# Peripheral and CSF protein quantification in Parkinson’s disease and multiple system atrophy—the nucleic acid-linked immuno-sandwich assay

**DOI:** 10.1093/braincomms/fcag035

**Published:** 2026-02-04

**Authors:** Nirosen Vijiaratnam, Christine Girges, Arthur Mitchell, Dilan Athauda, Riona Fumi, Jennifer Hay, Nicola O’Reilly, Huw Morris, Camille Carroll, Michele T M Hu, Monty A Silverdale, Gordon Duncan, Amanda Heslegrave, Eliza Chai, Sonia Gandhi, Thomas Foltynie

**Affiliations:** Department of Clinical and Movement Neurosciences, UCL Queen Square Institute of Neurology, London WC1N 3BG, UK; Department of Clinical and Movement Neurosciences, UCL Queen Square Institute of Neurology, London WC1N 3BG, UK; Neurodegenration Biology Laboratory, The Francis Crick Institute, London NW1 1AT, UK; Neurodegenration Biology Laboratory, The Francis Crick Institute, London NW1 1AT, UK; Department of Clinical and Movement Neurosciences, UCL Queen Square Institute of Neurology, London WC1N 3BG, UK; Neurodegenration Biology Laboratory, The Francis Crick Institute, London NW1 1AT, UK; Neurodegenration Biology Laboratory, The Francis Crick Institute, London NW1 1AT, UK; Department of Clinical and Movement Neurosciences, UCL Queen Square Institute of Neurology, London WC1N 3BG, UK; Translational and Clinical Research Institute Clinical Ageing Research Unit, Newcastle University, Newcastle, UK; Nuffield Department of CLinical Neurosciences, Oxford University and Oxford University Hospitals NHS Foundation Trust, Oxford OX3 9DU, UK; Manchester Centre for Clinical Neurosciences, Northern Care Alliance NHS Foundation Trust, Manchester Academic Health Science Centre, University of Manchester, Manchester M13 9NQ, UK; University of Edinburgh and Western General Hospital, Edinburgh EH4 2XU, UK; UK Dementia Research Institute, University College London, London NW1 3BT, UK; Department of Neurodegenerative Disease, UCL Institute of Neurology, London WC1N 3BG, UK; Alamar Biosciences, Inc, Fremont, CA 94538, USA; Department of Clinical and Movement Neurosciences, UCL Queen Square Institute of Neurology, London WC1N 3BG, UK; Neurodegenration Biology Laboratory, The Francis Crick Institute, London NW1 1AT, UK; Department of Clinical and Movement Neurosciences, UCL Queen Square Institute of Neurology, London WC1N 3BG, UK

**Keywords:** Parkinson’s disease, multiple system atrophy, biomarker, proteomics

## Abstract

There are currently no validated peripheral biomarkers for the diagnosis, differentiation or progression of the neurodegenerative synucleinopathies, Parkinson’s disease and multiple system atrophy. Diagnostic biomarkers that reflect the disease mechanisms or progression biomarkers that change with disease severity would be extremely valuable for assessing disease-modifying therapies. Our objective was to explore putative protein biomarkers of Parkinson’s disease and multiple system atrophy, in relation to clinical disease severity, using the nucleic acid-linked immuno-sandwich assay central nervous system disease panel for biomarker quantification. We used the nucleic acid-linked immuno-sandwich assay CNS disease panel to test plasma from 161 Parkinson’s disease patients collected at three time points (0, 48, 96 weeks) and serum from 43 multiple system atrophy patients at three time points (0, 24, 48 weeks) and compared results to paired plasma and serum samples collected from (*n* = 39) age-matched healthy control individuals at a single time point. We also tested paired CSF samples collected on two occasions, separated by 96 weeks from a subgroup of Parkinson’s disease participants (*n* = 51) and after an interval of 48 weeks in a subgroup of multiple system atrophy participants (*n* = 23). All samples were taken contemporaneously with objective clinical assessments of disease severity. Biomarker comparisons were made across disease status and in relation to disease severity using linear modelling. Multiple proteins showed significantly different quantitative levels (false discovery rate-corrected *P* value < 0.05) between peripheral samples from Parkinson’s disease and healthy controls and multiple system atrophy and healthy controls. For Parkinson’s disease, we identified three key classes of proteins that showed significant differences between Parkinson’s disease and controls: (i) amyloidogenic proteins, specifically, oligomeric alpha-synuclein was significantly higher in Parkinson’s disease compared to controls. A number of other aggregating proteins also exhibited differences. (ii) Metabolic pathways, including the adipokine (chemokine-like protein *TAFA-*5), were associated with Parkinson’s disease diagnosis, and (iii) inflammatory pathways (interleukin-7) were associated with Parkinson’s disease diagnosis. Importantly, some of these same proteins were significantly associated with Parkinson’s disease severity including oligomeric and phosphorylated forms of alpha-synuclein and insulin-like growth factor-1 receptor. We also confirmed as expected that neurofilament light levels strongly distinguish multiple system atrophy patients from healthy controls, while also demonstrating that serum inflammatory proteins (interleukin-6) as well as the phosphorylated alpha-synuclein ratio are strongly associated with multiple system atrophy severity. These results from the nucleic acid-linked immuno-sandwich assay multiplex platform provide additional insights into the complex pathogenetic mechanisms associated with alpha-synucleinopathy related neurodegeneration. Individual protein levels or the combination of multiple protein candidates may usefully serve as diagnostic biomarkers, or as biomarkers for disease progression in trials of potential disease-modifying interventions.

## Teaser

Vijiaratnam *et al.* report the results of the nucleic acid-linked immuno-sandwich assay panel detecting changes in levels of multiple proteins between Parkinson’s disease, multiple system atrophy and healthy controls in peripheral samples and CSF, highlighting the relevance of protein aggregation, inflammation and metabolism in the pathogenesis of these diseases.

## Graphical Abstract

**Graphical Abstract:**
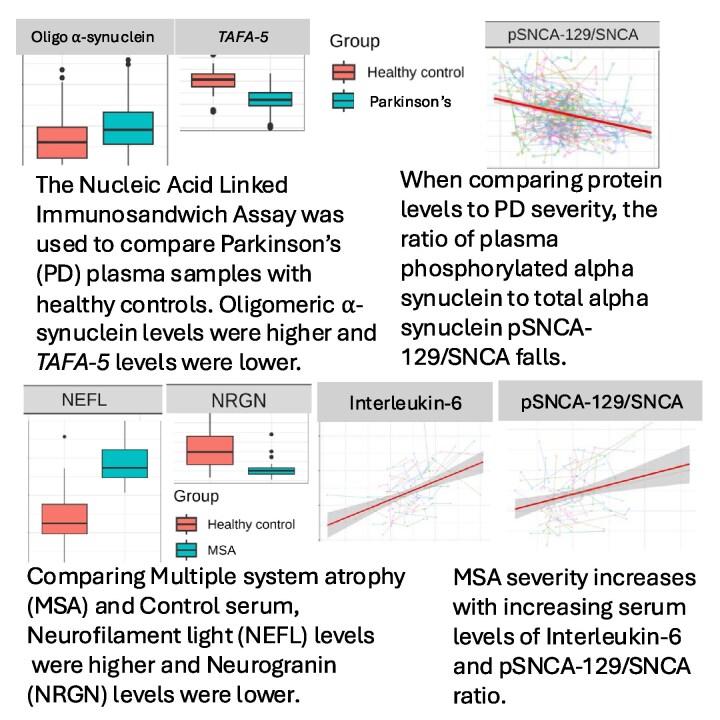

## Introduction

Parkinson’s disease and multiple system atrophy (MSA) are neurodegenerative diseases which have traditionally been defined based on the pathological confirmation of alpha-synuclein-positive Lewy body pathology^1^ and alpha-synuclein-positive glial cytoplasmic inclusions,^2^ respectively. Phenotypic differences have led to the development of clinical diagnostic criteria, but misdiagnosis can occur especially in the early years after disease onset; hence, there is a need for accurate and validated biomarkers for diagnostic confirmation.^3^

The pathophysiological processes leading to alpha-synuclein-related pathologies include alpha-synuclein oligomeric seeding, albeit with different pathological characteristics, which can be detectable using the alpha-synuclein seed amplification assay.^4,5^ Other common mechanisms leading to neurodegeneration include systemic and central nervous system (CNS) inflammatory responses, mitochondrial and lysosomal dysfunction,^6^ but a complete understanding of the range of pathophysiological processes contributing to neurodegeneration is lacking. Moreover, many individuals clinically diagnosed with Parkinson’s disease or MSA during life have multiple co-pathologies affecting the brain at post-mortem.^7^

Disease modification remains a key unmet goal in both of these disorders.^8^ Achieving this has been limited by our limited and oversimplistic understanding of disease pathophysiology and difficulty in distinguishing whether detectable responses to pathological processes act to compensate for toxicity, versus those which may aggravate/accelerate it, both of which are potential targets for disease-modifying interventions. To help track Parkinson’s disease progression over time, and in response to candidate interventions, a staging system based on the alpha-synuclein seed amplification assay, DaTSCAN™ imaging, and the severity of clinical features has been proposed^9^; however, a biomarker/combination of biomarkers, validated to be sensitive to changing disease severity, would shorten the duration of follow-up needed to assess the efficacy of an intervention in a clinical trial.

Proximity ligation assays can measure panels of (>100) CNS-related proteins in small sample volumes. These assays offer the possibility of broad explorations of multiple biomarker candidates in a single analysis, alongside comparisons between groups (or potentially even individuals) according to characterization of clinical phenotypes, and on a longitudinal basis. The nucleic acid-linked immuno-sandwich assay (NULISA) CNS panel with detection by next-generation sequencing (NULISAseq) is a novel approach created by Alamar Biosciences that can simultaneously measure proteins known to be involved in multiple pathophysiological pathways of relevance to neurodegeneration.^10-12^ The panel of CNS-related proteins (Supplementary Table 1) has been specifically chosen to capture recognized/candidate biomarkers of amyloid/tau pathologies, neurodegeneration, synuclein and synaptic disorders, inflammation and vascular and metabolism disorders. Previous studies have confirmed strong correlations between results produced using the NULISA platform compared with traditional single-molecule array (SIMOA) techniques.^12^

In this study, we aimed to explore the value of the NULISAseq 120-plex CNS disease panel in elucidating protein changes occurring in relation to neurodegeneration in Parkinson’s disease and MSA in comparison to age-matched healthy controls (HC), as well as their relationship to disease severity. We compared proteins in peripheral (serum/plasma) samples as well as central (CSF samples) collected longitudinally alongside systematic clinical data collection documenting disease severity, to Good Clinical Practice (GCP) trial standards.

## Materials and methods

### Participants and clinical assessments

Subjects for this study comprised participants with Parkinson’s disease from the exenatide PD3 trial,^13^ participants with MSA from the exenatide MSA trial^14^ and healthy controls (HC) from the PROSPECT-M-UK natural history study.^15^ The PROSPECT-M-UK study recruited participants with atypical parkinsonian disorders and HC to determine the clinical and biological natural history of these groups. Descriptions of the characteristics of these studies have previously been published.^13-15^ Patients with Parkinson’s disease were diagnosed using the Queen Square Brain Bank criteria,^16^ and patients with MSA were diagnosed using the Gilman criteria.^2^ All studies received research ethics committee approvals. In the Parkinson’s disease cohort, the MDS Unified Parkinson’s Disease Rating Scale Part 3 scores (MDS UPDRS Part 3) in the off-medication state were used as a measure of disease severity at baseline, 48 weeks and 96 weeks. For MSA patients, total Unified MSA Rating Scale (UMSARS) Part 1 + 2 scores were used to measure disease severity at baseline and after 24 and 48 weeks. All participants provided informed consent for sample collection and analysis.

### Sample collection

Parkinson’s disease blood samples collected at baseline, 48 weeks and 96 weeks from the exenatide PD3 trial were used in this study. The blood was centrifuged at 2000 × *g* for 10 min, and the plasma was stored in 1 ml aliquots at −80°C. For the MSA trial, blood samples were collected at baseline, 24 and 48 weeks and then processed to serum by centrifuging at 2000 × *g* for 10 min. A subgroup of consenting participants from both cohorts had 15 ml of CSF sample taken via lumbar puncture at baseline, and this was repeated at 96 weeks for the Parkinson’s disease trial and 48 weeks for the MSA trial. Plasma and serum from age-matched HC (collected from the same individuals simultaneously at the baseline visit of the PROSPECT-M-UK study) were used for this study (see Table 1). Only aliquots with no previous freeze-thaw cycles were used.

**Table 1:** Summary of cohort characteristics after exclusion of 1 outlying sample

	Healthy control (*N* = 78)	MSA-C (*N* = 20)	MSA-P (*N* = 22)	Parkinson’s disease (*N* = 161)	Overall (*N* = 281)
Sample matrix					
Plasma	39 (50%)	0 (0%)	0 (0%)	161 (100%)	200 (71.2%)
Serum	39 (50%)	20 (100%)	22 (100%)	0 (0%)	81 (28.8%)
Sex					
Female	44 (56.4%)	9 (45%)	12 (54.5%)	42 (26.1%)	107 (38.1%)
Male	34 (43.6%)	11 (55%)	10 (45.5%)	119 (73.9%)	174 (61.9%)
Age					
Mean (SD)	61.9 (13.0)	64.0 (8.32)	62.0 (7.96)	61.0 (9.17)	61.6 (10.2)
Median (min, max)	63.0 (33.0, 90.0)	61.2 (49.0, 78.1)	61.9 (49.6, 77.2)	61.2 (38.7, 77.5)	61.7 (33.0, 90.0)
MDS UPDRS Part 3 (off-drug)					
Mean (SD)	-	-	-	32.5 (13.0)	-
Median (min, max)				32.0 (8.0, 69.0)	
Hoehn and Yahr					
Stage 0	-	-	-	2 (1.2%)	-
Stage 1				14 (8.7%)	
Stage 2				133 (82.6%)	
Stage 3				11 (6.8%)	
Stage 4				1 (0.6%)	
UMSARS total					
Mean (SD)	-	42.9 (9.0)	44.4 (10.7)	-	-
Median (min, max)		44.0 (23.0, 56.0)	43.0 (28.0, 62.0)		
MoCA					
Mean (SD)	-	26.4 (2.3)	27.9 (1.6)	27.9 (1.9)	-
Median (min, max)		27.0 (22.0, 30.0)	28.0 (24.0, 30.0)	28.0 (22.0, 30.0)	

### NULISA testing

Samples were analysed using the NULISAseq CNS disease panel at Alamar Biosciences according to the manufacturer’s instructions. This technology provides quantification of 120 protein targets log transformed as NULISA protein quantification (NPQ) units.

Briefly, samples were thawed, centrifuged for 10 min at 10 000 × *g* and 4°C and transferred to 96-well plates and assayed with the reagent kit. Subsequent steps involved immunocomplex formation with the paired set of oligo-conjugated antibodies, first capture with oligo-dT beads, release, second capture with streptavidin beads and ligation performed on the automated Alamar ARGO prototype system. The library of DNA reporters containing unique target-specific molecular identifiers and sample-specific molecular identifiers was pooled, amplified by PCR, purified and sequenced on the Illumina NovaSeq X system.

After sequencing, automatic normalization (to each sample’s internal control, as well as intra-plate control) and conversion to the log_2_ scale NULISA protein quantification (NPQ) units were performed for each protein biomarker. Conversion to NPQ units is a method of linearizing fold change, reducing variance and improving interpretability (fold change = 2 ^Difference in NPQ^).

Samples were analysed in nine batches with 86 samples per plate. A pooled plasma control sample with three replicates was included on each plate to assess within-plate and between-plate reproducibility. To ensure consistency between batches, bridging normalization was performed using seven overlapping samples across different plates, consisting of three serum samples and four plasma samples. This method was applied to bridge two batches of plates, where each batch had already been internally normalized using inter-plate controls. For each target, the differences in NPQ were taken between the matching bridge samples. The target-specific median of the differences across all the bridge samples was then applied to the new batch for normalization. A protein target was considered detectable if the value was above the limit of detection (LOD) in >50% of samples, where LOD is three standard deviations above the mean of the unlogged normalized blank wells. NPQ differences between plates were calculated for each detectable target. Target-specific medians of differences were used to get normalization terms which were then added to each sample NPQ value. The ratios of pTau 217, pTau181 and pTau231 to MAPT, oligo-SNCA and pSNCA-129 to SNCA and Aβ42 to Aβ40 were derived from unlogged NPQ values, converted to log_2_ scale and included in all comparisons related to disease state and disease severity.

Prior to comparisons between phenotypic groups, outlier checking was performed for all samples using principal components analysis and unsupervised clustering on NPQ values after standardizing each target relative to group means and standard deviations.

### Statistical analysis

#### Peripheral biomarkers of disease state

To determine protein NPQ levels associated with disease status, plasma (Parkinson’s disease) samples and serum (MSA all cases, MSA-P subgroup, MSA-C subgroup) collected at baseline visits were compared to plasma/serum samples respectively from HC. Direct comparisons were also made between the serum and plasma samples collected contemporaneously from HC individuals, to inform on the comparability of NPQ levels according to these different matrices. This served to determine whether proteins specifically associated with Parkinson’s disease plasma compared to MSA serum would be possible. Pairwise comparisons were performed combining protein expression of all samples collected at baseline visits, in a categorical linear model adjusting for age at baseline and sex.

#### CSF biomarkers

Protein NPQ levels in CSF from Parkinson’s disease and MSA individuals were compared using pairwise comparisons combining protein expression of all samples from baseline collections in a categorical linear model adjusting for age and sex.

#### Biomarkers of disease severity

To determine which proteins were associated with disease severity, we performed linear mixed-effects regression modelling, where disease severity score across all time points was modelled as a function of protein NPQ levels, adjusting for age at baseline, sex and exenatide drug randomization, and a random effect for subject to account for within-subject correlation. To control for multiple comparisons, the threshold for statistical significance was determined using the Benjamini–Hochberg method to adjust for the false discovery rate (FDR), with a significance level set at adjusted *P* < 0.05.

## Results

### Cohort characteristics and quality control

Baseline characteristics of the different phenotypic groups are summarized in Table 1.

A total of 161 Parkinson’s disease patients provided plasma samples (from three time points), and 43 MSA patients (21 with MSA-C, 22 with MSA-P) provided serum samples (from three time points). Peripheral samples taken at baseline (prior to exenatide exposure) were compared to paired plasma (Parkinson’s disease analysis) and serum (MSA analysis) samples respectively collected from (*n* = 39) HC at a single time point. A total of 51 Parkinson’s disease patients provided two CSF samples each (at baseline and after 96 weeks), and 23 MSA patients (10 MSA-C, 13 MSA-P) provided two CSF samples each (at baseline and after 48 weeks).

A total of 830 samples (526 plasma, 156 serum and 148 CSF) were profiled in two batches. In the first batch, the across-target median of the per-target average intra-plate per cent coefficient of variation (%CV) was 6.2% (expected range <10%). The median inter-plate %CV was 8.5% (expected range <15%). In the second batch, the intra-plate %CV median was 5.5%. Approximately 95% of targets were detectable in Parkinson’s disease plasma, and 93.5% were detectable in MSA serum. In addition, 103 (83.5%) were detectable in CSF across both diseases. (Targets with detectability <50% in plasma, serum or CSF samples are listed in Supplementary Table 2.) Unlike all other targets, APOE4 status is considered binary ‘carrier or non-carrier’ rather than on a continuous scale.

One outlying sample (MSA- C serum) was identified through PCA and heat map clustering showing NPQ values substantially lower than the group mean for the majority of targets. This sample and targets with detectability < 50% were removed from subsequent analysis.

### Differential protein expression between Parkinson’s disease and HC

**Figure 1:**
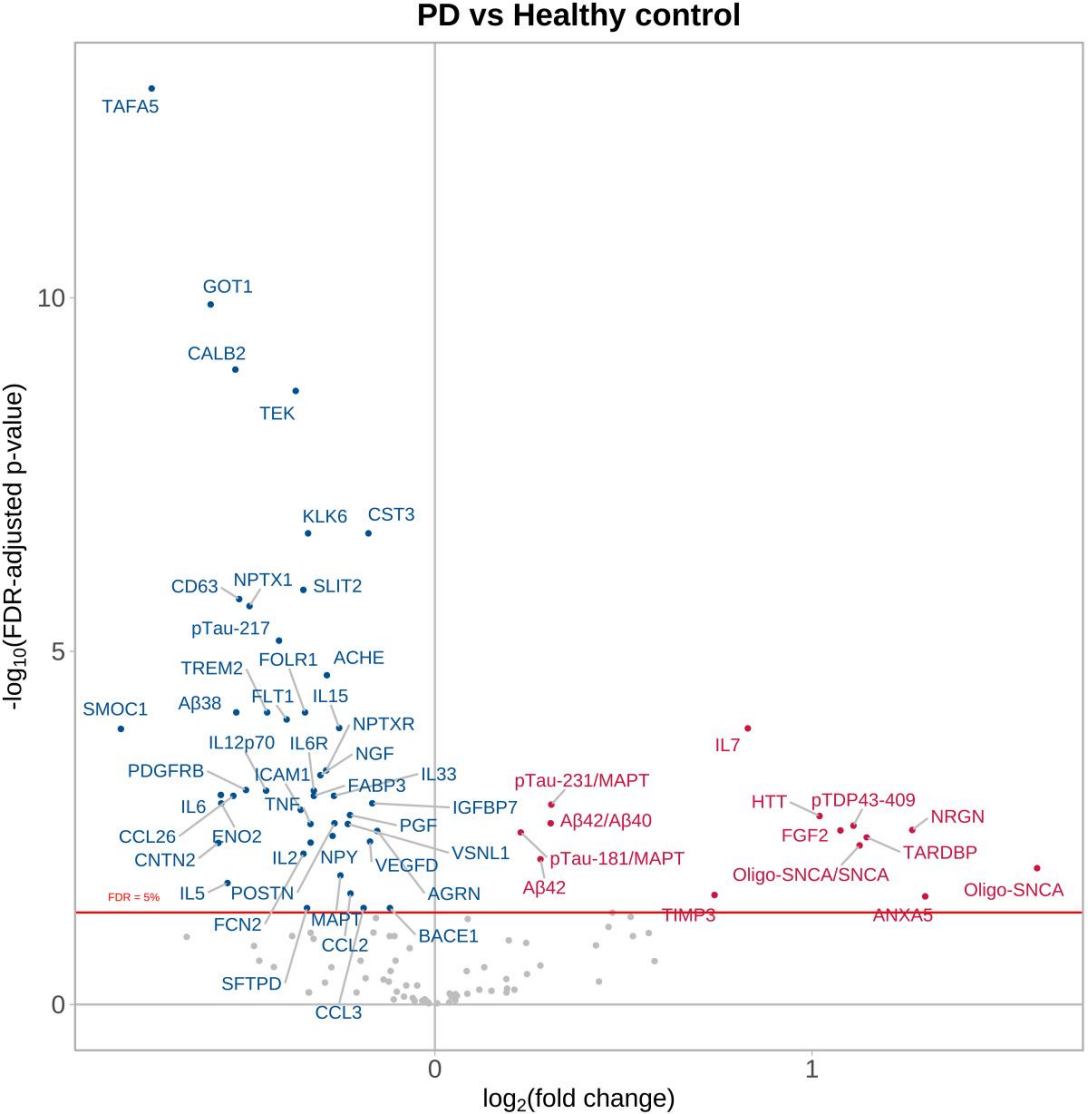
**Parkinson’s disease plasma versus healthy control plasma.** Parkinson’s disease plasma samples (*n* = 161) taken at baseline trial visits compared to healthy control plasma samples (*n* = 39) using a categorical linear model adjusting for age at baseline and sex. The volcano plot shows log_10_-adjusted FDR-adjusted *P* values represented on the *y*-axis, i.e. a log_10_-adjusted FDR-adjusted *P* value of 5 represents *P* = 0.00001. The *x*-axis shows the log_2_ fold change, i.e. positive fold changes (proteins displayed in red) of +1 indicate that the protein values are twice as high in the Parkinson’s disease samples compared with control samples. See Supplementary material for the abbreviation list.

Figure 1 (Supplementary Figs 1 and 2) summarizes the target proteins identified by the models as having significantly different NPQ levels (FDR-adjusted *P* value < 0.05) between Parkinson’s disease plasma and HC plasma. The log_2_ (fold change) plotted on the *x*-axis indicates how much the protein levels change, i.e. a log_2_ score of 1 equates to a 2,^1^ i.e. a 2× higher level of that protein. The *y*-axis shows the *P* values on a −log_10_ scale, i.e. a *P* value of 0.0001 equates to a −log_10_ score of 4.

A total of 45 proteins were significantly reduced in Parkinson’s disease plasma compared to HC plasma, while 10 proteins were significantly increased. Notably, oligo-SNCA, ANXA5, NRGN, TARDBP, oligo-SNCA/SNCA ratio, pTDP43-409 and HTT had log_2_ fold change scores of 1 (i.e. twice the absolute level), whereas the greatest reduction in NPQ levels in Parkinson’s disease patients compared to healthy controls was for TAFA5, GOT1, CALB2, CD63, Aβ38 and SMOC1. The ratios of pTau-231/MAPT, pTau-181/MAPT and Aβ42/Aβ40 were also significantly elevated in Parkinson’s disease patients.

### Proteins associated with Parkinson’s disease severity

**Figure 2:**
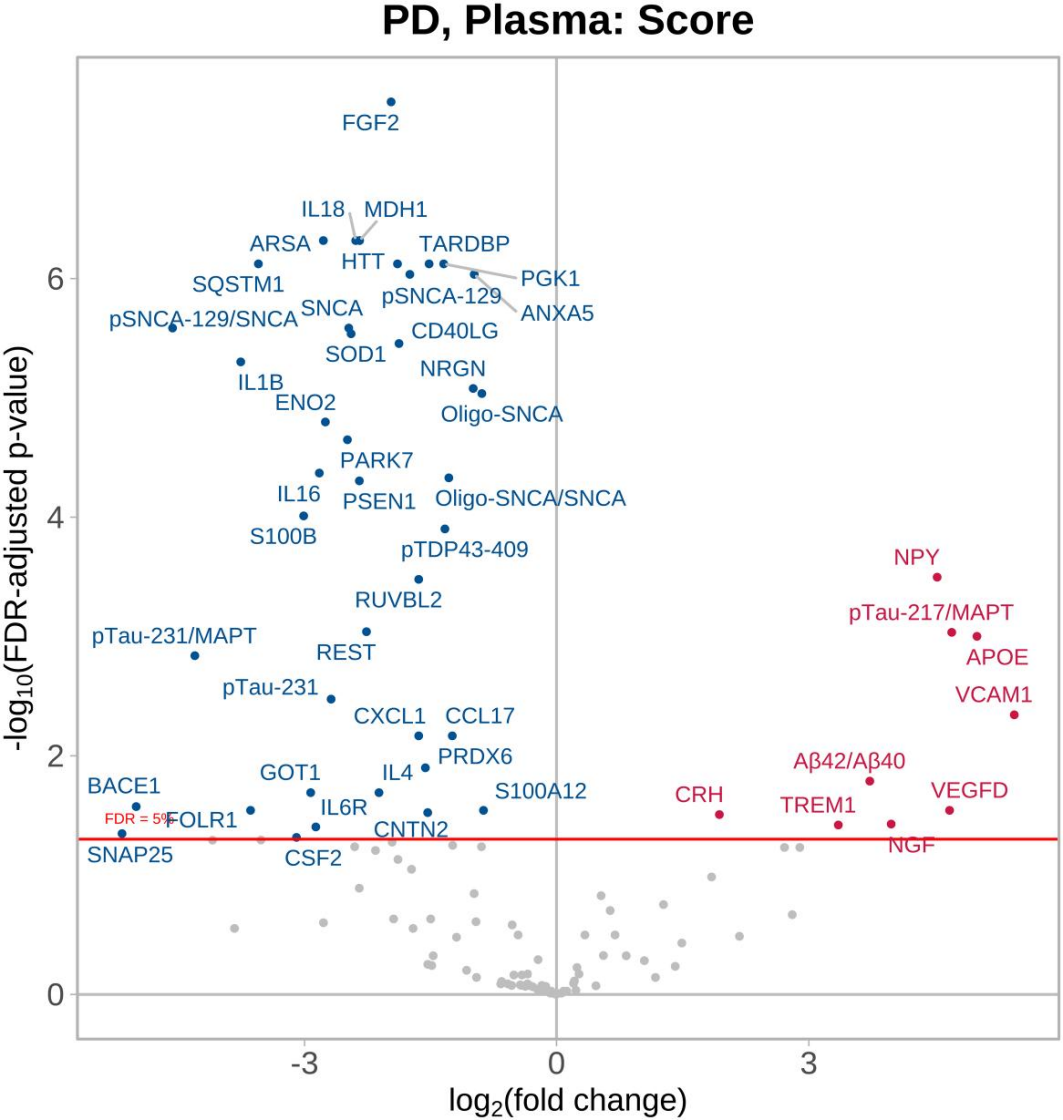
**Parkinson’s disease plasma versus Parkinson’s disease severity.** Parkinson’s disease plasma samples (*n* = 161) collected at multiple time points and compared to Parkinson’s disease severity using linear mixed-effects regression modelling, adjusting for age at baseline, sex and exenatide drug randomization, and a random effect for subject to account for within-subject correlation. The volcano plot shows log_10_-adjusted FDR-adjusted *P* values represented on the *y*-axis, i.e. a log_10_-adjusted FDR-adjusted *P* value of 5 represents *P* = 0.00001. The *x*-axis shows the direction of effect, i.e. positive coefficients (proteins displayed in red) indicate that higher protein values are significantly associated with worse disease severity. See Supplementary material for the abbreviation list.

Based on data from all three time points, 49 plasma proteins had NPQ levels that were significantly associated with Parkinson’s disease severity. NPY, pTau217/MAPT, APOE, VCAM1, VEGFD, TREM1, Aβ42/Aβ40, NGF and corticotropin-releasing hormone (CRH) were the strongest positively associated proteins (higher levels with increasing disease severity), whereas the most strongly negatively associated proteins were FGF2, MDH1, IL18, ARSA, SQSTM1 pSNCA-129/SNCA and IL1B (Fig. 2 and Supplementary Fig. 3).

**Figure 3:**
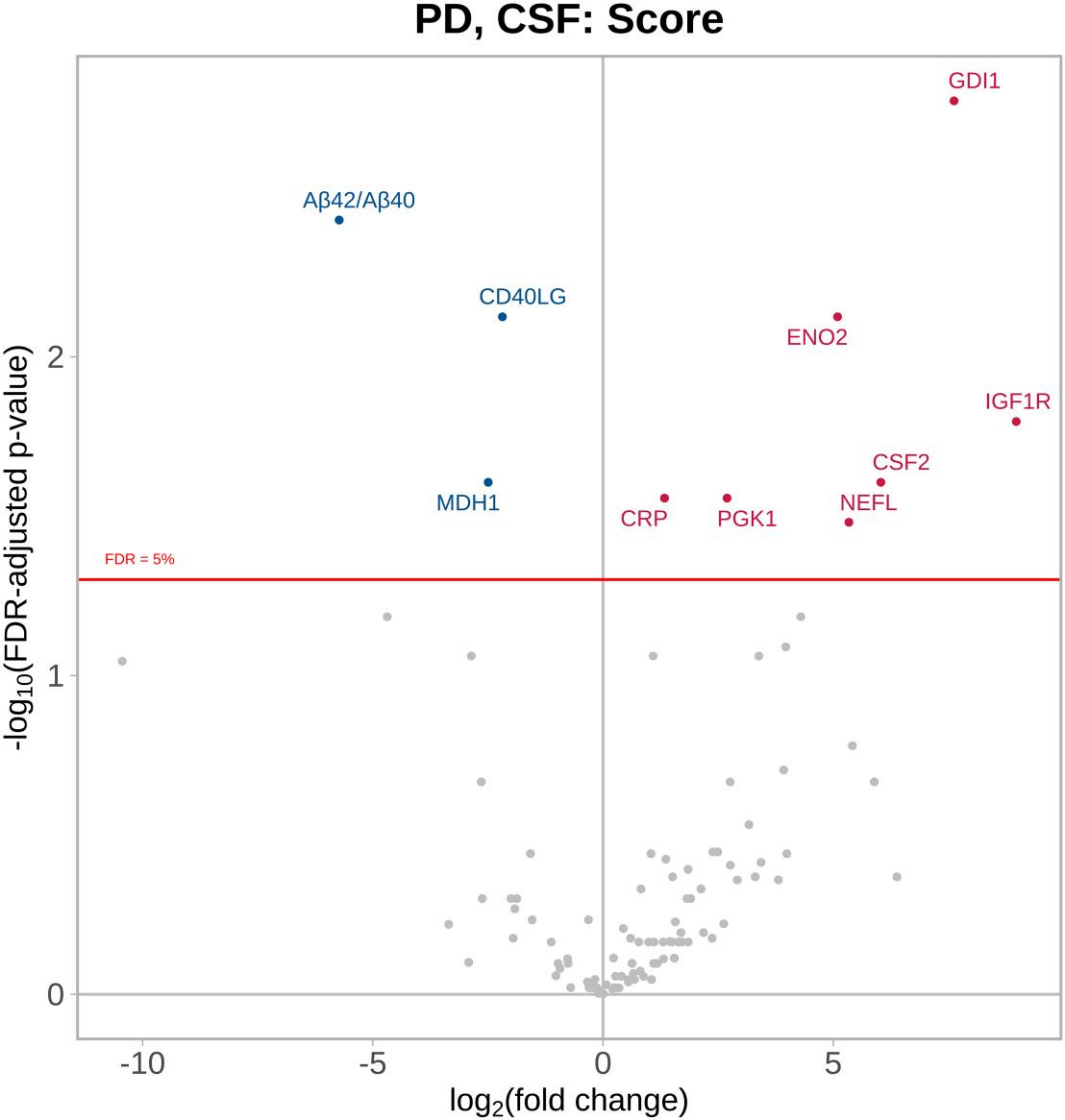
**Parkinson’s disease CSF versus Parkinson’s disease severity.** Parkinson’s disease CSF samples (*n* = 51) collected at multiple time points and compared to Parkinson’s disease severity using linear mixed-effects regression modelling, adjusting for age at baseline, sex and exenatide drug randomization, and a random effect for subject to account for within-subject correlation. The volcano plot shows log_10_-adjusted FDR-adjusted *P* values represented on the *y*-axis, i.e. a log_10_-adjusted FDR-adjusted *P* value of 2 represents *P* = 0.01. The *x*-axis shows the direction of effect, i.e. positive coefficients (proteins displayed in red) indicate that higher protein values are significantly associated with worse disease severity. See Supplementary material for the abbreviation list.

In the CSF of Parkinson’s disease patients, 10 proteins were significantly associated with disease severity over the two time points; notably higher NPQ levels of GDI1, IGF1R, ENO2, CSF2, NEFL and PGK1 were associated with more severe disease, whereas Aβ42/Aβ40, CD40LG and MDH1 were associated with less severe disease (Fig. 3 and Supplementary Fig. 4).

### Differential protein expression between MSA and HC

**Figure 4:**
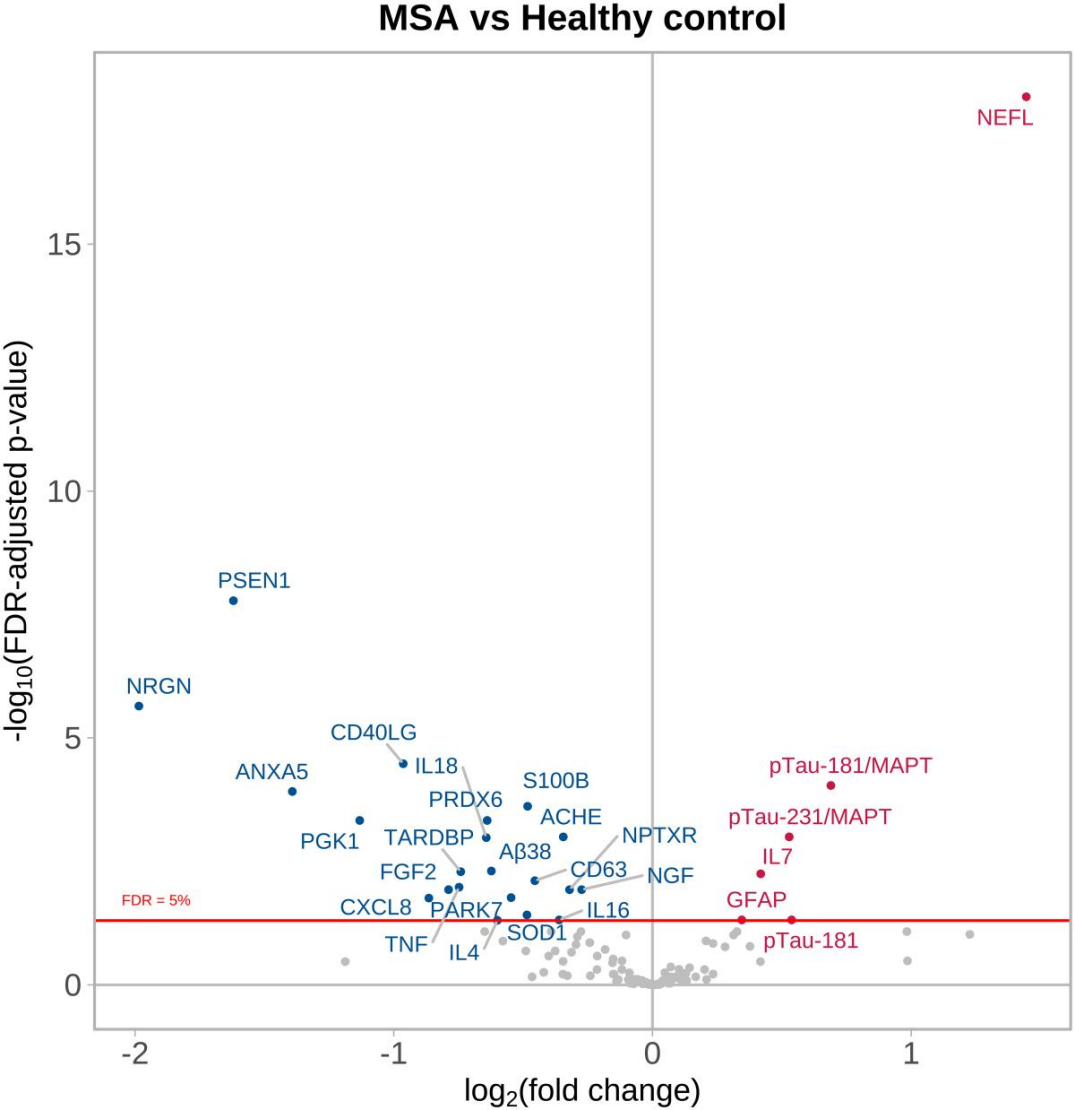
**MSA serum versus healthy control serum.** MSA serum samples (*n* = 42) taken at baseline trial visits compared to healthy control serum samples (*n* = 39) using a categorical linear model adjusting for age at baseline and sex. The volcano plot shows log_10_-adjusted FDR-adjusted *P* values represented on the *y*-axis, i.e. a log_10_-adjusted FDR-adjusted *P* value of 5 represents *P* = 0.00001. The *x*-axis shows the log_2_ fold change, i.e. positive fold changes (proteins displayed in red) of +1 indicate that the protein values are twice as high in the MSA samples compared with control samples. See Supplementary material for the abbreviation list.

Figure 4 and Supplementary Fig. 5 show four target proteins having significantly increased NPQ levels in MSA serum compared with HC serum (NEFL, IL7, GFAP and pTau-181), alongside the ratios of pTau181/MAPT and pTau231/MAPT, while 21 proteins were significantly decreased (Fig. 4 and Supplementary Fig. 6), with NRGN, PSEN1, ANXA5 and PGK1 being the most significantly and substantially decreased.

Supplementary Figs 7–12 show the comparisons restricted to MSA-P and MSA-C, respectively, showing consistency of NEFL increases and NRGN, PSEN1, ANXA5 and PGK1 decreases in both phenotypes. IL-7 and GFAP appear to be differentially increased in MSA-C individuals, whereas the ratios of pTau181/MAPT and pTau231/MAPT and POSTN were differentially elevated in MSA-P individuals.

### Proteins associated with MSA severity

**Figure 5:**
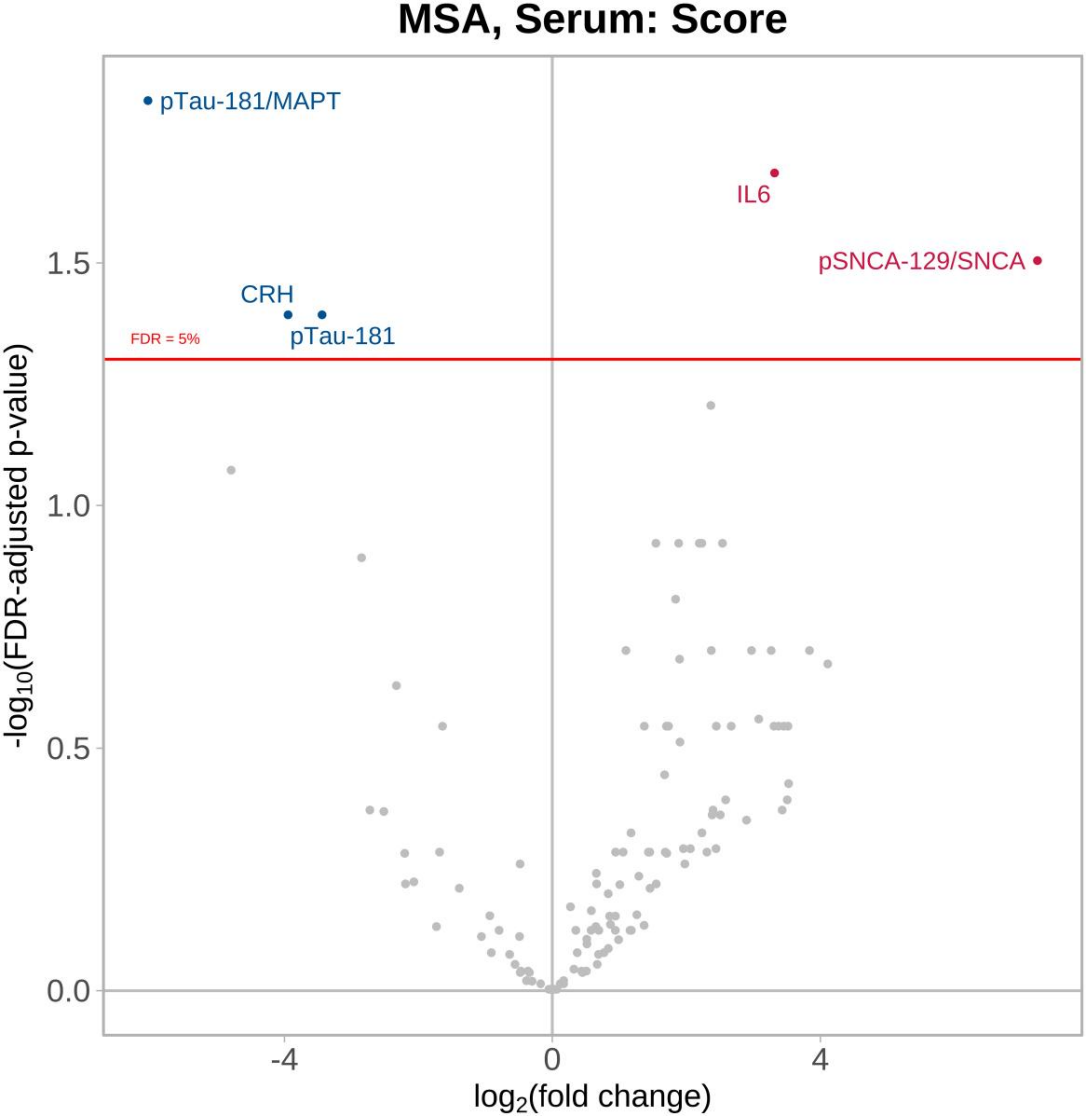
**MSA serum versus MSA severity.** MSA serum samples (*n* = 42) collected at multiple time points and compared to MSA severity using linear mixed-effects regression modelling, adjusting for age at baseline, sex and exenatide drug randomization, and a random effect for subject to account for within-subject correlation. Volcano plot showing log_10_-adjusted FDR-adjusted *P* values represented on the *y*-axis, i.e. a log_10_-adjusted FDR-adjusted *P* value of 2 represents *P* = 0.01. The *x*-axis shows the direction of effect, i.e. positive coefficients (proteins displayed in red) indicate that higher protein values are significantly associated with worse disease severity. See Supplementary material for the abbreviation list.

In the comparison of NPQ protein levels and MSA disease severity across all corresponding time points, four proteins were significantly associated; notably pSNCA-129/SNCA and IL6 were most associated with higher UMSARS 1 + 2 scores (increased severity) while pTau181/MAPT and CRH were most significantly associated with reduced severity (Fig. 5 and Supplementary Fig. 13). In the MSA-P subgroup, pTau181/MAPT was associated with worse disease severity (Supplementary Figs 14 and 15), while pSNCA-129/SNCA as well as C–C chemokine 4 (CCL4), CCL3, CCL22, CRP and FGF2 were all associated with worsening severity in the MSA-C subgroup (Supplementary Figs 16 and 17). CRH was associated with reduced MSA severity in the MSA-C subgroup.

There were no CSF proteins which significantly predicted MSA severity at the FDR threshold, nor in analyses separately exploring the smaller subgroups of MSA-C and MSA-P.

### Peripheral biomarker comparisons between Parkinson’s disease and MSA

Comparisons of differential expression according to the serum/plasma matrices in identical HC (samples taken contemporaneously from the same healthy individuals) revealed differences across multiple proteins (Supplementary Figs 18 and 19), indicating that the matrix itself can lead to substantial differences in NPQ levels, thus preventing any meaningful comparisons between Parkinson’s disease ‘plasma’ samples and MSA ‘serum’ samples.

### CSF biomarker comparisons between Parkinson’s disease and MSA

**Figure 6:**
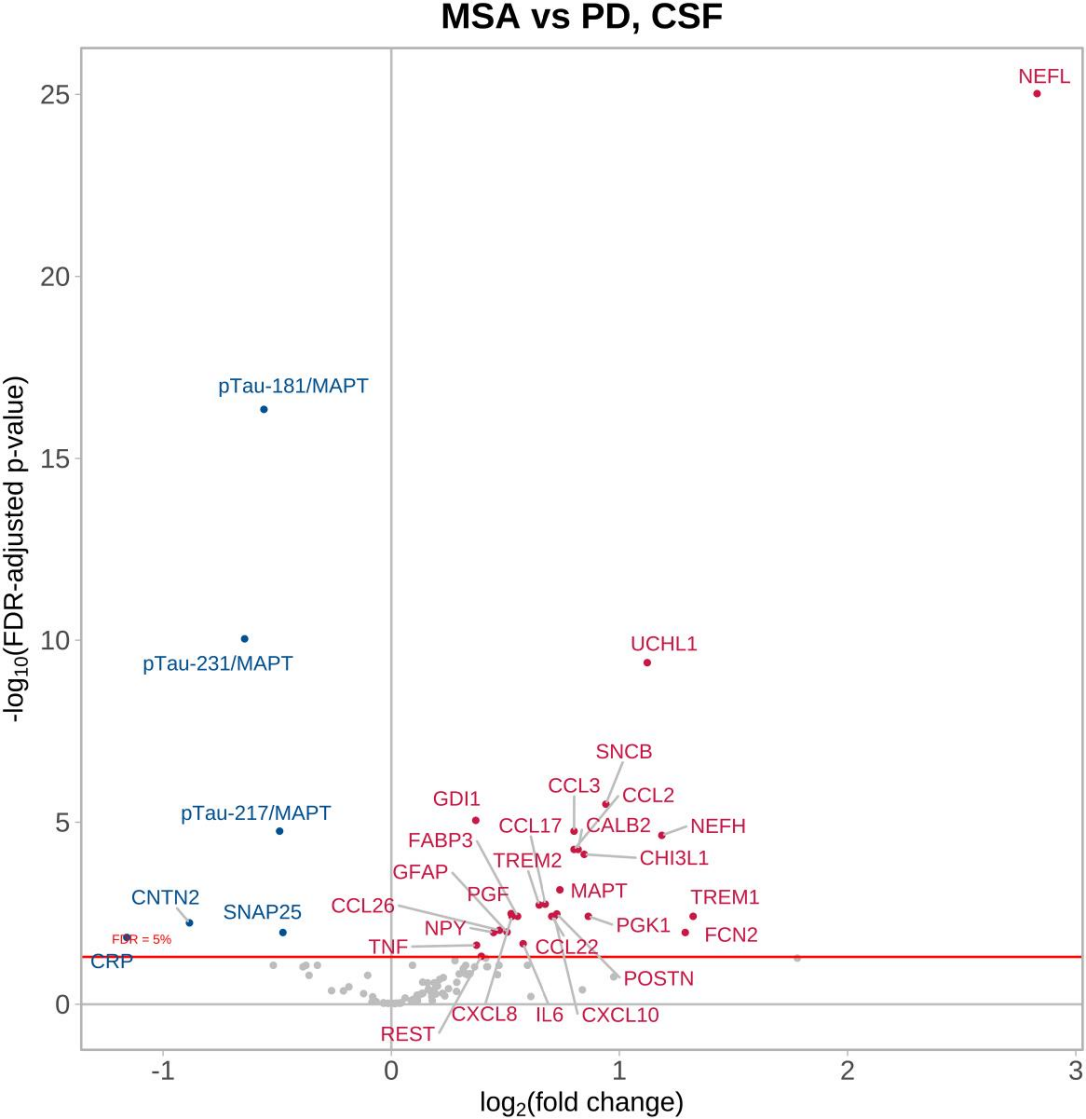
**Parkinson’s disease CSF versus MSA CSF.** Magnitude of differential protein expression in MSA compared to Parkinson’s disease in CSF using a categorical linear model adjusting for age at baseline and sex. Parkinson’s disease CSF samples (*n* = 51) compared to MSA CSF samples (*n* = 23) collected at trial baseline visits. The volcano plot shows log_10_-adjusted FDR-adjusted *P* values represented on the *y*-axis, i.e. a log_10_-adjusted FDR-adjusted *P* value of 5 represents *P* = 0.00001. The *x*-axis shows the log_2_ fold change, i.e. positive fold changes (proteins displayed in red) of +1 indicate that the protein values are twice as high in the MSA samples compared with Parkinson’s disease samples. See Supplementary material for the abbreviation list.

Comparison of protein expression in Parkinson’s disease versus MSA CSF samples revealed 27 significantly increased proteins in MSA (of which NEFL, NEFH, UCHL1, TREM1 and FCN2 had greater than 2-fold change at FDR-adjusted thresholds for statistical significance) (Fig. 6 and Supplementary Fig. 20). This was very similar in a comparison between MSA-P and Parkinson’s disease CSF (Supplementary Figs 21 and 22) whereas in the comparison between MSA-C and Parkinson’s disease CSF samples, there were also greater than 2-fold increases in SNCB, MAPT, CALB2, CCL3, CHI3L1, TREM1, TREM2, MDH1 and PGK1 in MSA-C patients compared to Parkinson’s disease CSF (Supplementary Figs 23 and 24).

The largest decreases seen in MSA CSF compared with Parkinson’s disease CSF were for pTau181/MAPT, pTau231/MAPT and pTau217/MAPT (Fig. 6 and Supplementary Fig. 25) as well as CNTN2 (MSA-P) (Supplementary Figs 21 and 26) and SNAP2 (MSA-C) (Supplementary Figs 23 and 27).

## Discussion

To date, this study is the most comprehensive evaluation of peripheral and CNS protein changes to have been performed in Parkinson’s disease and MSA patients in comparison to healthy control subjects as well as an evaluation of protein expression compared against systematic documentation of disease severity. The strength of this study lies in the selection of a NULISA panel of 120 CNS proteins that captures key drivers of synucleinopathy mechanisms (aggregation, metabolism, inflammation and synaptic loss) in a large sample size.

### Which proteins differ the most between Parkinson’s disease plasma and HC?

#### Protein misfolding is the central pathogenic driver of all neurodegenerative diseases

There has been extensive validation of the seed amplification assay for alpha-synuclein from Parkinson’s disease CSF^17^ and skin,^18^ and there is ongoing work to create a validated equivalent assay in peripheral blood samples^19^ meaning that the seed amplification assay is likely to be the gold standard to differentiate neuronal alpha-synuclein pathology from other neurodegenerative disorders such as MSA/PSP.^9^ The use of the seed amplification assay has not yet been shown however, to be useful in measuring disease severity.

#### Alpha synuclein

Importantly, given that we are defining our populations as having an alpha-synucleinopathy, we found significantly elevated levels of oligomeric SNCA in Parkinson’s disease plasma samples compared to HC in the group comparisons. When normalizing for total SNCA (using oligo-SNCA/total SNCA), the positive association with Parkinson’s disease state was preserved. We did not find evidence of elevated pSNCA-129 (phosphorylated alpha-synuclein at Position 129) nor total SNCA in Parkinson’s disease plasma samples. pSNCA-129 is the pathological form of alpha-synuclein that is deposited in Lewy bodies and glial cytoplasmic inclusions of MSA.^20^ Despite the importance of these abnormal forms of alpha-synuclein in the brain, for Parkinson’s disease diagnosis, they are not sufficiently strong enough to be a plasma biomarker.

With relevance to alpha-synuclein turnover, we found that levels of KLK6 (kallikrein-related peptidase 6, also known as neurosin) are lower in Parkinson’s disease plasma compared to HC. KLK6 is expressed within neurons and oligodendrocytes and is a protease which is able to cleave alpha-synuclein and protect against alpha-synuclein aggregation.^21-23^ This protein also has an inverse correlation with alpha-synuclein load in dementia with Lewy body (DLB) patients at post-mortem^24^ but seemingly does not co-locate with alpha-synuclein deposition in MSA.^25^

The elevated levels of plasma ANXA5 are also of interest. ANXA5 (annexin A5) interacts and reduces the amyloidogenic toxicity of alpha-synuclein^26^ and has been previously found to be decreased in the CSF of Parkinson’s disease patients compared to controls.^27^

#### Elevated levels of aggregating proteins related to other neurodegenerative diseases

The role of other aggregating proteins in Parkinson’s disease remains unclear, although there appears to be extensive interrelationships between brain proteins related to neurodegeneration due to perturbations in protein homeostasis and or intracellular changes resulting from protein aggregation,^28^ with evidence that co-pathology with other aggregating proteins is almost the rule rather than the exception in neurodegenerative disease.^7,29,30^

Here, we detected significantly elevated plasma levels of TDP43, TDP43-p409 and HTT in Parkinson’s disease plasma compared to HC. We also found a minor, although still statistically significant, increase in Aβ42, pTau231/MAPT and pTau181/MAPT, alongside reduction of Aβ38, pTau-217 and MAPT. While originally described in association with Alzheimer’s disease, these amyloid and tau plasma biomarkers do not have high specificity for Alzheimer’s disease when compared to Lewy body dementia^31^ or Parkinson’s disease^32^ and are thought to indicate concurrent pathologies.

In Huntington’s disease brains, reduced soluble protein levels of HTT, alpha-synuclein and tau are all seen, and aggregations of alpha-synuclein correlate with HTT, TDP-43 and pTau levels in Huntington’s disease patients.^33^ Indeed, up to 82% of elderly Huntington’s disease patients have concurrent Alzheimer’s disease pathology at post-mortem^34^ and tau pathology (corticobasal degeneration/PSP) has previously been seen in combination with polyglutamine aggregates.^35^ Similarly, combinations of amyloid, tau, TDP-43 and alpha-synuclein can be seen in patients with chronic traumatic encephalopathy.^36^

Our findings of elevated plasma levels of HTT and TDP43/TDP43-409 in patients with Parkinson’s disease provide further support for the existence of interactions amongst numerous amyloidogenic proteins, even at a scale that may not lead to insoluble protein deposition at post-mortem (although TDP43 inclusions have been previously described in LRRK2-positive Parkinson’s disease cases).^37^

#### Markers of metabolic dysfunction

The most significant difference between Parkinson’s disease and HC plasma was a marked reduction in TAFA-5 in Parkinson’s disease plasma. TAFA-5 (recently renamed FAM19A5) is a chemokine-like protein with a role in the regulation of the immune response in the brain^38^ as well as being an adipokine with a role in homeostasis, and a marker for Type 2 diabetes and elevated blood glucose.^39^ The marked lowering of this protein in Parkinson’s disease contrasts with the findings of a previous study^40^ (which showed significant increases in FAM19A5 in fasted Parkinson’s disease patients with depression); however, it is of major interest given the emerging strong links between Parkinson’s disease neurodegeneration, insulin resistance and neuroinflammation.

GOT1 (glutamic-oxaloacetic transaminase 1) has a role in cancer proliferation due to its role in amino acid biosynthesis. It acts as a scavenger for glutamate.^41^ Consistent with our findings, it has previously been shown to be one of the seven genes which are downregulated in an analysis of the Gene Expression Omnibus database in Alzheimer’s disease, Parkinson’s disease and Huntington’s disease and has been proposed as a potentially druggable target in neurodegeneration with 1 or more nutritional supplements.^42^

Calbindin 2 (also known as calretinin) is a calcium-binding protein involved in intracellular calcium buffering. Loss of calcium-binding proteins has been described as an early feature of Parkinson’s disease that cannot be explained purely as a consequence of neurodegeneration,^43^ and it has been suggested as a potential neuroprotective protein for neurons in the substantia nigra,^44,45^ possibly mediating the relationship between oestrogen and the PI3K/Akt signalling pathway.^46^

#### Markers of neuroinflammation in Parkinson’s disease

Neuroinflammation is well established as relevant to alpha-synuclein-related neurodegeneration. The NULISA panel includes multiple neuroinflammatory proteins allowing us to further evaluate previously described relationships in Parkinson’s disease compared to HC and in relation to disease severity. Increased peripheral blood levels of IL-6, TNF-α, IL-1β, STNFR1, CRP, CCL2, CX3CL1, CXCL12 and VCAM1 have all previously been associated with Parkinson’s disease alongside decreases in IFN-γ and IL-4 levels in a systematic review of neuroinflammatory biomarkers, with similar patterns seen in CSF, i.e. elevations in IL6, TNF1, IL1B and CRP in Parkinson’s disease patients compared to HC.^47^

In contrast, however, our data found reduced levels of IL-6, IL-12p70, IL6R, IL-15, TNF-α, CCL2 and CCL3 in plasma from Parkinson’s disease patients versus HC, while we found significantly elevated levels of IL-7 in Parkinson’s disease patients’ plasma.

IL-7 is a member of the IL-2 interleukin subfamily which is essential for balancing the pro-inflammatory and anti-inflammatory aspects of the immune response mediated through the actions of regulatory T cells. IL-7 is a T-cell-associated cytokine which is essential for normal T-cell development and forms part of a homeostatic response for maintaining T-cell populations by inhibiting their apoptosis and when dysregulated can lead to the promotion of haematological cancers and solid tumours.^48^ IL-7 also supports aberrant immune activity in autoimmune diseases such as Type 1 diabetes^49^and in chronic inflammatory diseases such as inflammatory bowel disease.^50^ While IL-7 is expressed in the brain by neuronal progenitor cells,^51^ our finding of greatly elevated levels of IL-7 in Parkinson’s disease plasma versus HC, while not being related to disease severity, requires further exploration to distinguish whether this is a compensatory or causative phenomenon in Parkinson’s disease pathogenesis.

Lower levels of IL-6 and CCL-2 were detected in Parkinson’s disease plasma compared with HC. IL-6 and IL-15 are pro-inflammatory cytokines secreted by macrophages in response to pathogen-associated molecular patterns (PAMPs), leading to the acute phase response. The participants in this study were of representative age (61.0 years) of the population of Parkinson’s disease patients, albeit with an excess of male participants. The comparison with HC used Parkinson’s disease samples from the baseline trial visit, prior to any trial drug exposure, which excludes exenatide as a possible explanation for the lower levels of these inflammatory proteins. All participants were however also on L-dopa replacement which has been reported to have anti-inflammatory actions^52-54^ and might account for some of the differences in the diversity of results across studies of inflammatory proteins in Parkinson’s disease.

Other studies including large numbers of Parkinson’s disease CSF samples^55,56^ have also found no difference in IL6, CRP or CCL2 between Parkinson’s disease and healthy control CSF samples. The neuroinflammatory response is highly dynamic, and feedback mechanisms exist that may vary in their extent and effectiveness between patients in response to varying disease states. Furthermore, our view of these interleukins and chemokines may be somewhat simplistic, and their impact on neuronal biology may vary not only between patients but also over time according to changes in the expression of their intracellular targets.

Of further relevance is the highly significant relationship between lower levels of TEK and Parkinson’s disease. TEK receptor tyrosine kinase is expressed in endothelial cells and is the cell surface receptor for angiopoietin preventing the leakage of pro-inflammatory proteins into the CNS. Angiopoietin 2 has previously been found to rescue damaged dopaminergic neurons in adult rodents.^57^

### Which proteins are associated with disease severity in Parkinson’s disease?

Our longitudinal experimental design includes multiple time points from the same Parkinson’s disease patients, enabling us to determine whether any of the CNS NULISA panel proteins varied with worsening severity.

#### CSF protein changes with Parkinson’s disease severity

In the CSF of Parkinson’s disease patients, GDI1, IGF1R, NEFL, ENO2, CSF2 and PGK1 were directly associated with increased motor severity of disease. NEFL is the most well-established marker of axonal damage that has been repeatedly shown to be elevated in the CSF in the context of atypical forms of parkinsonism as well as a large number of other neurodegenerative diseases. Plasma NEFL levels are known not to be particularly elevated in Parkinson’s disease however, and this is less likely to be of use in Parkinson’s disease diagnosis although the relationship between Parkinson’s disease severity and NEFL suggests a potential role for its use in disease-modifying trials with sufficiently long-term follow-up.

GDI1 (Rab GDP dissociation inhibitor alpha) regulates the normal exchange of GDP/GTP of Rab proteins and is potentially of relevance to Parkinson’s disease and normal synaptic vesicle transport.^58^ GDI1 has previously been reported to be elevated in the CSF of Alzheimer patients and has been proposed to be a marker of microglial activation in response to beta amyloid.^59^ ENO2 (gamma enolase) is also well recognized as a biomarker for neuroinflammation and neuronal function, serving a role in the PI3k/Akt pathway, and also a mediator of cathepsin X-mediated degeneration.^60^

Notably, we found that elevated CSF levels of the IGF1 receptor were associated with increased severity of Parkinson’s disease. None of the participants in these trials had diabetes, but it is increasingly clear that there is a relationship between central insulin resistance and neurodegeneration in Parkinson’s disease.^61^ Insulin signalling is thought to have beneficial effects on cell survival, also through the PI3k/Akt pathway,^61^ and we would hypothesize that elevated IGF1R levels are a compensatory response to a reduction in IGF-1 itself rather than being causally related to neurodegeneration.

#### Plasma protein changes with Parkinson’s disease severity

While oligo-SNCA was found to be significantly elevated in Parkinson’s disease plasma compared to HC, it was negatively associated with disease severity. Notably, all three measured subtypes of alpha-synuclein (oligo-SNCA, pSNCA-129 and total SNCA, as well as the ratios of oligo-SNCA/SNCA and pSNCA-129/SNCA) were reduced in the plasma in association with greater disease severity, potentially a reflection of deposition of alpha-synuclein aggregates either peripherally or centrally as Lewy bodies.^62^ There were no significant changes in the CSF levels of any alpha-synuclein subforms in relation to disease severity. The potential of measuring plasma pSNCA-129/SNCA as a biomarker for Parkinson’s disease severity has previously been reviewed.^63,64^

A strong positive association was seen between plasma NPY levels and Parkinson’s disease severity. Increased numbers of cells expressing NPY have previously been reported in Parkinson’s disease patients,^65^ and given the neuroprotective effects of NPY in animal models,^66^ this has been proposed to be an endogenous neuroprotective response to the neurodegenerative process.^67^

Strongly significant inverse associations with Parkinson’s disease severity were also seen with plasma levels of FGF2, SQSTM1, MDH1, HTT, IL18, ANXA5, CD40LG, PGK1 and ARSA. While FGF2 is involved in neuroinflammation with an influence on microglial activation,^68^ it is also a growth factor with pleiotropic effects including adult neurogenesis^69,70^ and synaptic plasticity^71^ and is neuroprotective in rodent models of Parkinson’s disease.^72-74^ FGF2 has previously been the source of interest as a potential disease-modifying approach in Parkinson’s disease but represents a challenge regarding targeted administration to the CNS.

The *SQSTM1* gene codes for p62, which is a scaffold protein which targets aggregating proteins for degradation via the autophagy/lysosomal pathway.^75^ It is involved in Parkin/PINK1-mediated mitophagy,^76^ as well as the toxicity associated with LRRK2.^77^

Of the biomarkers associated with metabolism, lower levels of PGK1 were associated with greater Parkinson’s disease severity. PGK1 is involved in neuronal glycolysis and ATP generation. Increased expression of PGK1 can preserve neuronal function in the presence of dopaminergic toxicity and has become an important potential target for disease-modifying intervention. Terazosin, a licensed drug for hypertension and bladder outflow obstruction, increases PGK1 activity and is entering trials as a potential Parkinson’s disease therapeutic.^78^

Of the biomarkers related to inflammation, CD40LG belongs to the TNF receptor superfamily and is a cytokine involved in the inflammatory response.^79^ Lower levels of CD40LG seen in our plasma and CSF samples may indicate greater binding of CD40LG to the CD40 receptor.

### Which serum proteins discriminate MSA from HC?

As expected, the NULISA assay confirmed NEFL as the serum protein that most distinguished MSA from HC in serum. In addition to NEFL, we also found elevated levels of pTau-181/MAPT and pTau-231/MAPT in MSA serum. Blood levels of both pTau-181^80^ and pTau-231^81^ have been confirmed to be useful predictors of Alzheimer’s disease diagnosis, reducing reliance on CSF biomarkers for the disease. We are not aware of any previous association between these phosphorylated tau proteins and MSA, although an increased level of CSF pTau/total tau ratio has been previously described in MSA compared with HC and Parkinson’s disease.^82^ While clinical misdiagnosis of MSA can occur in people who turn out to have pathologically confirmed PSP,^83^ we feel that misdiagnosis is unlikely to be the explanation for the tau associations we describe, given the rigorous trial inclusion criteria and extensive clinical assessments performed on all patients.

In contrast, the reductions in NRGN and ANXA5 levels in MSA serum compared with HC are opposite to our observation in Parkinson’s disease patients possibly reflecting the more aggressive disease process, or fundamental differences in the pathophysiological processes between the diseases. There were also reduced levels of PSEN1 (presenilin-1) in MSA patients, a protein well known in Alzheimer’s disease neurodegeneration with its involvement in the gamma secretase complex required for cleavage of APP, as well as having a role in synaptic vesicle replenishment. This is a novel observation, although a role for PSEN1 in alpha-synuclein expression in oligodendrocytes has previously been described.^84^

### Which proteins are associated with disease severity in MSA?

Despite being the strongest association distinguishing MSA from HC, neither serum nor CSF levels of NEFL were significantly associated with the severity of clinical scores in MSA patients. It has been shown previously that NEFL levels can predict the subsequent rate of clinical change in MSA patients, but the levels of serum or CSF NEFL can in fact decelerate later in the disease (presumably due to brain atrophy); hence, the strength of the relationship may differ if including longitudinal assessments.^85^ Our sample included individuals with established disease and our necessary adjustment for within-patient correlation across longitudinal assessments may explain the lack of any significant relationship being found.

A very strong association with disease severity was however seen in our analysis for the ratio of serum pSNCA-129/SNCA. pSNCA-129 in red blood cells has previously been proposed as a diagnostic biomarker for MSA,^86^ but to our knowledge, a relationship between serum levels of pSNCA-129/SNCA and MSA severity has not been previously reported; therefore, this is an important novel finding.

While IL-6 did not discriminate between Parkinson’s disease or MSA versus HC, we confirm an association between the levels of IL-6 and CRP in MSA serum and MSA severity, which demonstrates the relevance of systemic inflammation to this disease.^87^ We also identified a number of other neuroinflammatory chemokines to be associated with MSA-C severity. C–C motif chemokine 4 (CCL4) (previously known as macrophage inflammatory protein 1β) and CCL3 (previously known as macrophage inflammatory protein 1α), both act as chemoattractants to monocytes and macrophages at the site of damaged tissue. In addition, both these chemokines are secreted by activated microglia and bind to neuronal CCR5 receptors which ultimately disrupts neuronal autophagy and aggregate-prone protein clearance.^88^ Elevated levels of CCL4 and CCL3 have also been described in a transgenic mouse model of MSA.^89^

The ratio of pTau-181/MAPT and the level of CRH (corticotropin-releasing hormone) were the proteins most significantly associated with reduced MSA severity. While the ratio of serum pTau-181/MAPT is elevated in MSA serum compared to HC, lower levels of pTau181 in the serum with increased MSA severity are presumably a reflection of pTau181 aggregation in the brain. This intriguing relationship between the pathological forms of both pSNCA-129 and pTau-181 and MSA severity represents an important insight into MSA pathogenesis and is a potentially highly useful peripheral biomarker that may be relevant for clinical trial use.

CRH has previously been described to be reduced in the post-mortem brains (cerebellar hemispheres) of MSA patients.^90^ It is a peptidergic neurotransmitter produced by neurons in the inferior olivary complex, and its preservation may simply be a reflection of a less aggressive neurodegenerative process, and a relationship with disease severity has also been reported using the NULISA panel in dementia patients (Alzheimer’s disease, DLB, Frontotemporal dementia, PSP).^12^

### Can CSF biomarkers discriminate between MSA and Parkinson’s disease?

The most consistent discriminator between Parkinson’s disease and MSA was the CSF level of NEFL with almost 8-fold higher levels in MSA. NEFH, UCHL1, TREM1 and FCN2 were also 2-fold higher in MSA patients.

UCHL1 is an abundant, neuron-specific protein, comprising between 1 and 5% of total soluble brain protein. UCHL1 has multiple functions within the CNS and co-aggregates with alpha-synuclein within Lewy bodies which presumably accounts for its lower levels in the CSF of Parkinson’s disease patients.^91^ It plays a central role in the ubiquitin proteasome system (UPS). It has previously been found to have lower abundance in neurodegenerative diseases in CSF compared to HC, being particularly low in Parkinson’s disease compared with MSA potentially serving as a marker to distinguish these conditions.^92^

We identified that the inflammatory proteins CCL2, CCL3, CCL17, CCL22, CCL26 and CXCL8 were all significantly higher in CSF from MSA patients compared to Parkinson’s disease, potentially highlighting a greater role for modulation of the inflammatory response in MSA than Parkinson’s disease. We also found that both TREM1 and TREM2 levels were higher in the CSF of (particularly) MSA-C patients than Parkinson’s disease patients, contrasting with results from a previous study.^93^ Triggering receptor expressed on myeloid cell 1 (TREM1) is an important regulator of cellular inflammatory responses. In keeping with this, previous research has linked TREM1 to Parkinson’s disease pathogenesis,^94^ and reducing levels of TREM1 has been shown to be helpful in rat and zebrafish^95^and mouse models of Parkinson’s disease.^96^

### Limitations

The NULISA panel has been designed to be of use in the assessment of CNS protein changes across a number of neurodegenerative and non-neurodegenerative diseases. While the ultimate goal of any proteomic panel is to identify changes in proteins which have the greatest potential to serve as diagnostic, prognostic and disease tracking markers, it is important to highlight that altered protein levels in disease compared to healthy controls may or may not be directly implicated in the disease pathophysiology. Large-scale untargeted datasets may capture both secondary/bystander consequences of disease, as well as direct pathogenic drivers and host compensatory mechanisms, and disentangling these is challenging. They are also often confounded by the intrinsic heterogeneity of the disease pathophysiology as well as interindividual genetic and/or variability in relation to comorbidity or drug exposures.

Despite the wealth of important and intriguing results included in this study, we acknowledge that there are a number of limitations. We have confirmed previous reports from Alzheimer’s disease samples that the NULISA platform allows accurate multiplex measurements using low sample volumes, which can integrate measurements from several study visits using either peripheral blood-derived samples or CSF. Our comparison of results from paired plasma versus serum in the same HC individuals suggests both matrices are acceptable for testing using the platform, but different NPQ values are detected in different matrices even when samples are collected from the same individuals contemporaneously and therefore prevented any meaningful comparison between our Parkinson’s disease plasma and MSA serum samples. Additionally, we did not have access to age-matched HC CSF samples and therefore our CSF comparisons are restricted to Parkinson’s disease versus MSA and in association with longitudinal disease severity.

While the sample size is large for a biomarker study, we included a larger number of Parkinson’s disease participants than MSA participants; therefore, some significant associations seen in the Parkinson’s disease cohort that were not found in the MSA cohort might simply be due to the smaller MSA sample size. While a strength of our data is that we have longitudinal clinical information regarding disease severity, the subjects were exposed to a range of dopaminergic and other symptomatic medications which may have had unknown influences on protein expression. Given that these samples were taken from clinical trials of exenatide, appropriate adjustment was made for exposure to this drug in the severity analyses that included samples taken post-exenatide exposure.

In our assessment of disease severity, we use the MDS UPDRS Part 3 score to measure Parkinson’s disease severity and the UMSARS Part 1 + 2 score to measure MSA severity. Both of these diseases can also lead to substantial non-motor symptom burden, and therefore the use of these scales may not fully capture the degree of disease progression in all individuals. Exploring the relationship between protein levels and non-motor symptoms such as cognition will be performed separately.

## Conclusions

We have reported the results of a large number of analyses made possible by the use of the NULISA CNS disease panel providing quantification of CNS proteins detected in peripheral and CSF samples collected longitudinally in Parkinson’s disease patients, MSA patients and HC. Importantly combining the breadth of the CNS disease panel, together with the relevance of the panel to disease-specific mechanisms in neurodegeneration and synucleinopathies, we were able to identify key classes of disease-relevant proteins that are associated with Parkinson’s disease and MSA. Together with prior knowledge of the role of these proteins in triggering or driving pathology in Parkinson’s disease, we can hypothesize whether these biomarkers reflect causal or secondary processes. Key to our study has been the ability to find markers which are highly significantly associated with disease severity and therefore have utility in disease-modifying trials as quantifiable and sensitive measures of progression.

We highlight three key findings:

- Levels of ‘multiple’ aggregating proteins differ between Parkinson’s disease and MSA from controls in peripheral biofluids. In particular, oligomeric alpha-synuclein can be directly detected and quantified using the NULISA panel; however, the presence of other pathological forms of aggregating proteins, specifically, plasma levels of TDP43 and HTT, is also elevated in association with Parkinson’s disease, while pTau-181/MAPT ratios are elevated in association with MSA-P. While the ratios of abnormal forms of alpha-synuclein to the total levels are clearly strongly related to disease state and severity, our view of these clinical phenotypes as being single proteinopathies is likely oversimplistic.
- Biomarkers of neuronal loss, such as NEFL, reliably distinguish the rapidly neurodegenerative synucleinopathy MSA from HC; however, with advancing disease severity, this relationship is not linear, and other markers of disease activity such as IL-6 may be more appropriate measures. The aggressive and rapid neurodegeneration associated with MSA is likely driven by both protein aggregation and neuroinflammation, and measurements of IL6 may be specifically useful in an interventional trial, especially of an agent targeting neuroinflammation.
- Peripheral biofluids clearly have great potential as disease markers, e.g. biomarkers of metabolism such as TAFA5 peripherally. These results need to be further replicated; however, they may provide further insights into the relationships between metabolism and neurodegeneration. CSF markers also reveal additional insights, particularly IGF1R, which changes with disease severity in Parkinson’s disease and may reflect a shift in bioenergetic status and insulin signalling in the brain and could therefore be used for disease-modifying therapies that target mitochondrial metabolism or insulin resistance.

## Supplementary Material

fcag035_Supplementary_Data

## Data Availability

Data analysed for this manuscript will be made available for sharing on reasonable request to T.F.
