# Supplementary material for: Peripheral and CSF protein quantification in Parkinson’s disease and multiple system atrophy—the nucleic acid-linked immuno-sandwich assay
Source: Brain Commun. 2026 Feb 4;8(2):fcag035. doi: 10.1093/braincomms/fcag035 (PMC12954390; doi:10.1093/braincomms/fcag035)

**Supplementary material**

**Supplementary Table 1. NULISAseq CNS disease Panel 120**


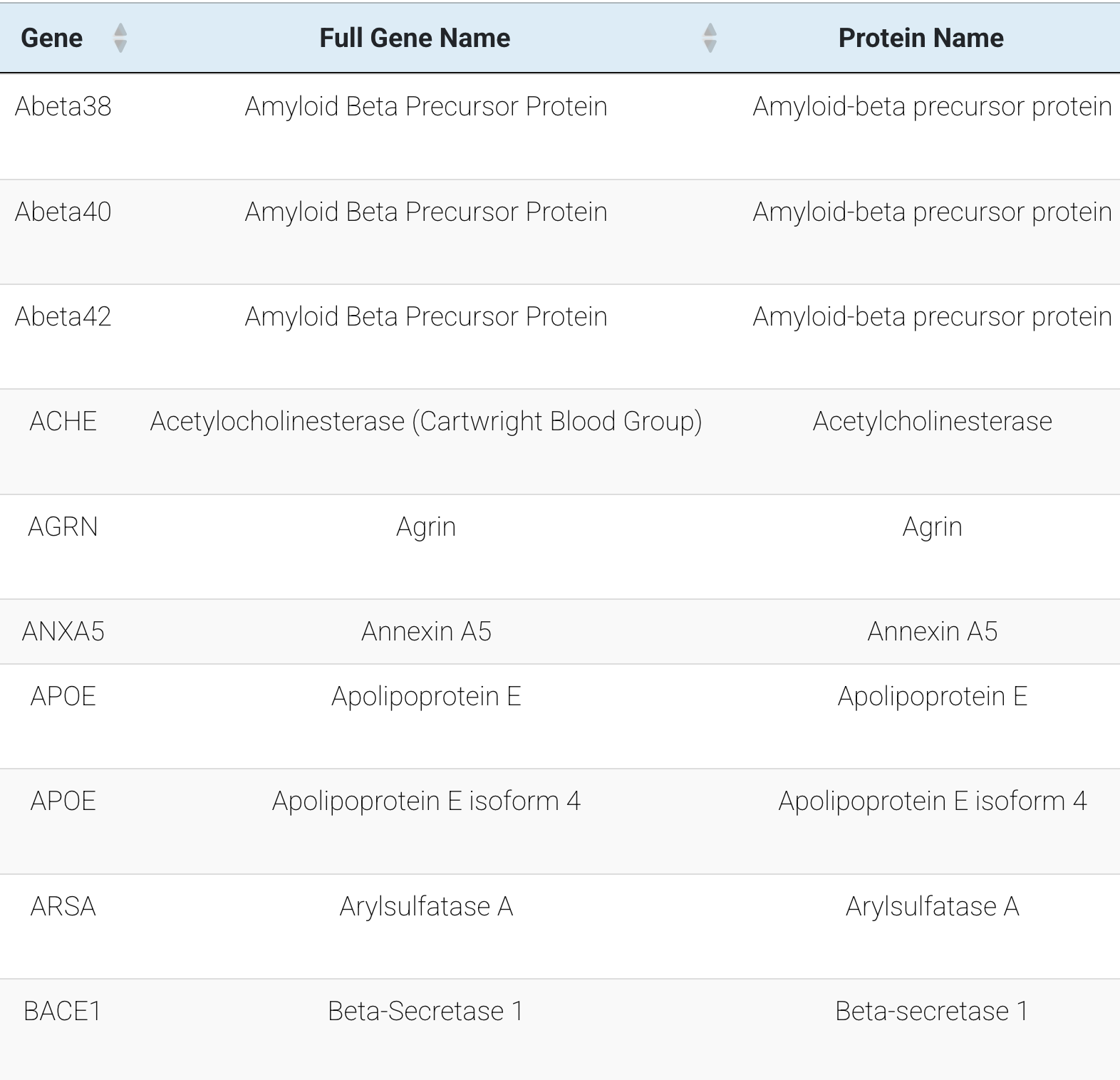


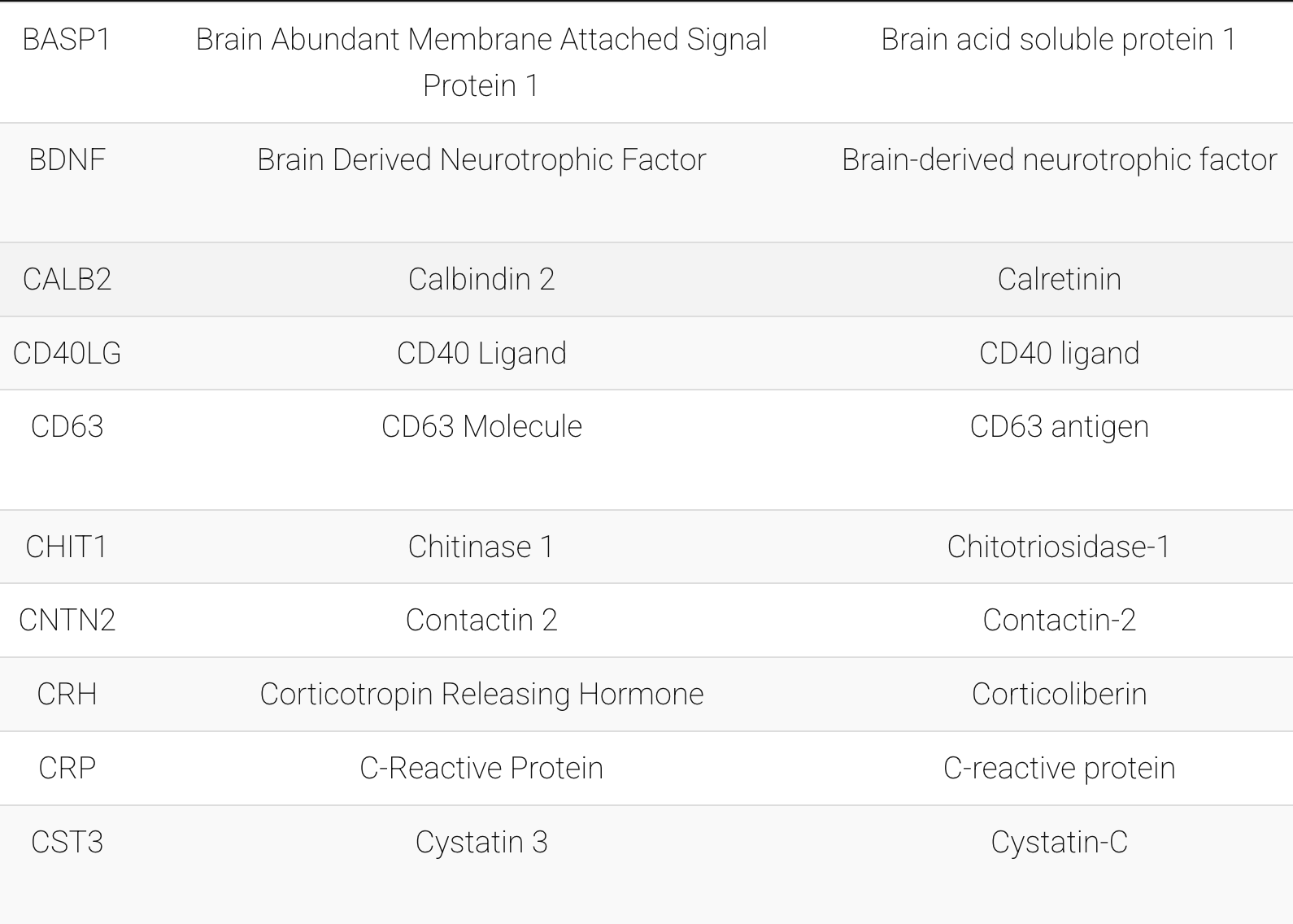


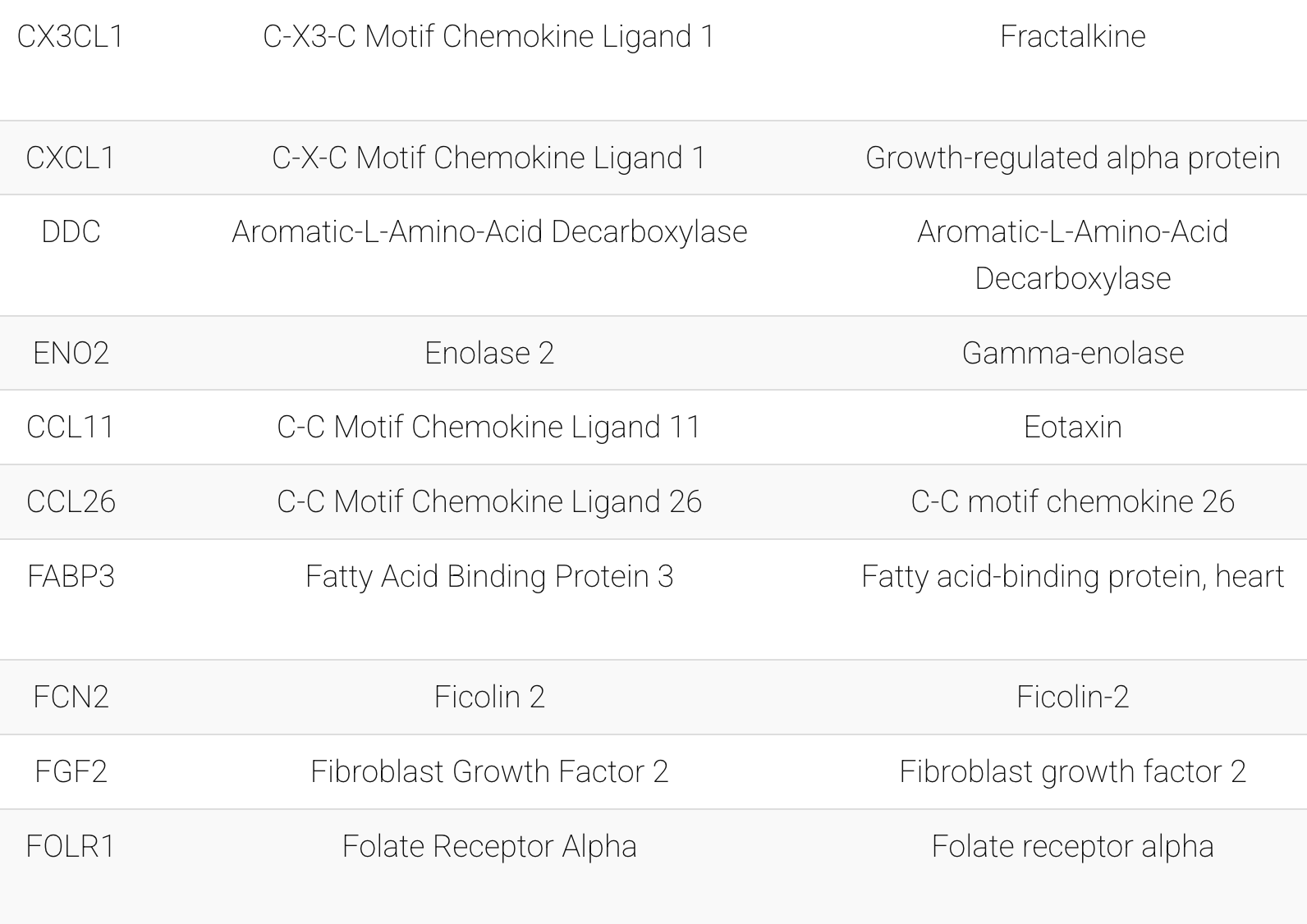


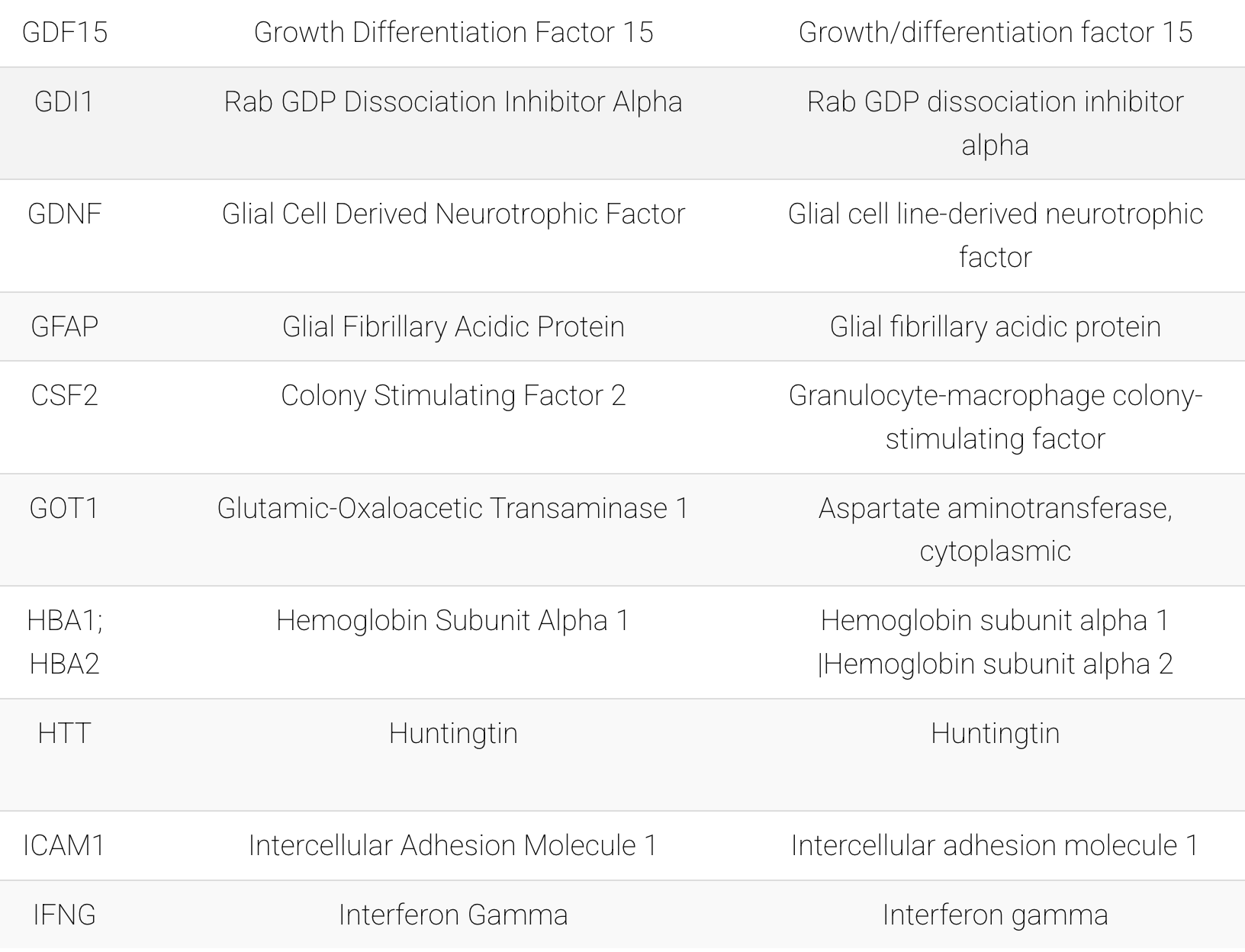


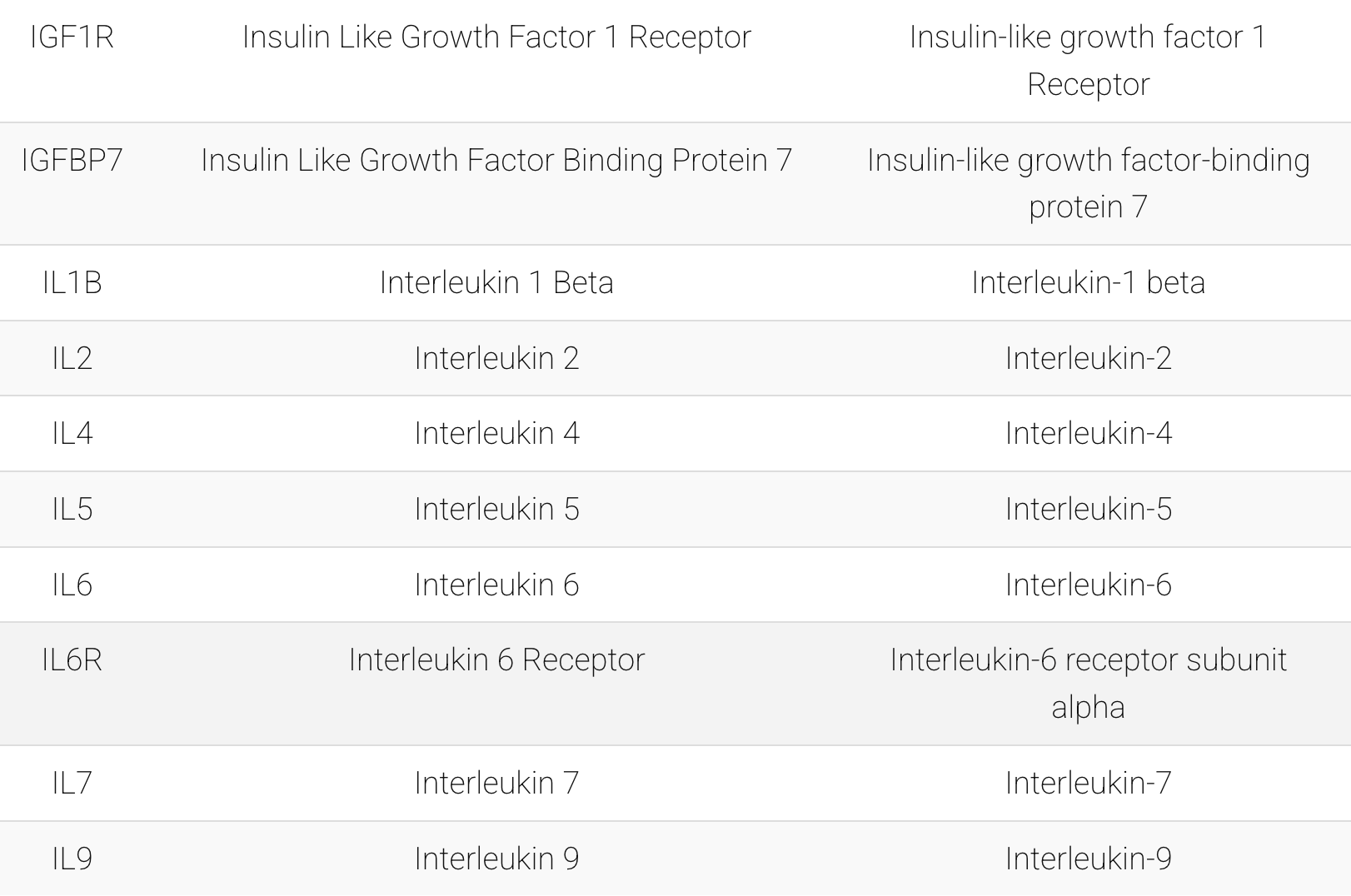


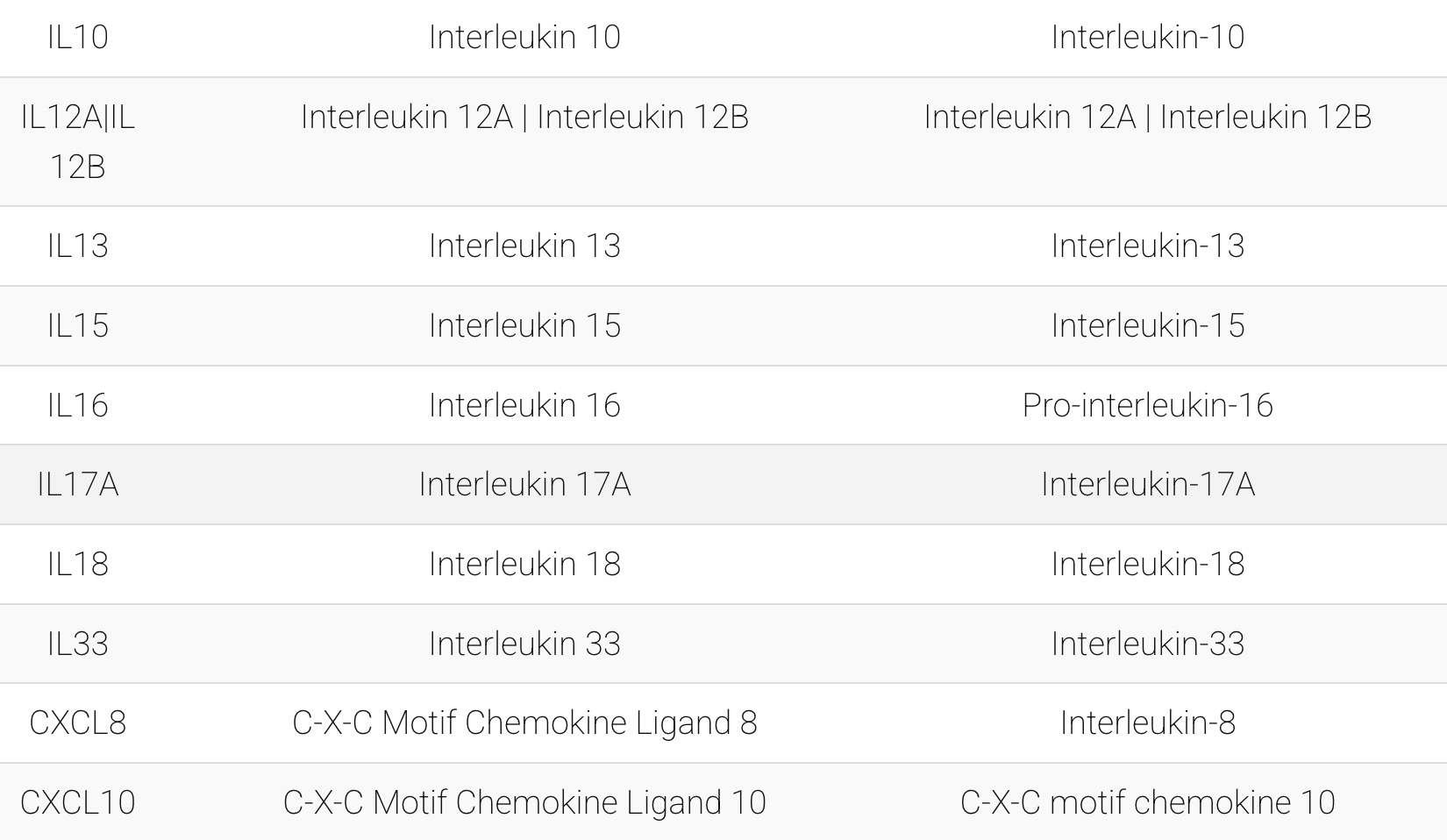


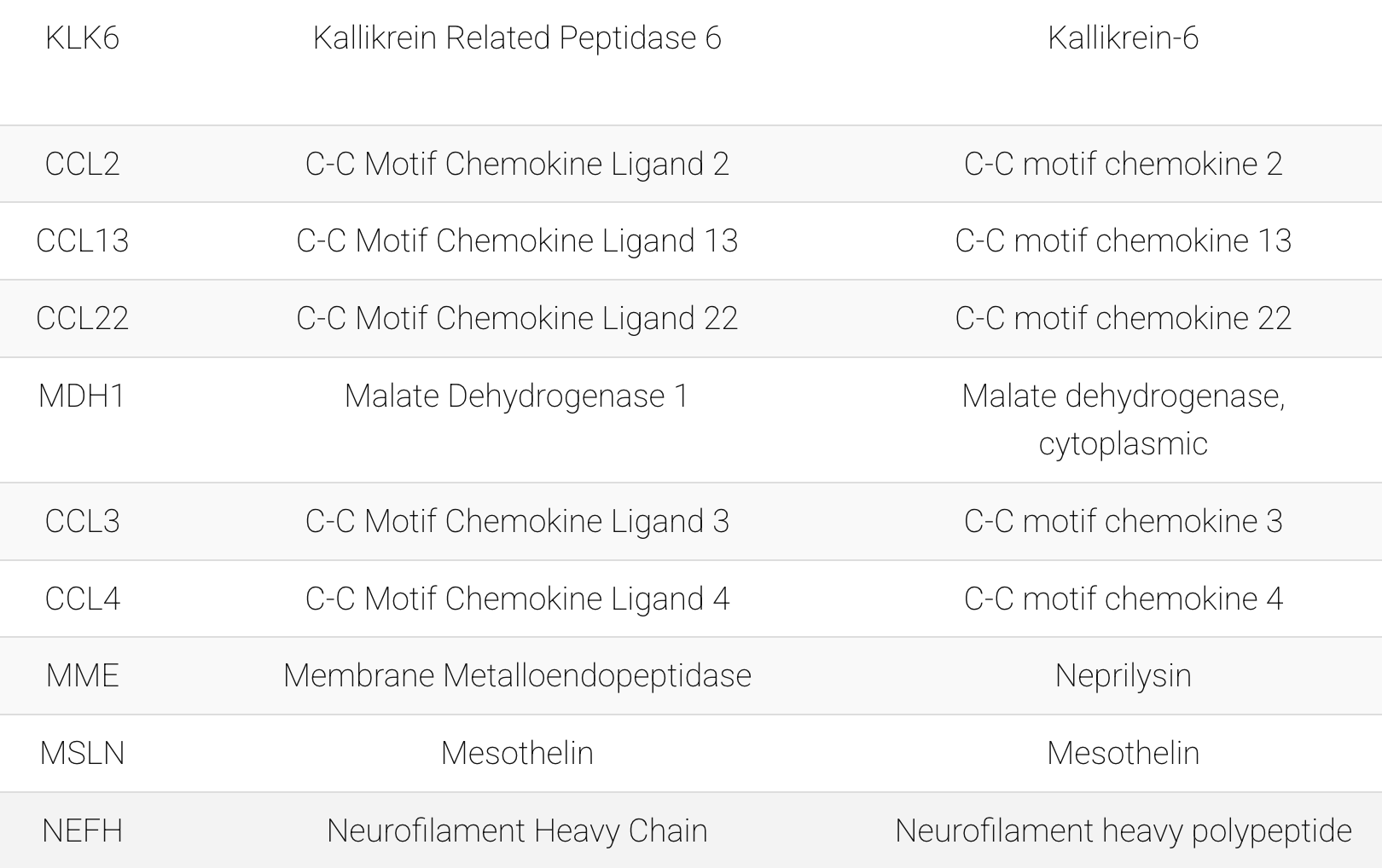


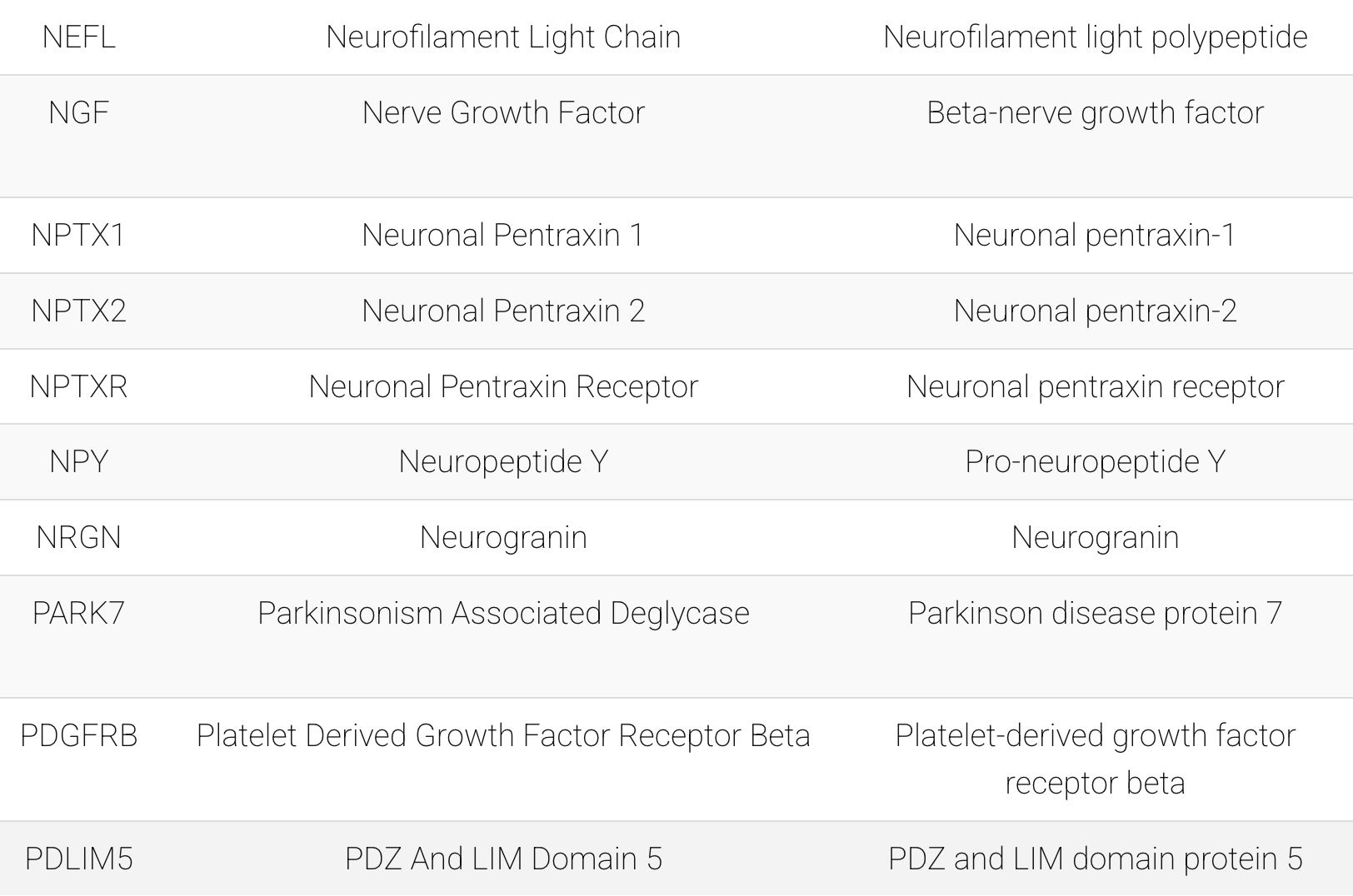


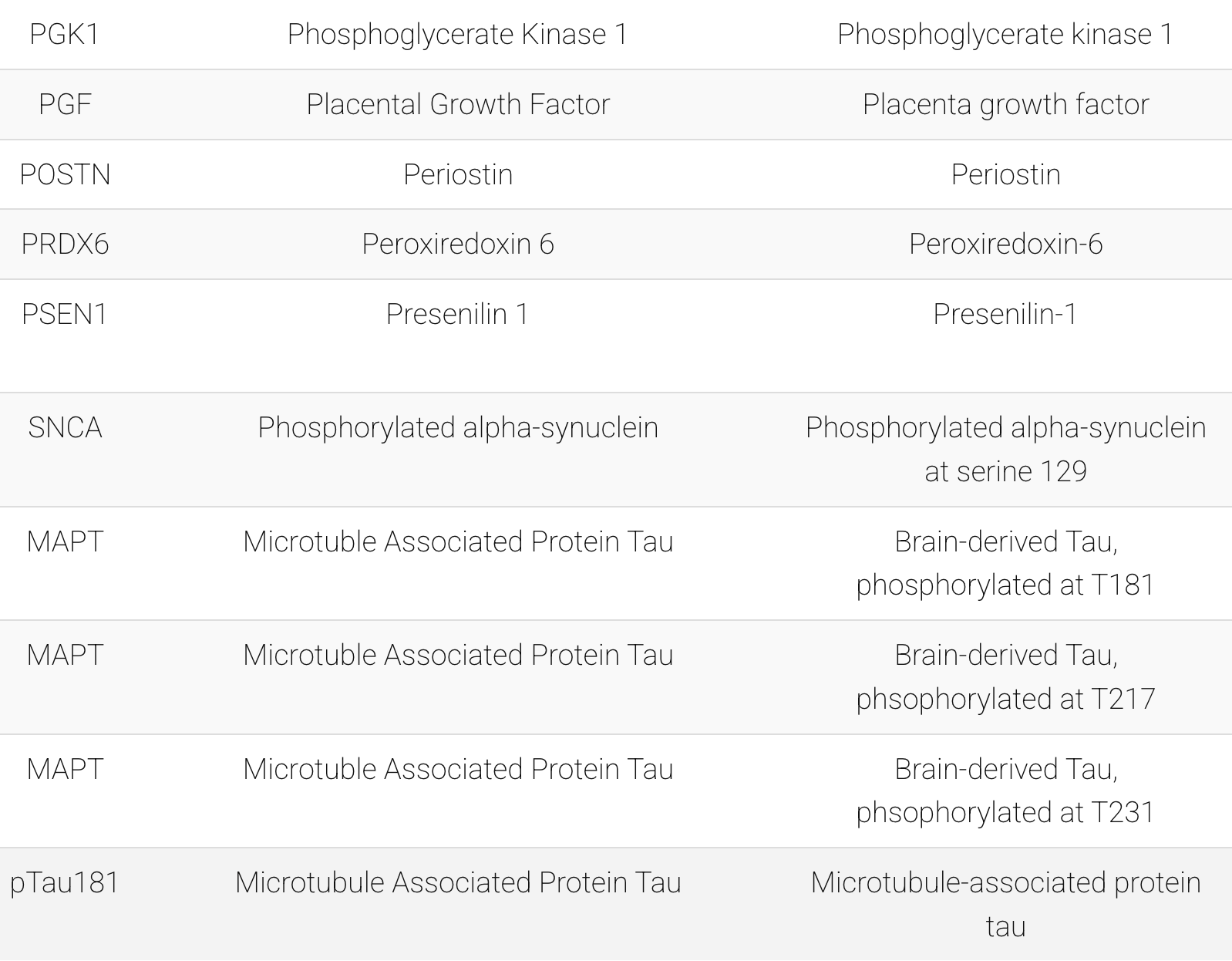


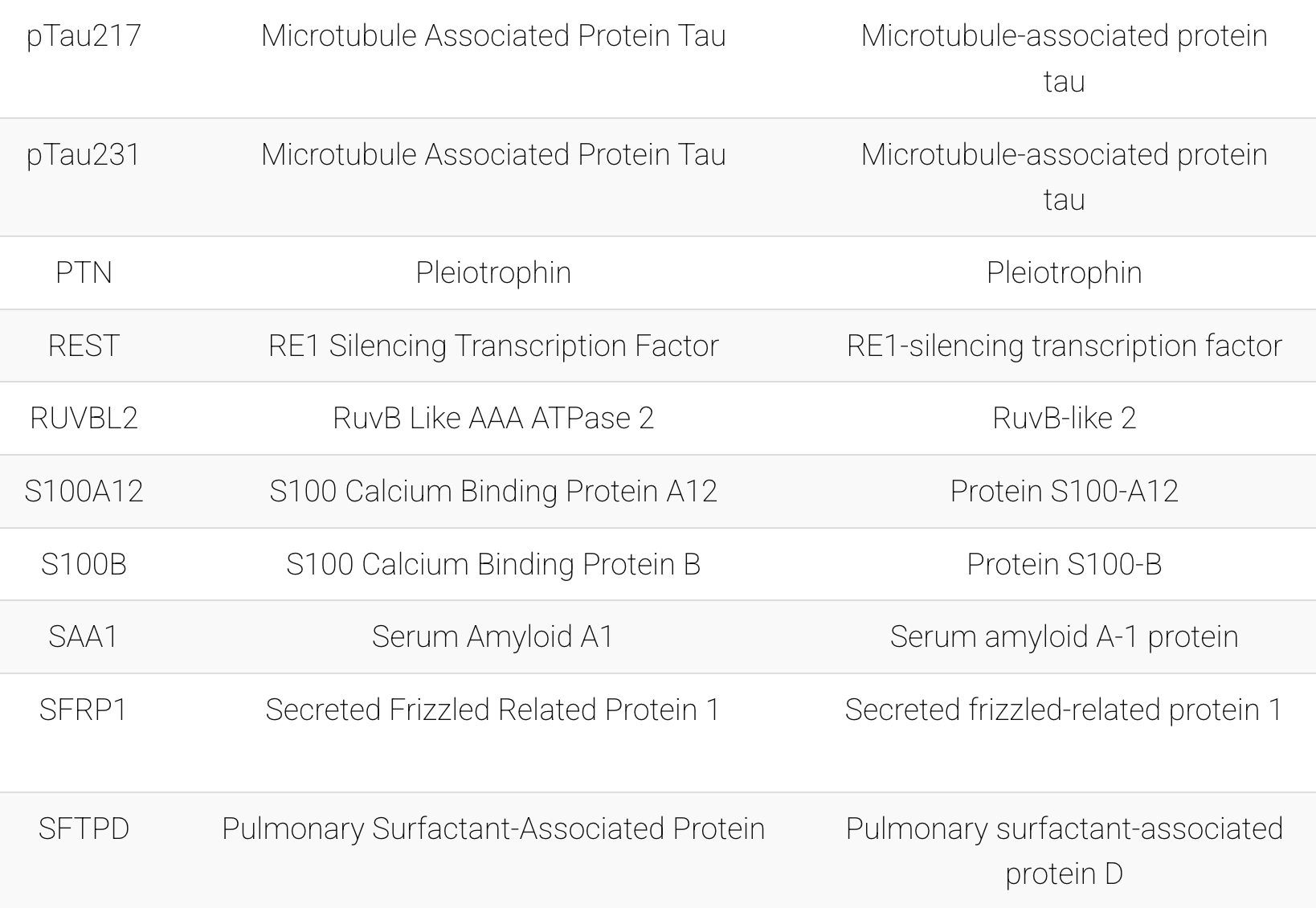


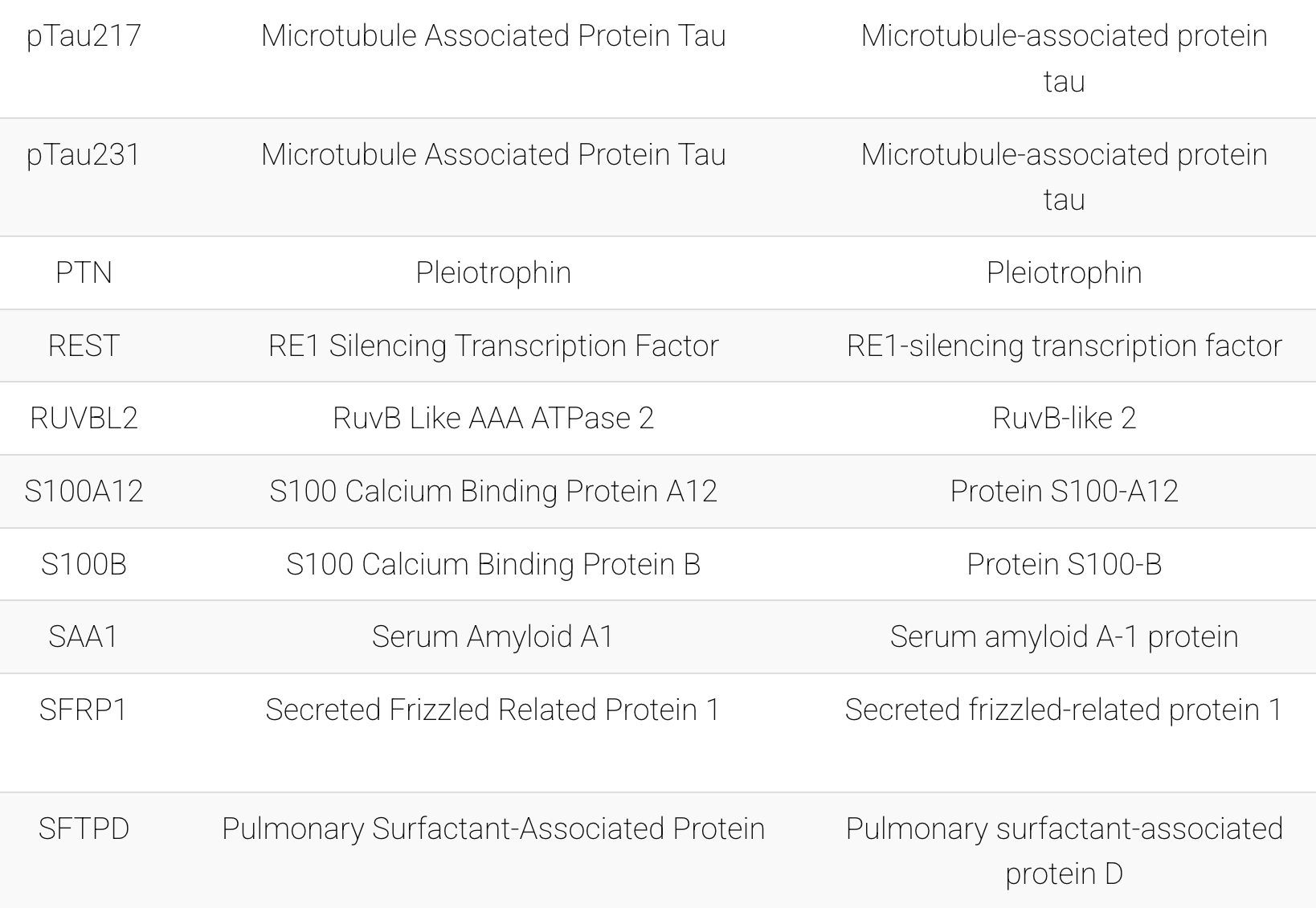


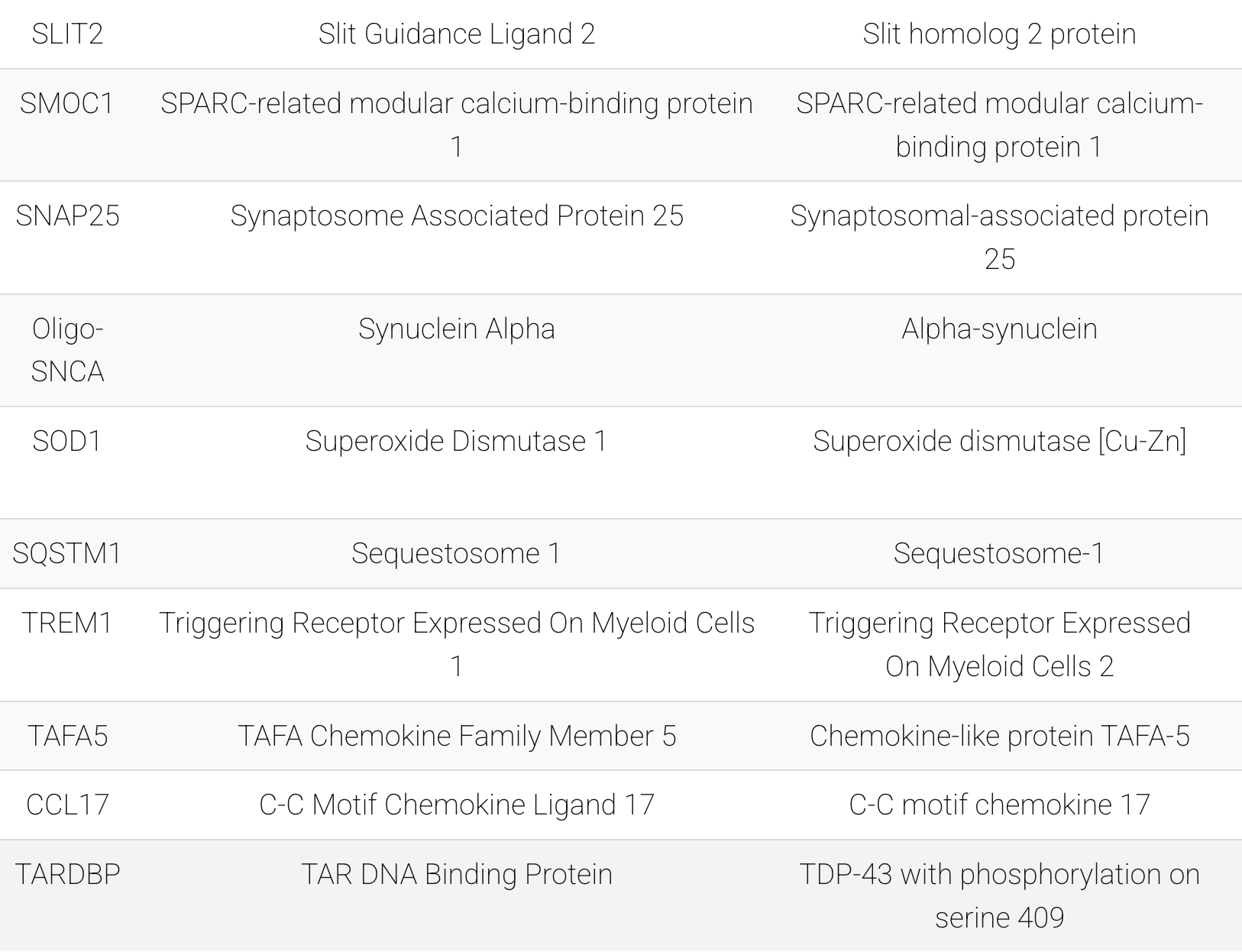


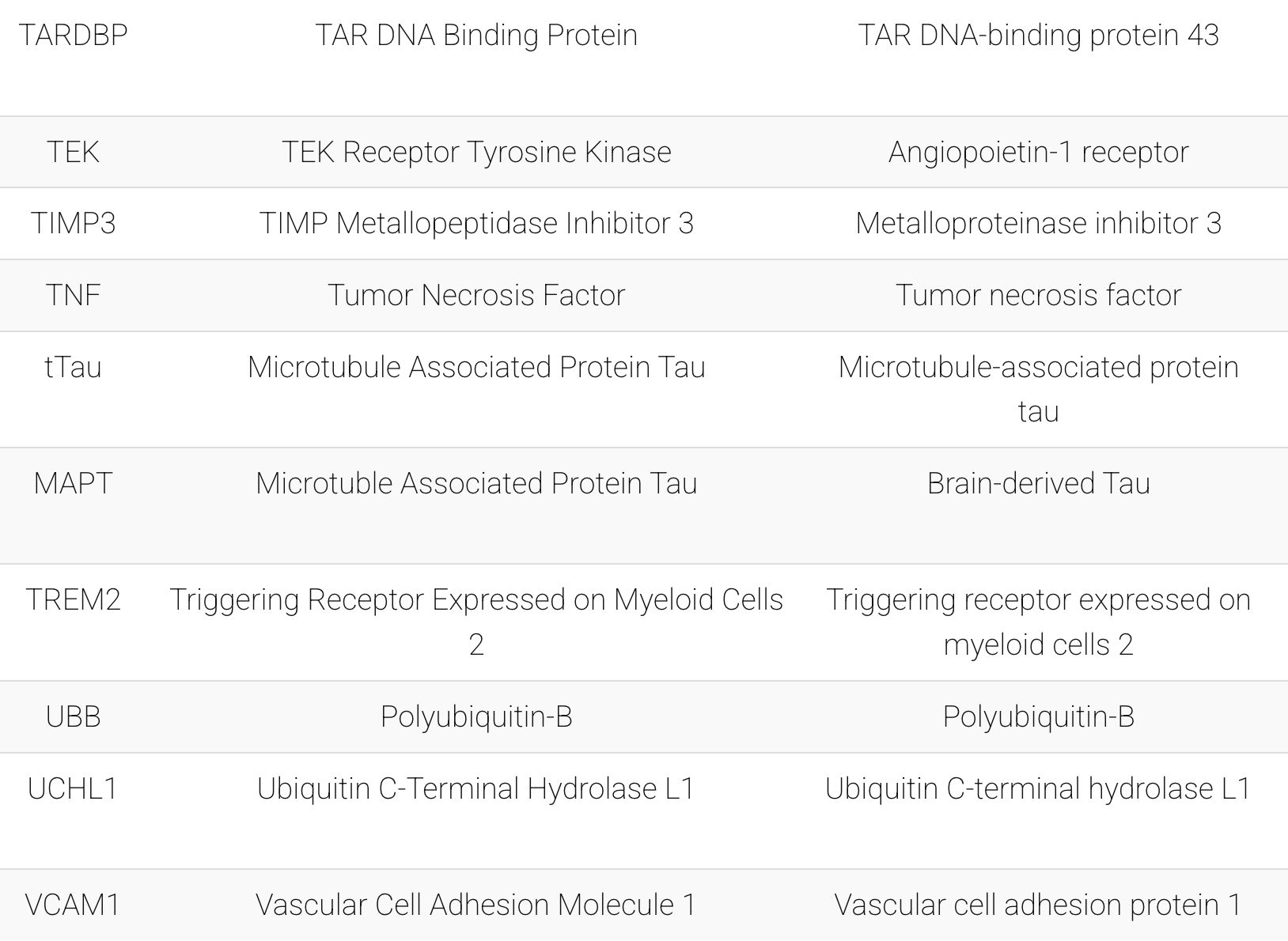


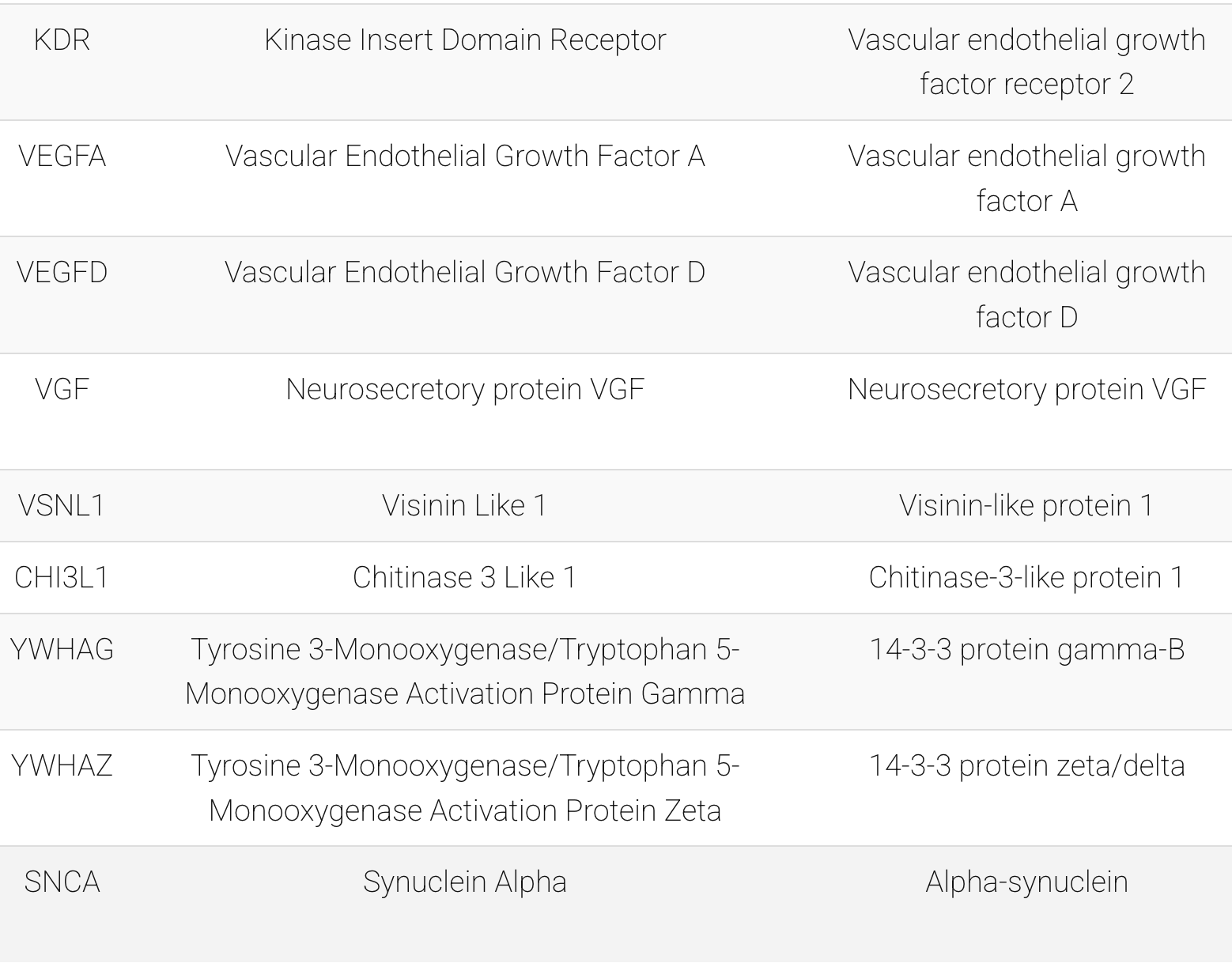


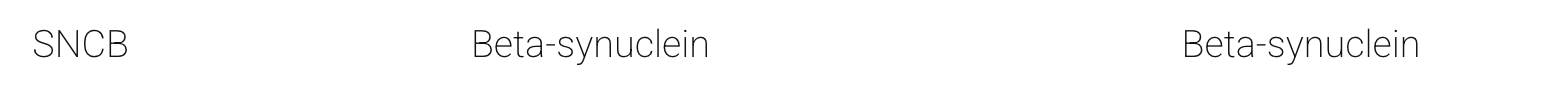


**Supplementary Table 2. Summary of poorly detectable targets**

| **Sample matrix** | **Targets excluded (<50% overall detectability)** |
| --- | --- |
| **Plasma** | APOE4, GDI1, PTN, SNCB, UCHL1, YWHAZ |
| **Serum** | APOE4, GDI1, IL1B, PTN, SNCB, UCHL1, YWHAZ, pTDP43-409 |
| **CSF** | ARSA, BASP1, BDNF, CDNF, HBA1, IFNG, IL17A, IL1B, IL2, IL33, IL4, MME, Oligo-SNCA, PARK7, PDLIM5, pTDP43-409, S100A12, SAA1, VEGFD, VGF, YWHAZ |

Supplementary Figure 1. PD plasma samples (n=161) taken at baseline trial visits compared to Healthy Control plasma samples (n=39) using a categorical linear model adjusting for age at baseline and sex. Boxplots showing upregulated relative protein NPQ values in PD vs HC plasma samples. Boxplots show median, interquartile range, and whiskers show minimum and maximum values. Additional dots show outlying values.


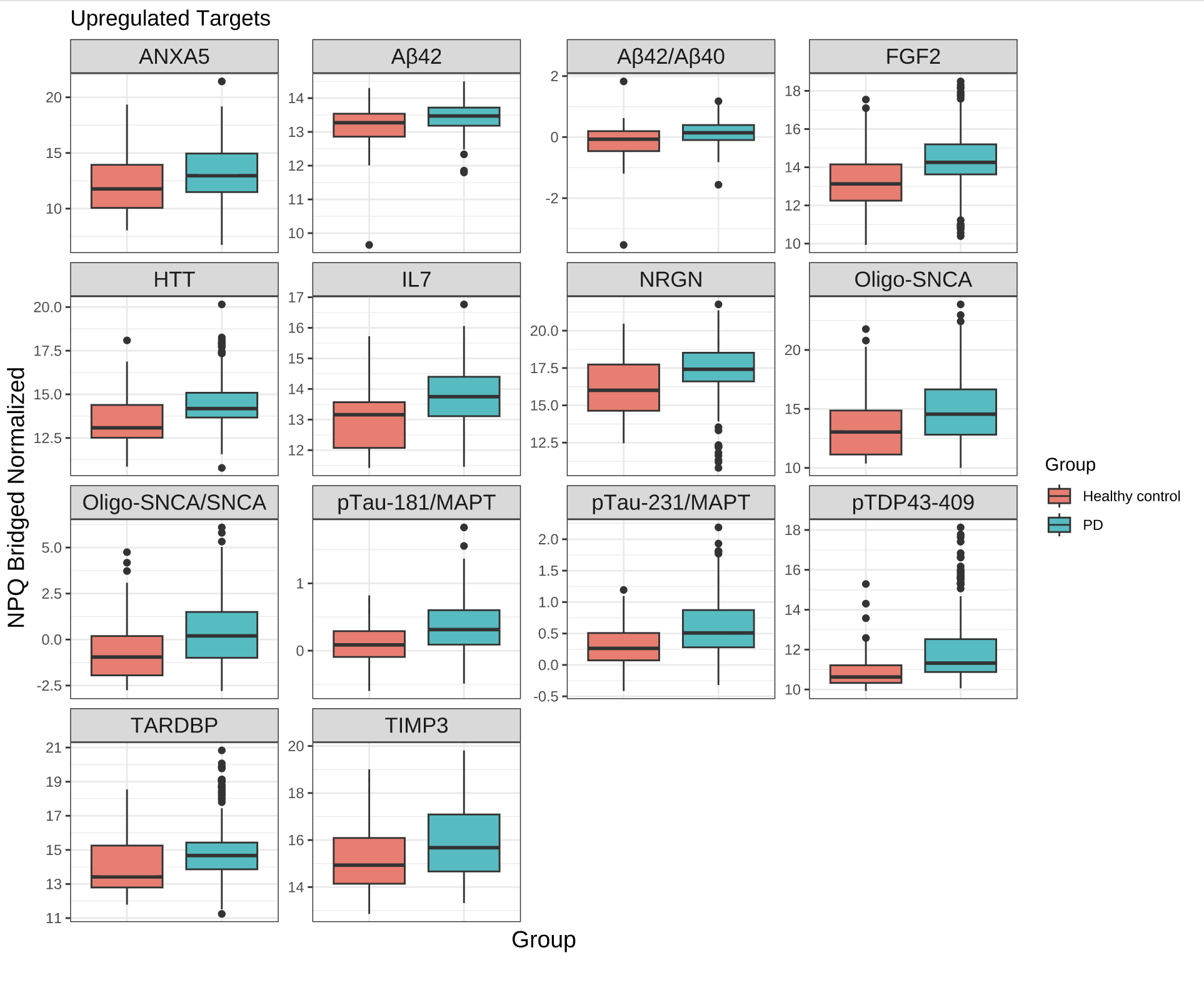


Supplementary Figure 2. PD plasma samples (n=161) taken at baseline trial visits compared to Healthy Control plasma samples (n=39) using a categorical linear model adjusting for age at baseline and sex. Boxplots showing downregulated relative protein NPQ values in PD vs HC plasma samples. Boxplots show median, interquartile range, and whiskers show minimum and maximum values. Additional dots show outlying values.


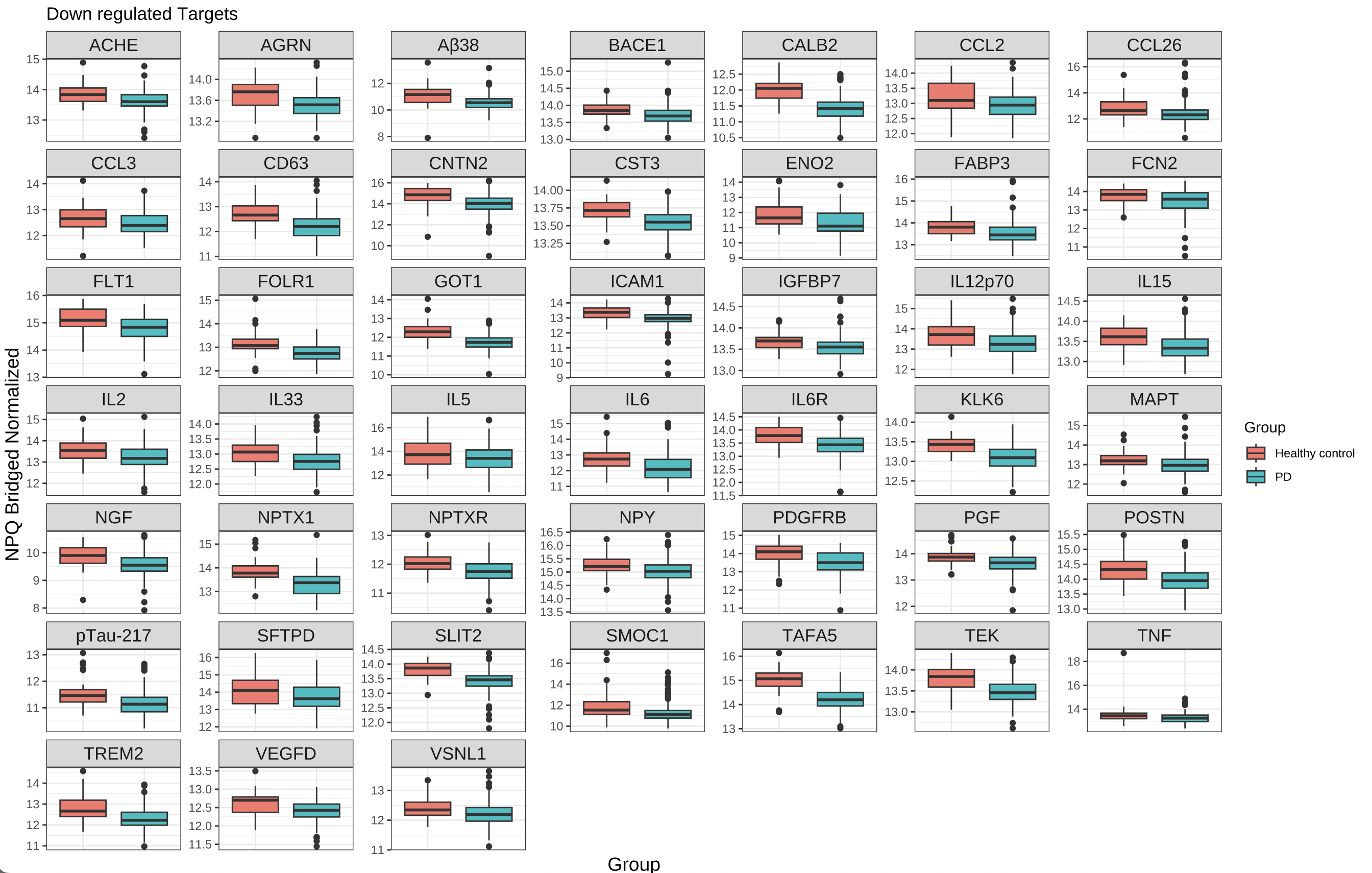


Supplementary Figure 3. PD Plasma samples (n=161) collected at multiple timepoints and compared to PD severity (MDS UPDRS part 3) using linear mixed-effects regression modelling, adjusting for age at baseline, sex, and exenatide drug randomisation, and a random effect for subject to account for within-subject correlation. Spaghetti plots showing individual variations in PD plasma NPQ values at different time points on x axis against disease severity score (MDS UPDRS part 3) on y axis.


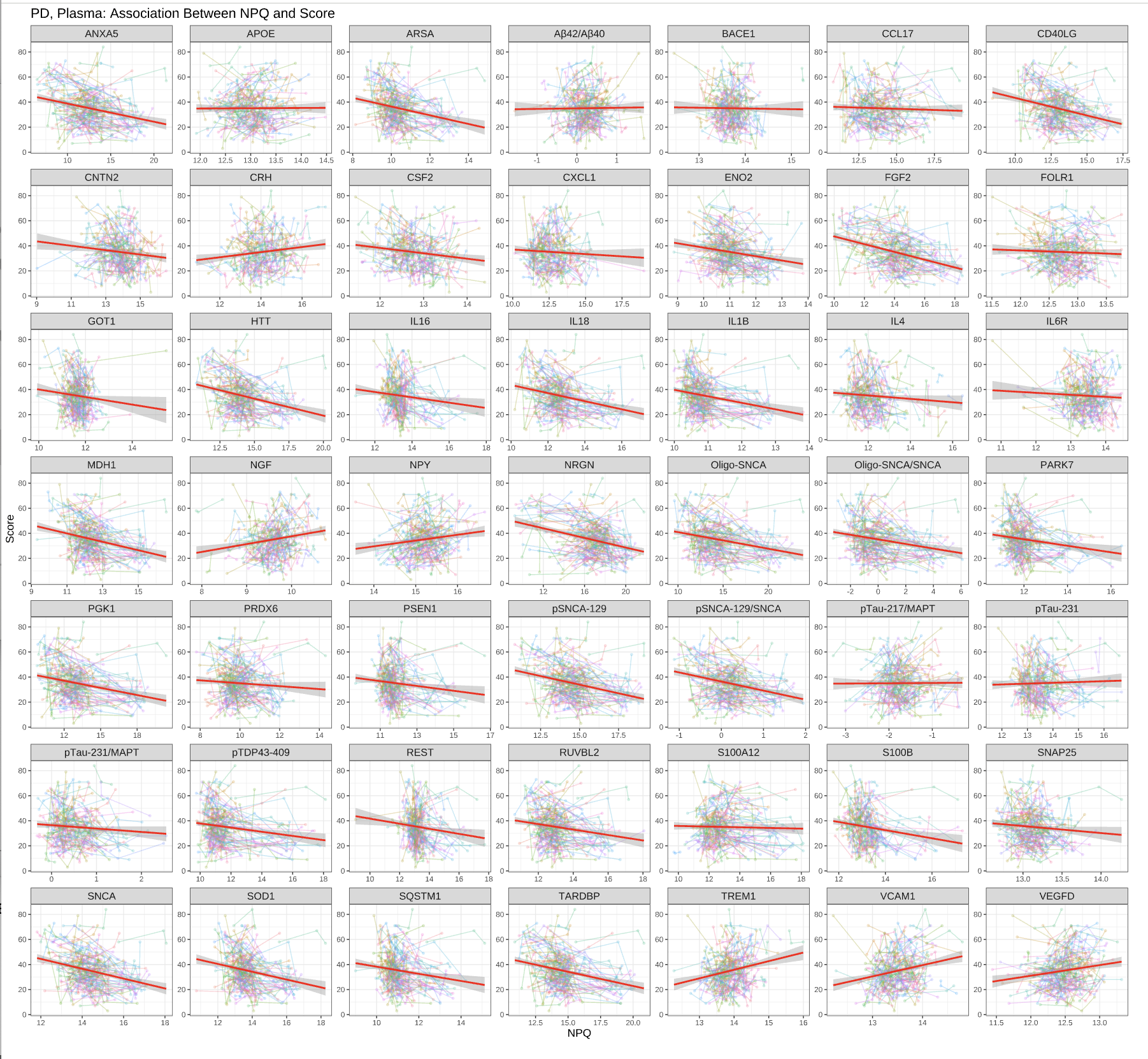


Supplementary Figure 4. PD CSF samples (n=51) collected at multiple timepoints and compared to PD severity (MDS UPDRS part 3) using linear mixed-effects regression modelling, adjusting for age at baseline, sex, and exenatide drug randomisation, and a random effect for subject to account for within-subject correlation. Spaghetti plots showing individual variations in PD CSF NPQ values at different time points on x axis against disease severity score (MDS UPDRS part 3) on y axis.


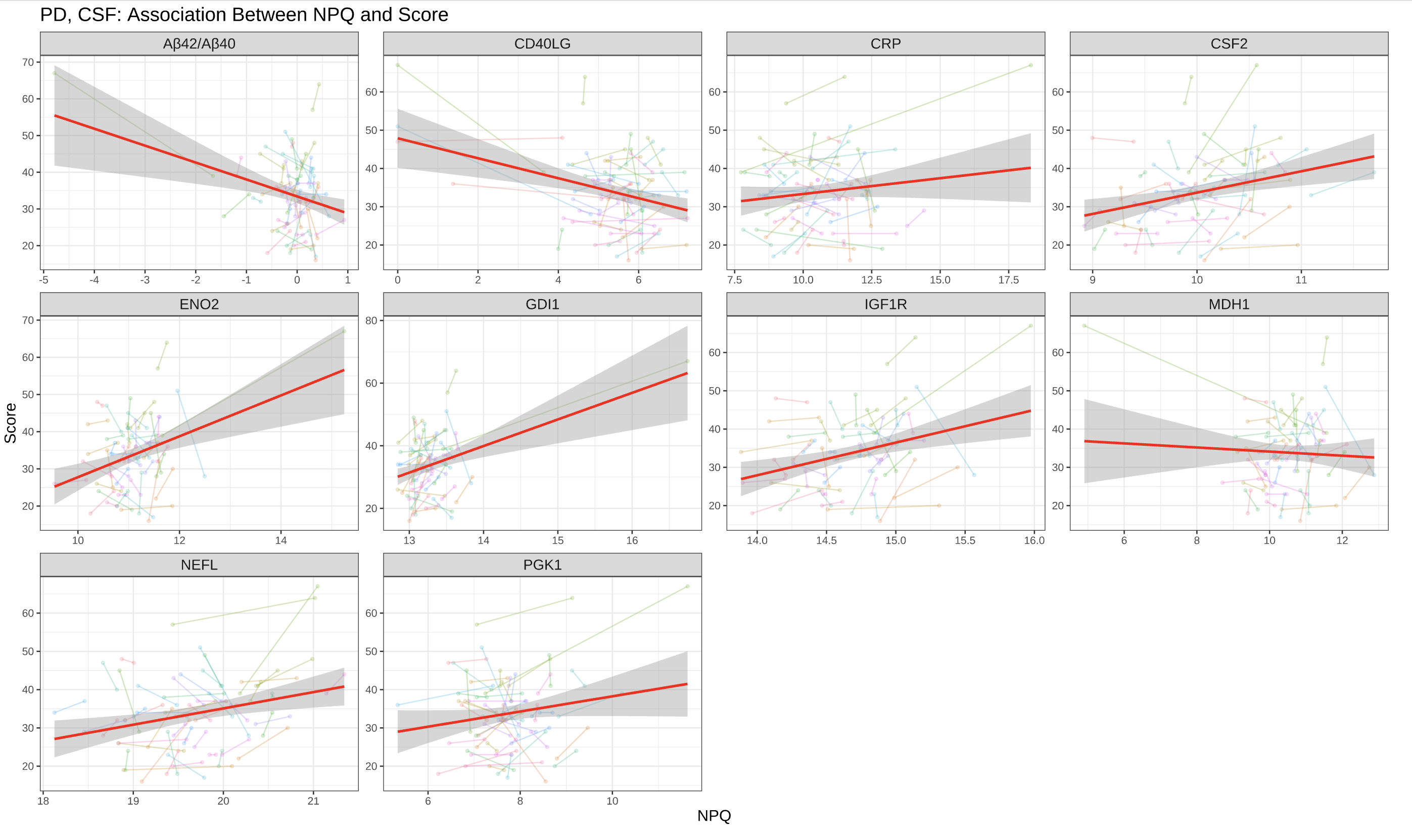


Supplementary Figure 5. MSA serum samples (n=42) taken at baseline trial visits compared to Healthy Control serum samples (n=39) using a categorical linear model adjusting for age at baseline and sex. Boxplots showing relative upregulated protein NPQ values in MSA serum samples. Boxplots show median, interquartile range, and whiskers show minimum and maximum values. Additional dots show outlying values.


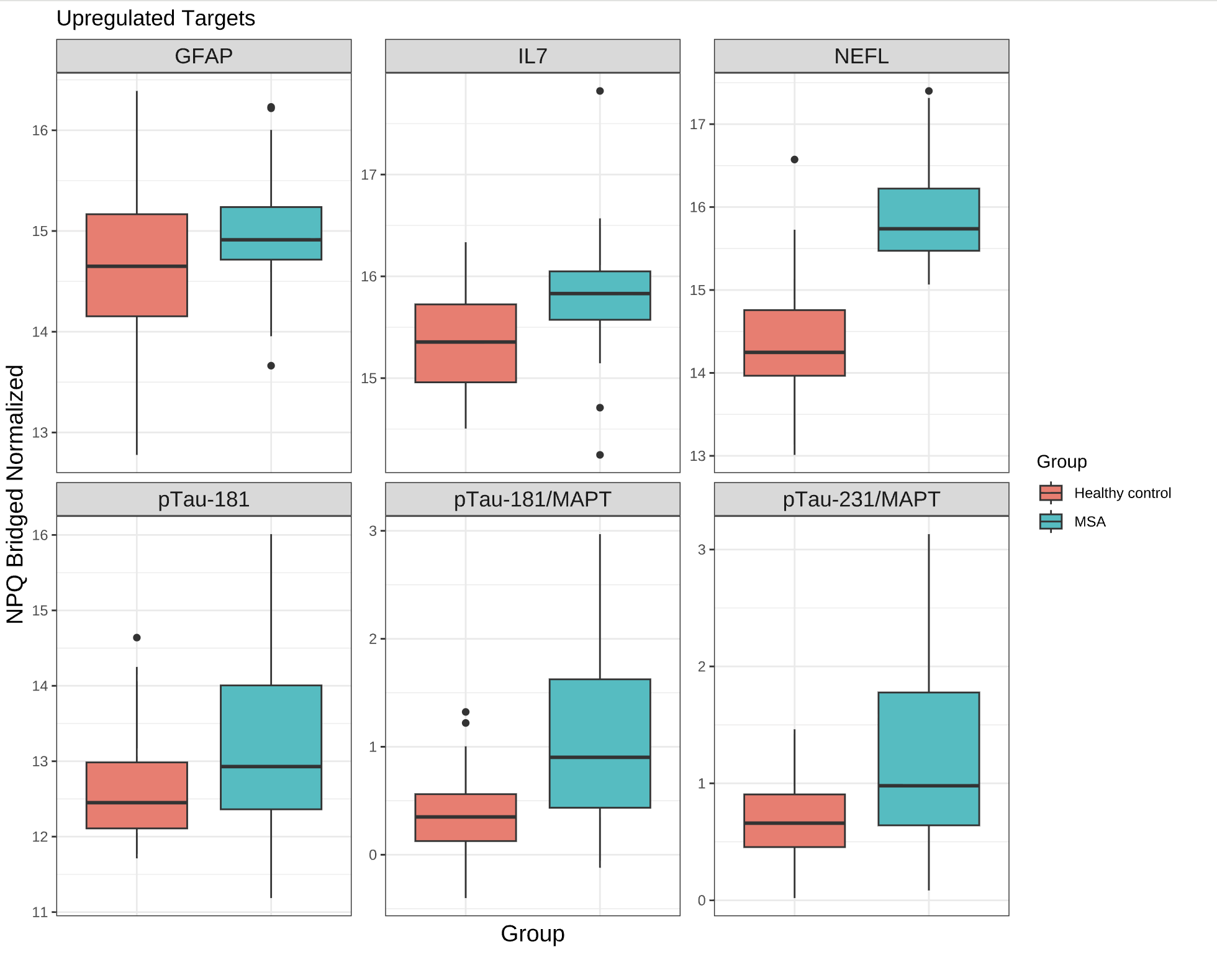


Supplementary Figure 6. MSA serum samples (n=42) taken at baseline trial visits compared to Healthy Control serum samples (n=39) using a categorical linear model adjusting for age at baseline and sex. Boxplots showing relative downregulated protein NPQ values in MSA samples. Boxplots show median, interquartile range, and whiskers show minimum and maximum values. Additional dots show outlying values.


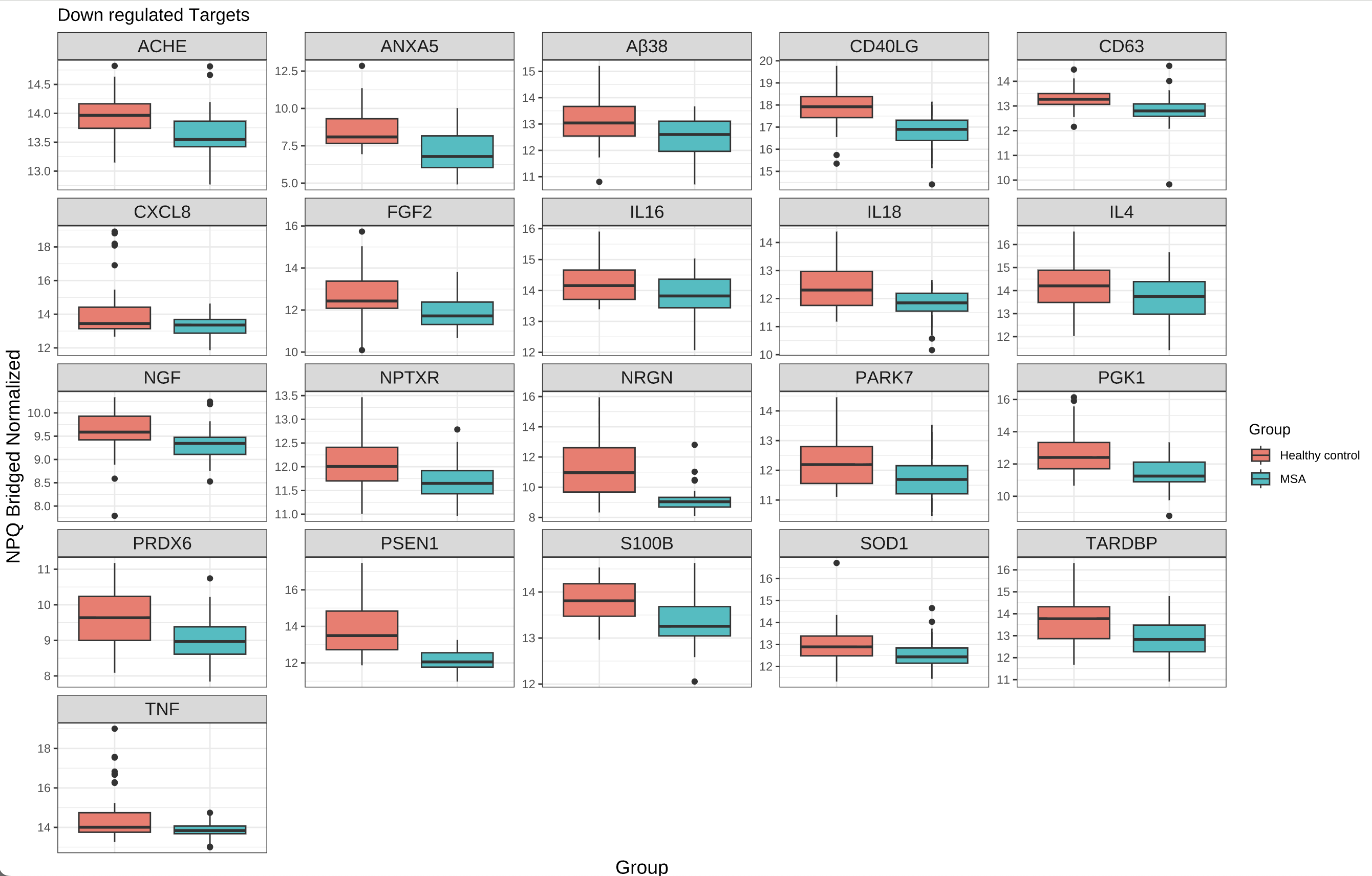


Supplementary Figure 7. MSA-P serum samples (n=22) taken at baseline trial visits compared to Healthy Control serum samples (n=39) using a categorical linear model adjusting for age at baseline and sex. The volcano plot shows Log_10_ adjusted FDR adjusted p values represented on the y axis i.e. a Log_10_ adjusted FDR adjusted p value of 5 represents p=0.00001. The x axis shows the log_2_ fold change, i.e. positive fold changes (proteins displayed in red) of +1 indicate that the protein values are twice as high in the MSA-P samples compared with control samples.


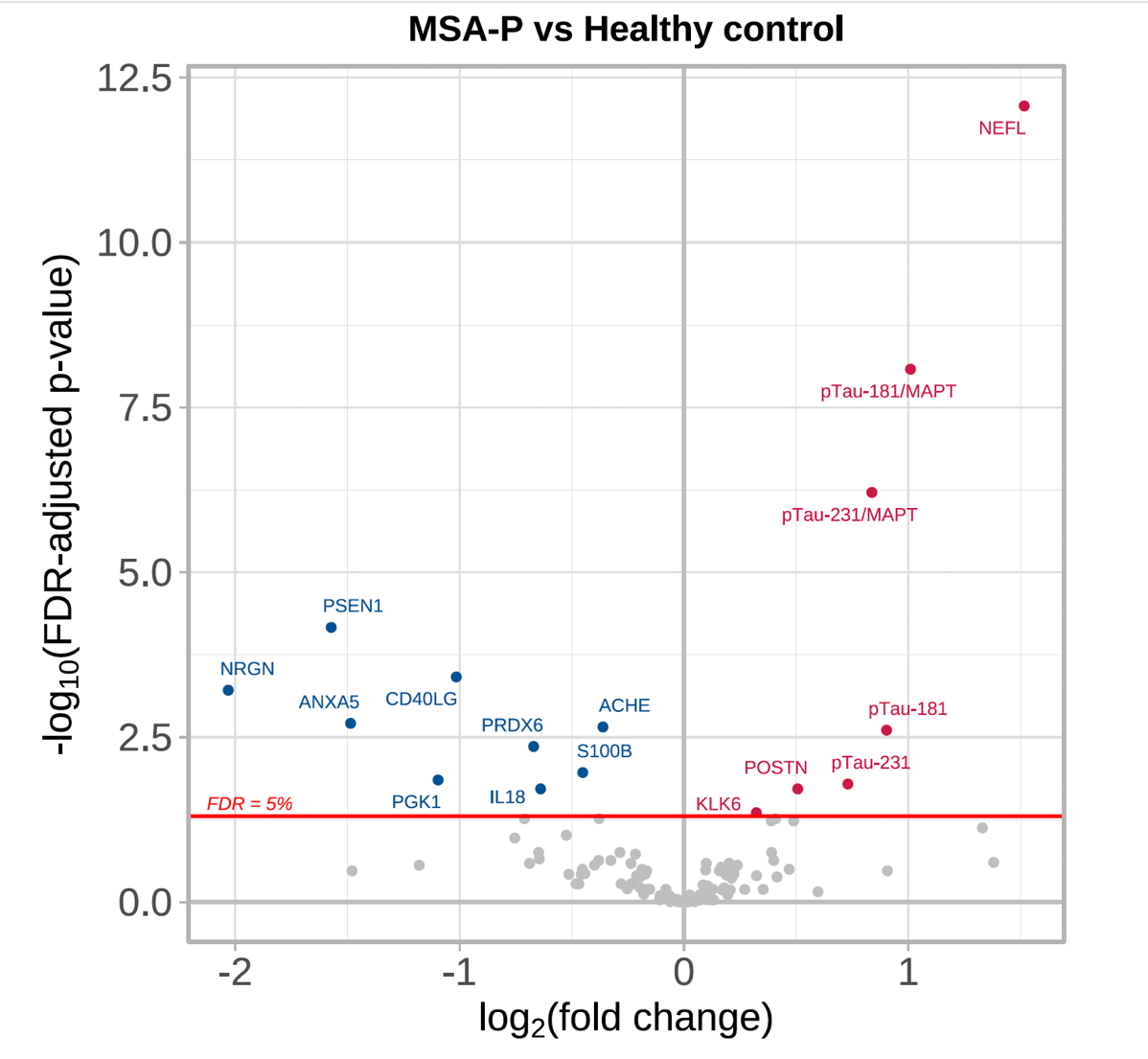


Supplementary Figure 8. MSA-P serum samples (n=22) taken at baseline trial visits compared to Healthy Control serum samples (n=39) using a categorical linear model adjusting for age at baseline and sex. Boxplots showing relative upregulated protein NPQ values in MSA-P serum samples. Boxplots show median, interquartile range, and whiskers show minimum and maximum values. Additional dots show outlying values.


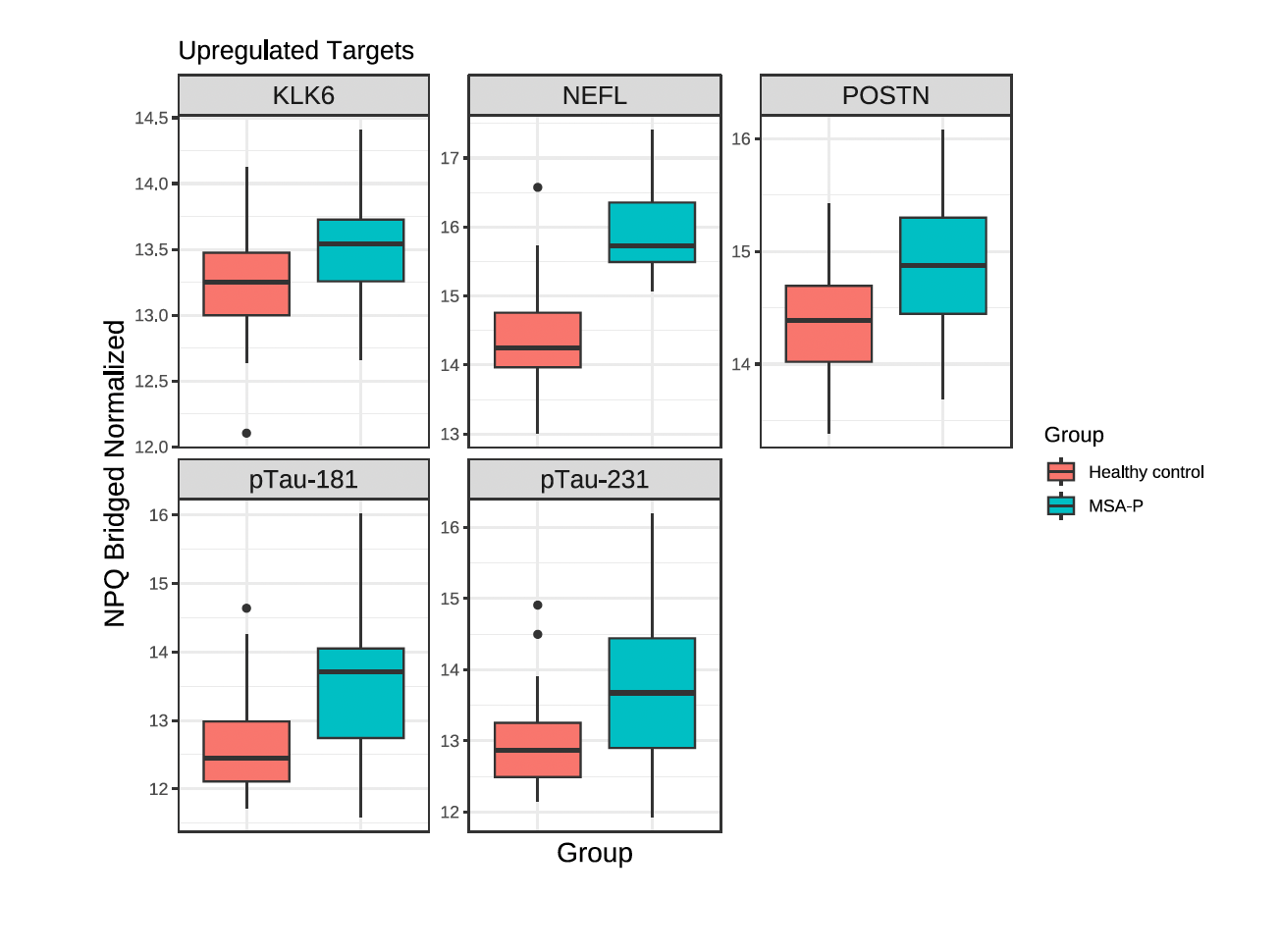


Supplementary Figure 9. MSA-P serum samples (n=22) taken at baseline trial visits compared to Healthy Control serum samples (n=39) using a categorical linear model adjusting for age at baseline and sex. Boxplots showing relative downregulated protein NPQ values in MSA-P serum samples. Boxplots show median, interquartile range, and whiskers show minimum and maximum values. Additional dots show outlying values.


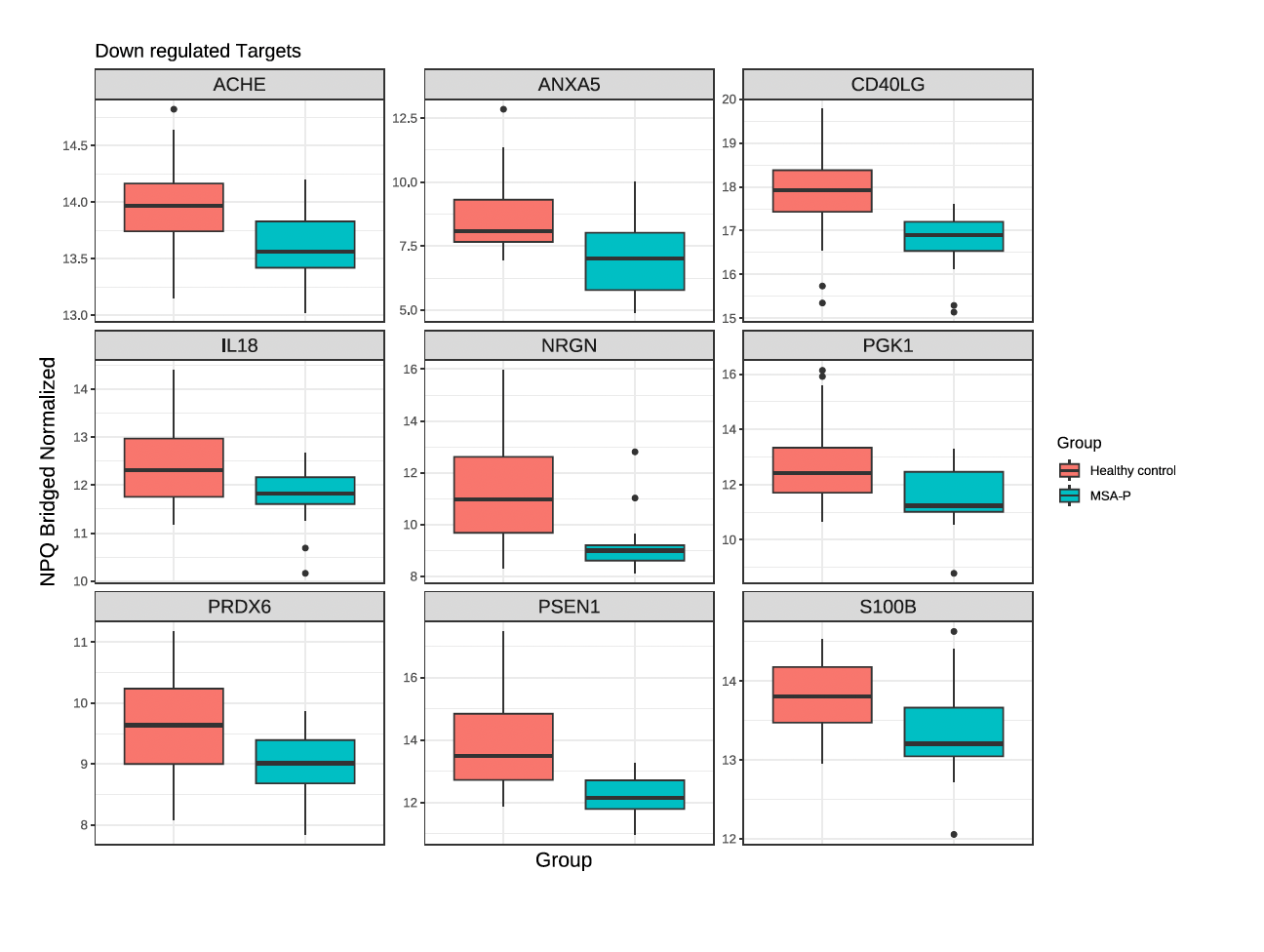


Supplementary Figure 10. MSA-C serum samples (n=20) taken at baseline trial visits compared to Healthy Control serum samples (n=39). using a categorical linear model adjusting for age at baseline and sex. The volcano plot shows Log_10_ adjusted FDR adjusted p values represented on the y axis i.e. a Log_10_ adjusted FDR adjusted p value of 5 represents p=0.00001. The x axis shows the log_2_ fold change, i.e. positive fold changes (proteins displayed in red) of +1 indicate that the protein values are twice as high in the MSA-C samples compared with control samples.


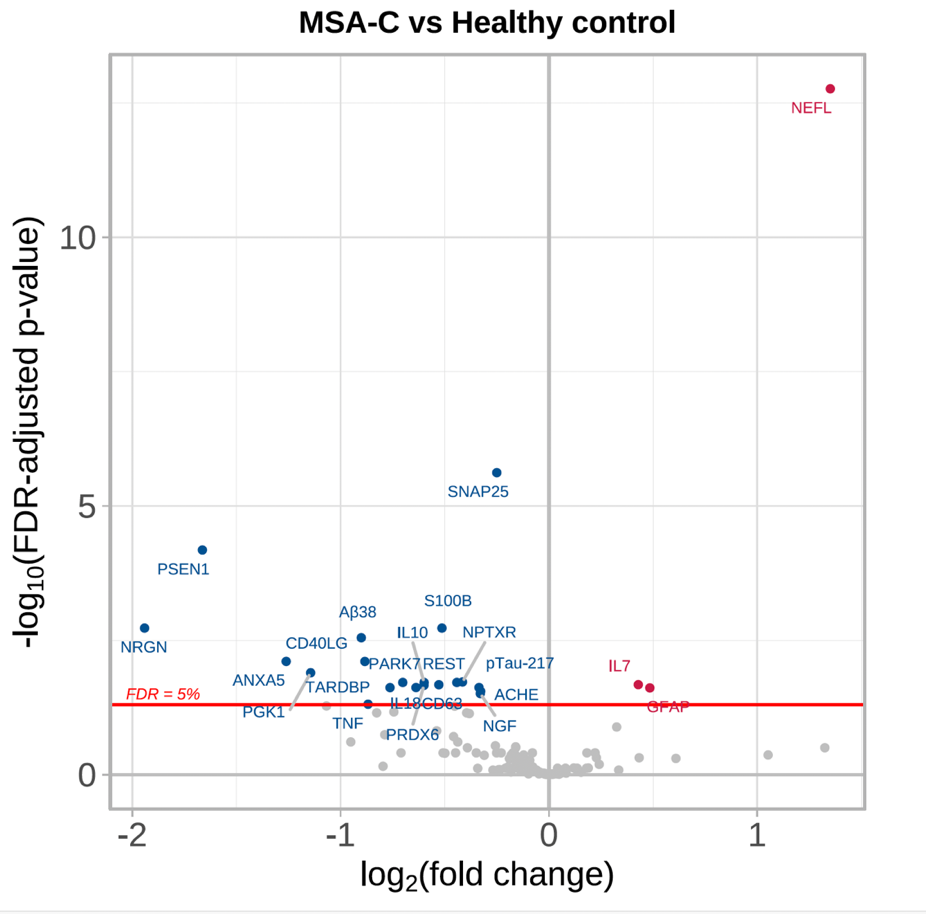


Supplementary Figure 11. MSA-C serum samples (n=22) taken at baseline trial visits compared to Healthy Control serum samples (n=39) using a categorical linear model adjusting for age at baseline and sex. Boxplots showing relative upregulated protein NPQ values in MSA-C serum samples. Boxplots show median, interquartile range, and whiskers show minimum and maximum values. Additional dots show outlying values.


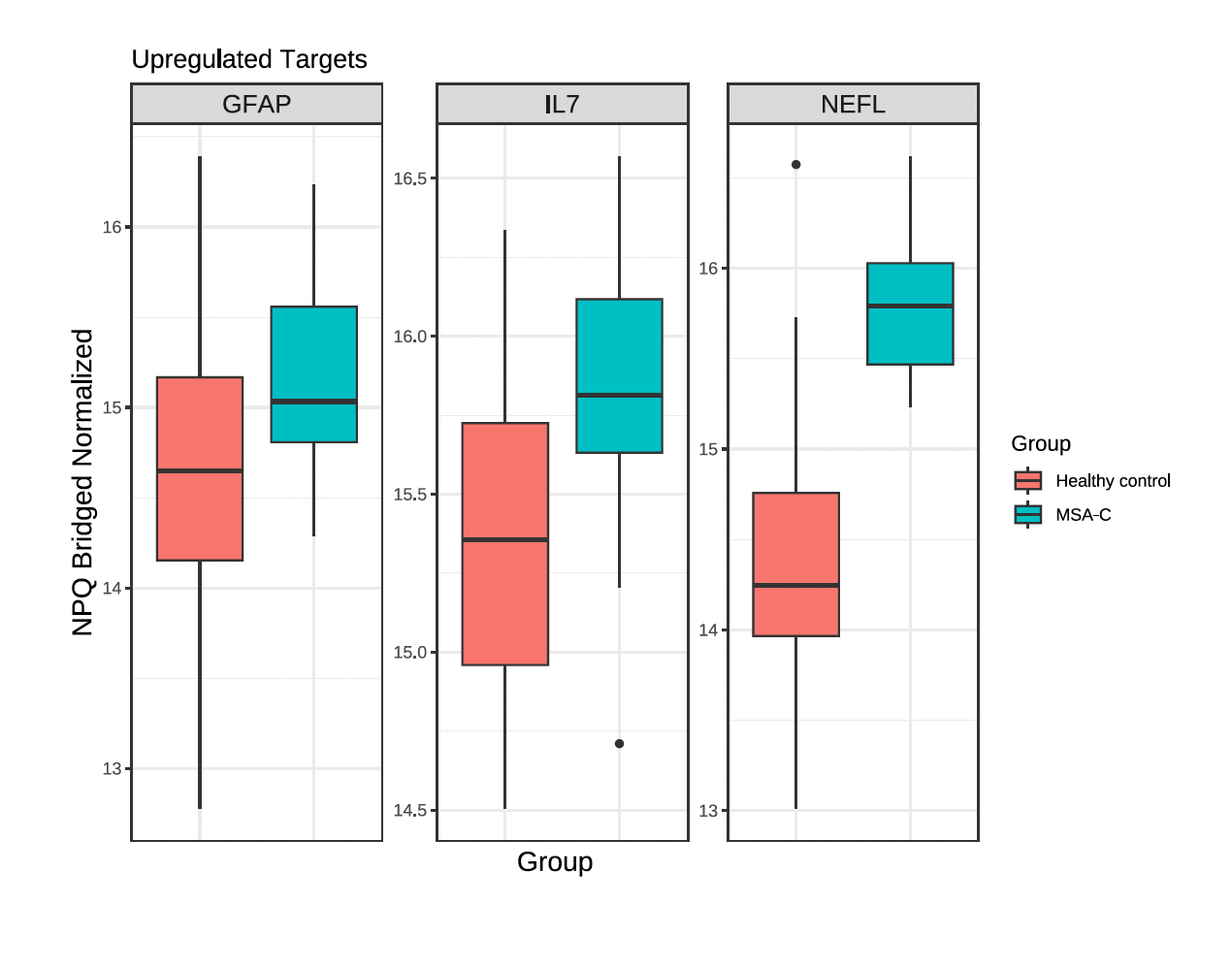


Supplementary Figure 12. MSA-C serum samples (n=22) taken at baseline trial visits compared to Healthy Control serum samples (n=39) using a categorical linear model adjusting for age at baseline and sex. Boxplots showing relative downregulated protein NPQ values in MSA-C serum samples. Boxplots show median, interquartile range, and whiskers show minimum and maximum values. Additional dots show outlying values.


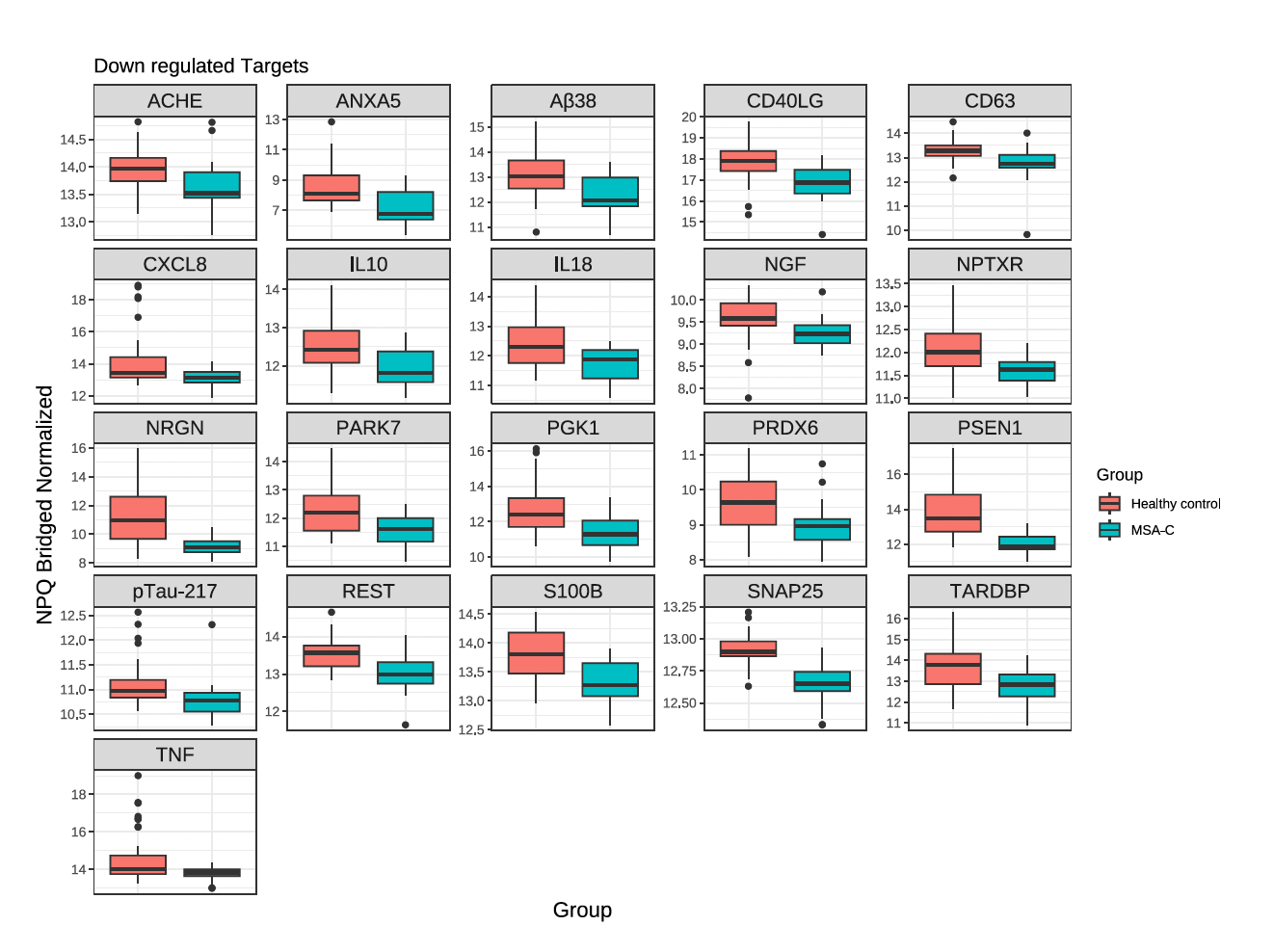


Supplementary figure 13. MSA Serum samples (n=42) collected at multiple timepoints and compared to MSA severity (UMSARS 1+2 score) using linear mixed-effects regression modelling, adjusting for age at baseline, sex, and exenatide drug randomisation, and a random effect for subject to account for within-subject correlation. Spaghetti plots showing individual variations in MSA serum NPQ values at different time points on x axis against disease severity score on y axis.


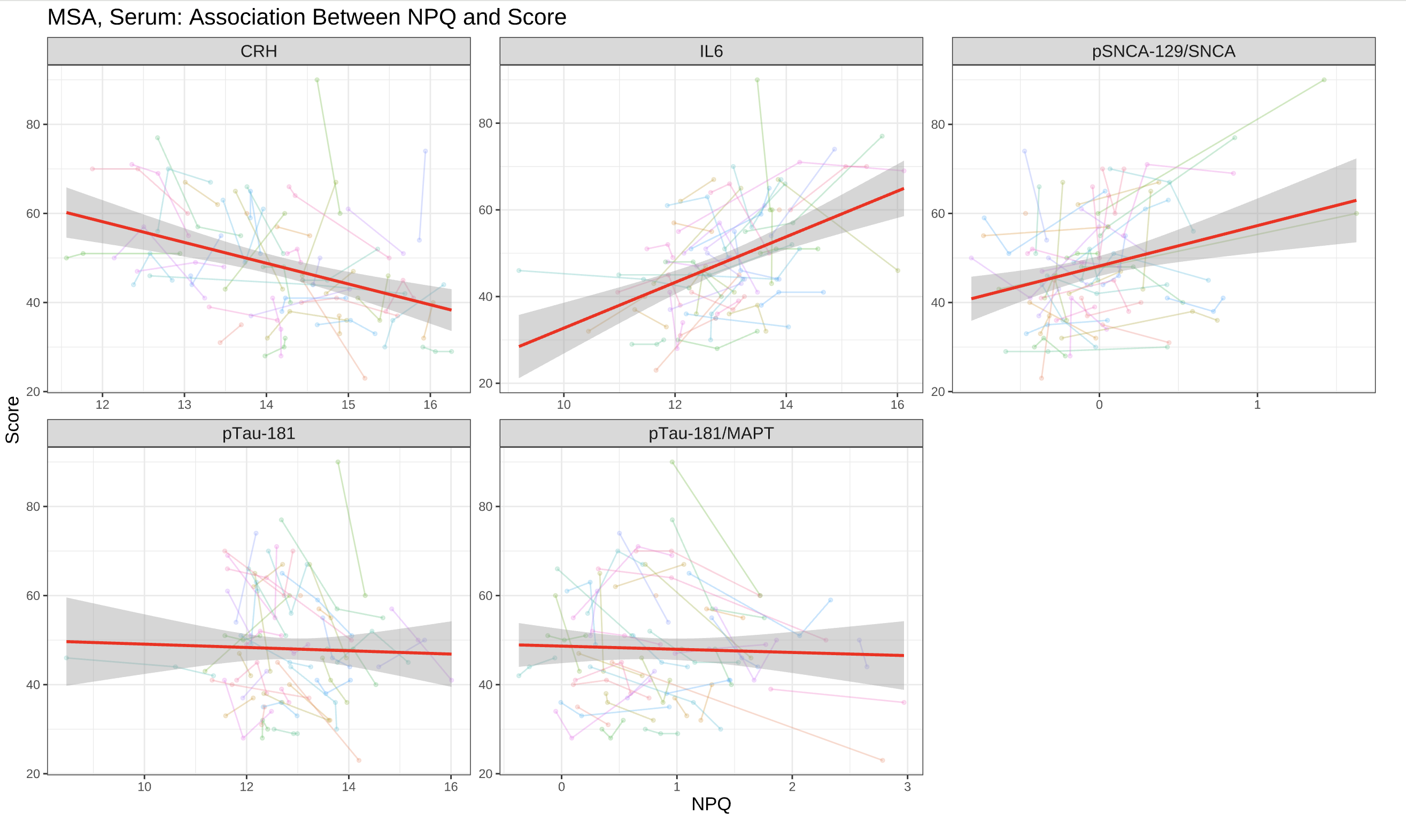


Supplementary Figure 14. MSA-P Serum samples (n=22) collected at multiple timepoints and compared to MSA-P severity (UMSARS part 1+2 score) using linear mixed-effects regression modelling, adjusting for age at baseline, sex, and exenatide drug randomisation, and a random effect for subject to account for within-subject correlation. The volcano plot shows Log_10_ adjusted FDR adjusted p values are represented on the y axis i.e. a Log_10_ adjusted FDR adjusted p value of 2 represents p=0.01. The x axis shows the direction of effect, i.e. positive coefficients (proteins displayed in red) indicate that higher protein values are significantly associated with worse disease severity.


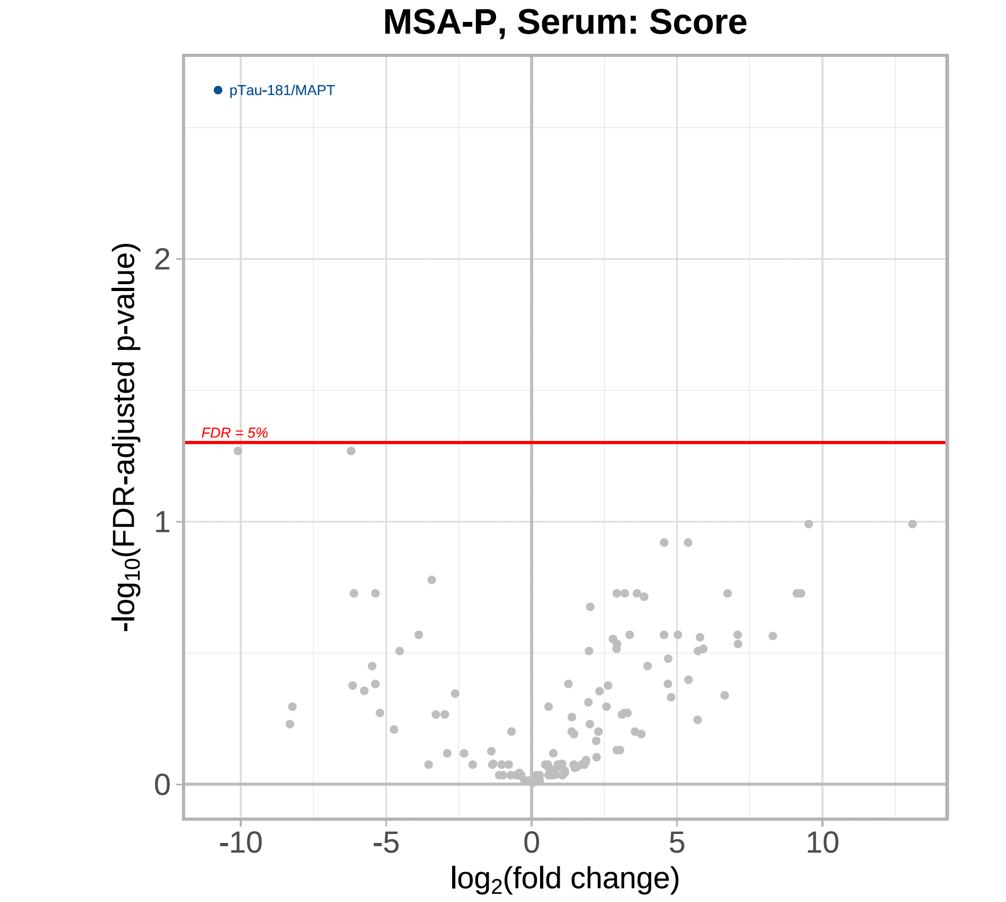


Supplementary Figure 15. MSA-P Serum samples (n=22) collected at multiple timepoints and compared to MSA-P severity (UMSARS part 1+2 score) using linear mixed-effects regression modelling, adjusting for age at baseline, sex, and exenatide drug randomisation, and a random effect for subject to account for within-subject correlation. Spaghetti plot showing individual variations in NPQ values over time on x axis against disease severity score on y axis.


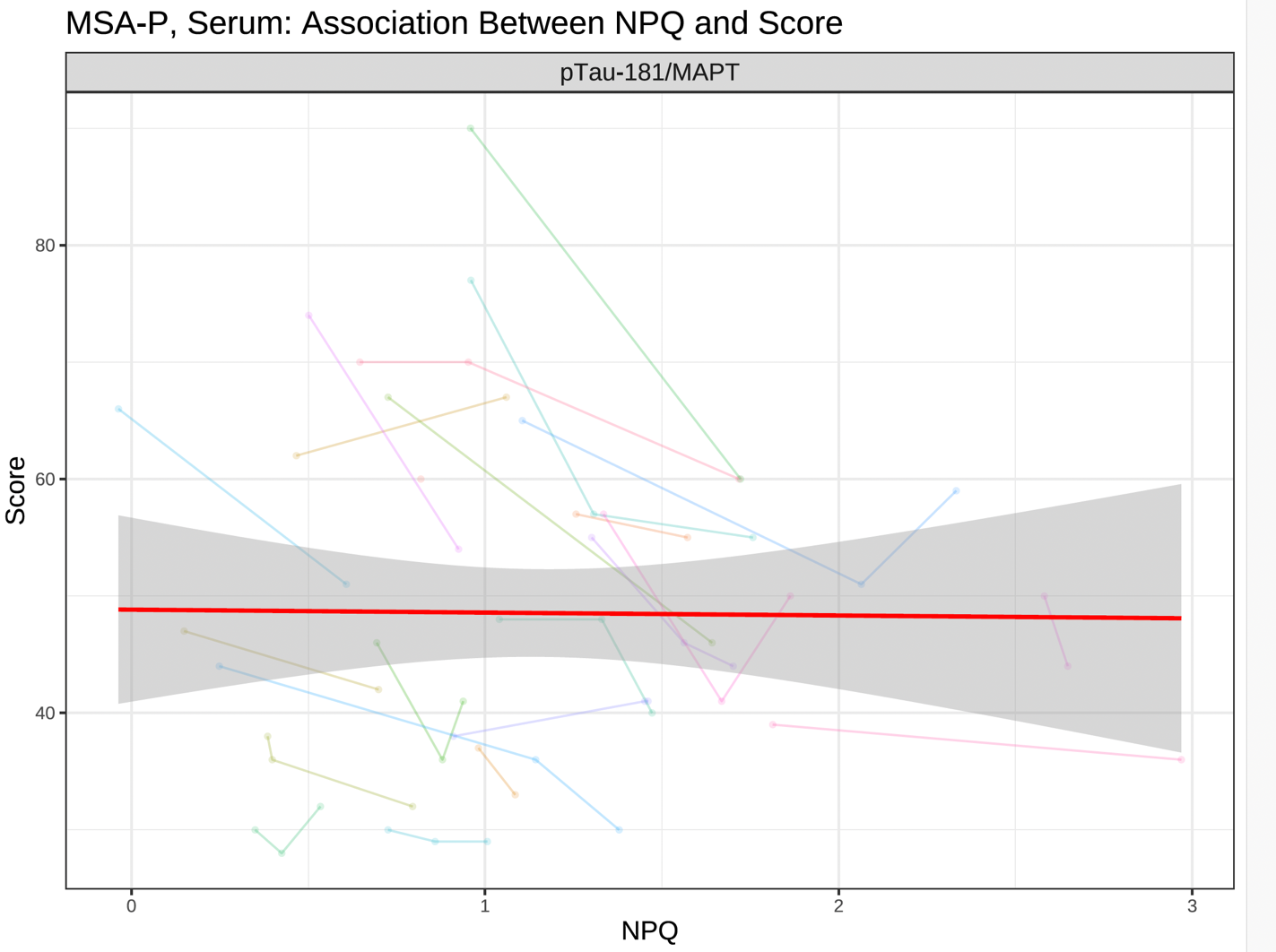


Supplementary Figure 16. MSA-C Serum samples (n=20) collected at multiple timepoints and compared to MSA-C severity (UMSARS part 1+2 score) using linear mixed-effects regression modelling, adjusting for age at baseline, sex, and exenatide drug randomisation, and a random effect for subject to account for within-subject correlation. The volcano plot shows Log_10_ adjusted FDR adjusted p values are represented on the y axis i.e. a Log_10_ adjusted FDR adjusted p value of 2 represents p=0.01. The x axis shows the direction of effect, i.e. positive coefficients (proteins displayed in red) indicate that higher protein values are significantly associated with worse disease severity.


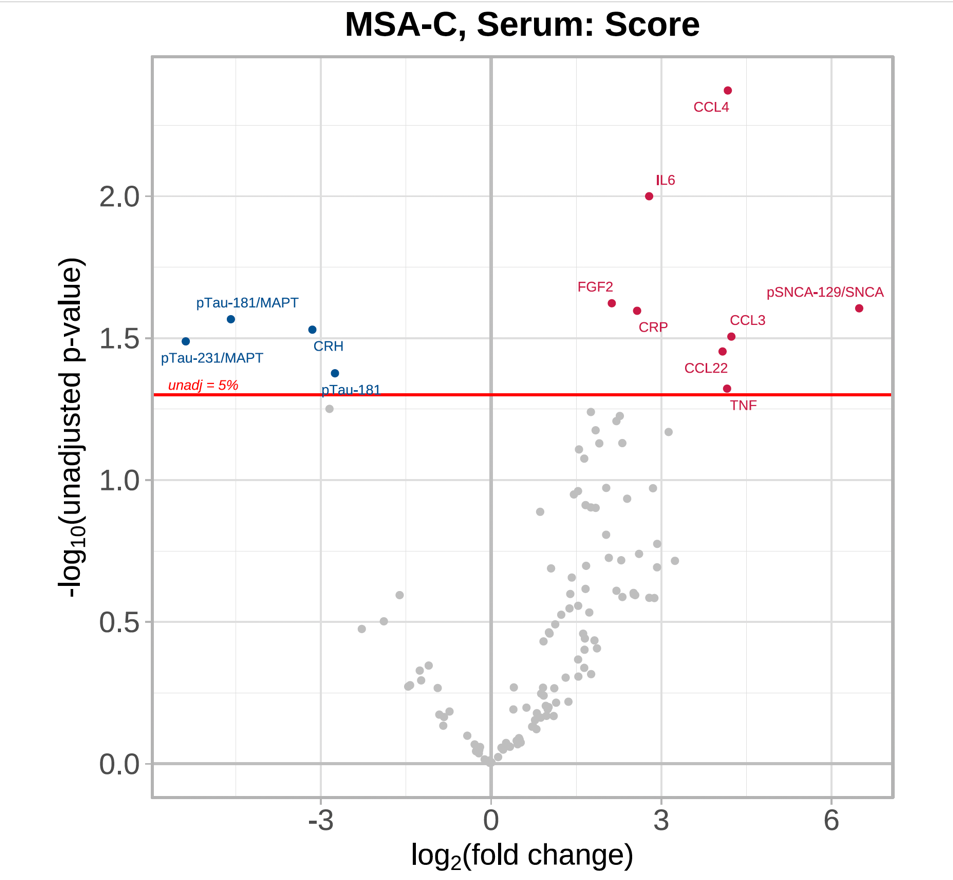


Supplementary Figure 17. MSA-C Serum samples (n=22) collected at multiple timepoints and compared to MSA-C severity (UMSARS part 1+2 score) using linear mixed-effects regression modelling, adjusting for age at baseline, sex, and exenatide drug randomisation, and a random effect for subject to account for within-subject correlation. Spaghetti plot showing individual variations in NPQ values over time on x axis against disease severity score on y axis.


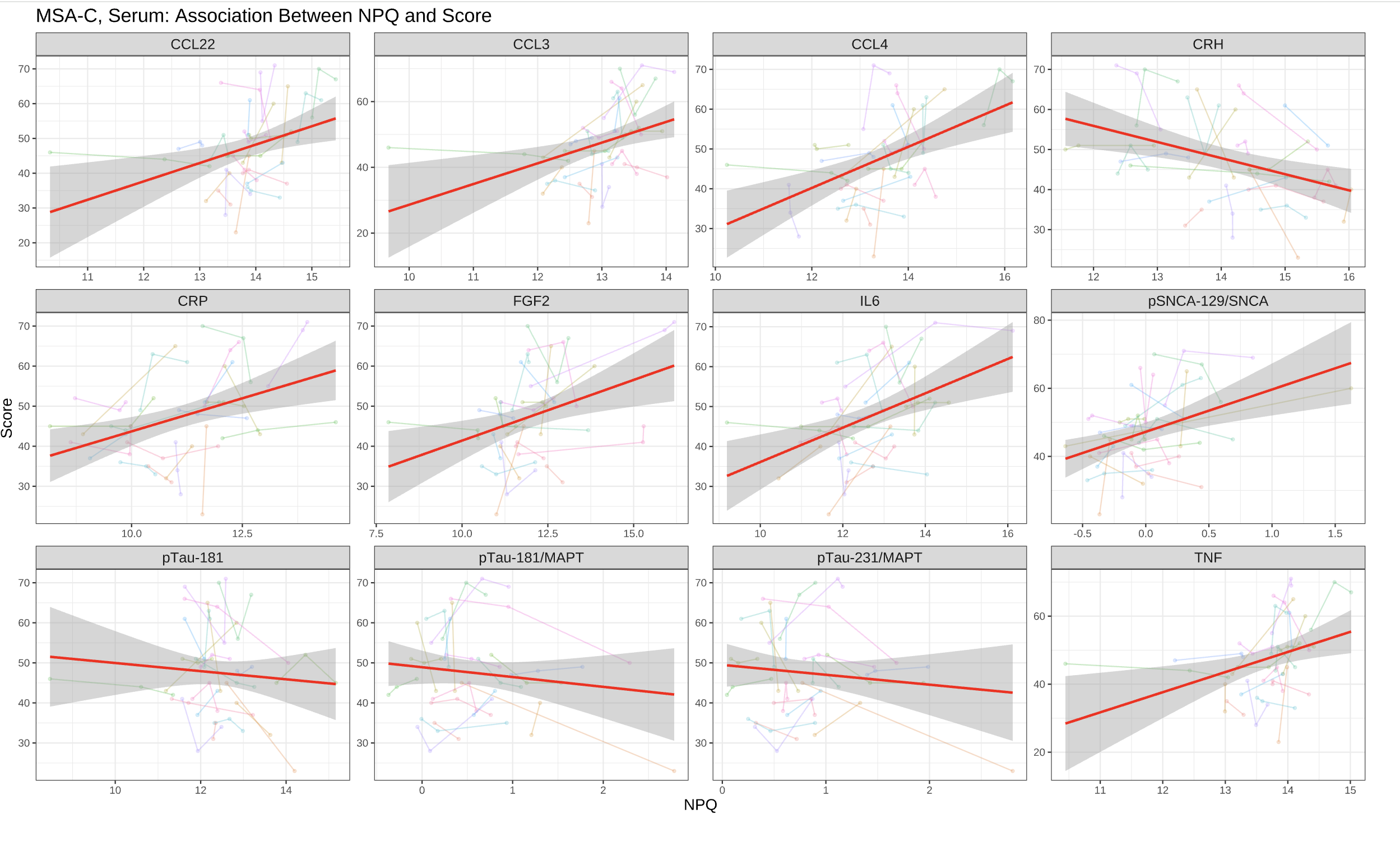


Supplementary Figure 18. Comparison of NPQ levels between serum and plasma samples taken contemporaneously from identical healthy controls. Boxplots showing relative protein NPQ values in healthy control serum and healthy control plasma. Boxplots show median, interquartile range, and whiskers show minimum and maximum values. Additional dots show outlying values.

Supplementary Figure 19. Comparison of serum and plasma samples taken contemporaneously from healthy controls, Boxplots showing relative protein NPQ values in healthy control serum and healthy control plasma restricted to those NPQ values significantly different values (FDR p<0.05).

Supplementary Figure 20. Magnitude of differential protein expression in MSA compared to PD in CSF using a categorical linear model adjusting for age at baseline and sex. PD CSF samples (n=51) compared to MSA CSF samples (n=23) collected at trial baseline visits. Boxplots showing relative upregulated protein NPQ values in MSA CSF samples relative to PD CSF samples.


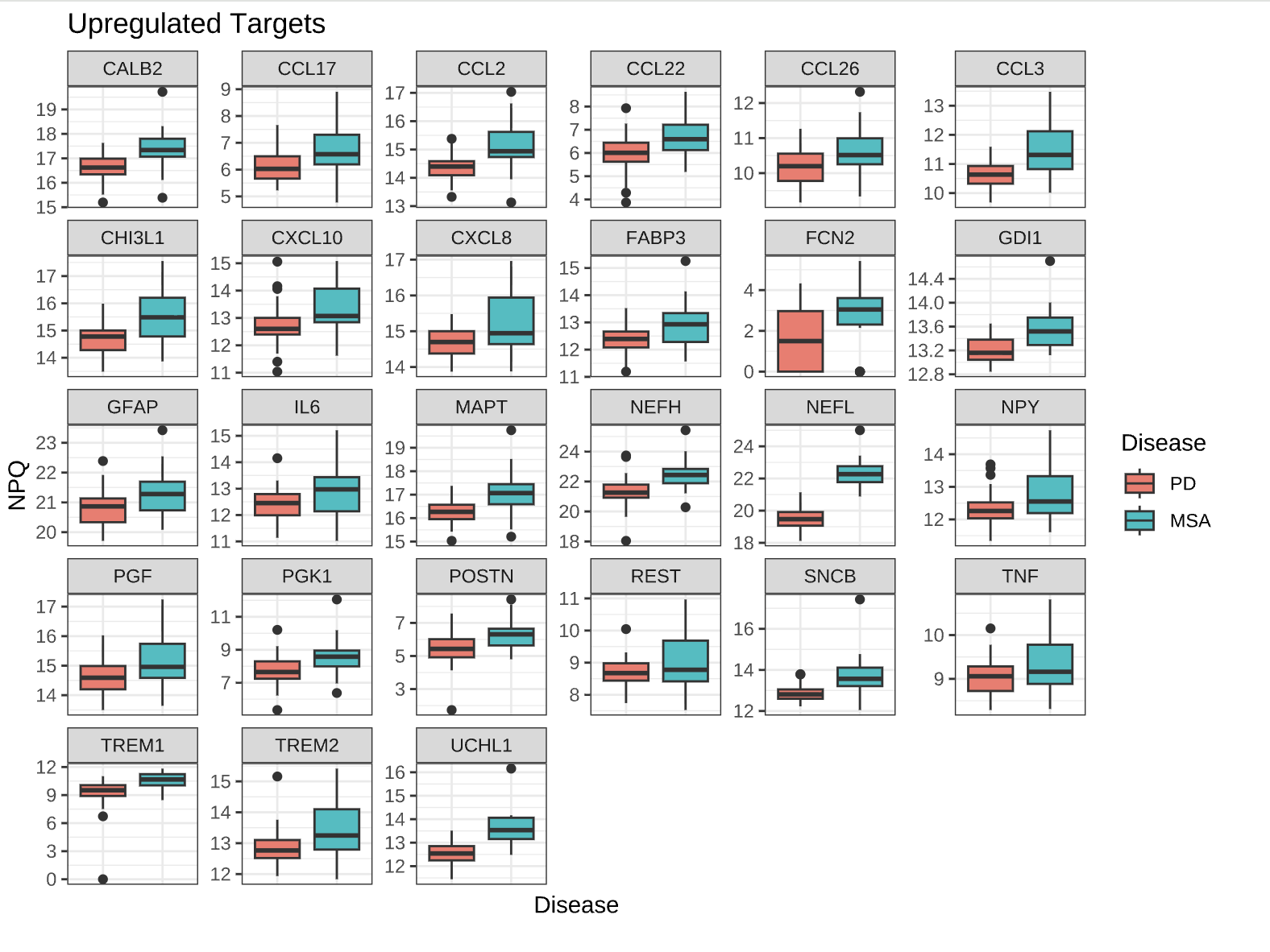


Supplementary Figure 21. PD CSF samples (n=51) compared to MSA-P CSF (n=13) samples using a categorical linear model adjusting for age at baseline and sex. Volcano plot shows Log_10_ adjusted FDR adjusted p values represented on the y axis i.e. a Log_10_ adjusted FDR adjusted p value of 5 represents p=0.00001. The x axis shows the log_2_ fold change, i.e. positive fold changes (proteins displayed in red) of +1 indicate that the protein values are twice as high in the MSA-P samples compared with PD samples.


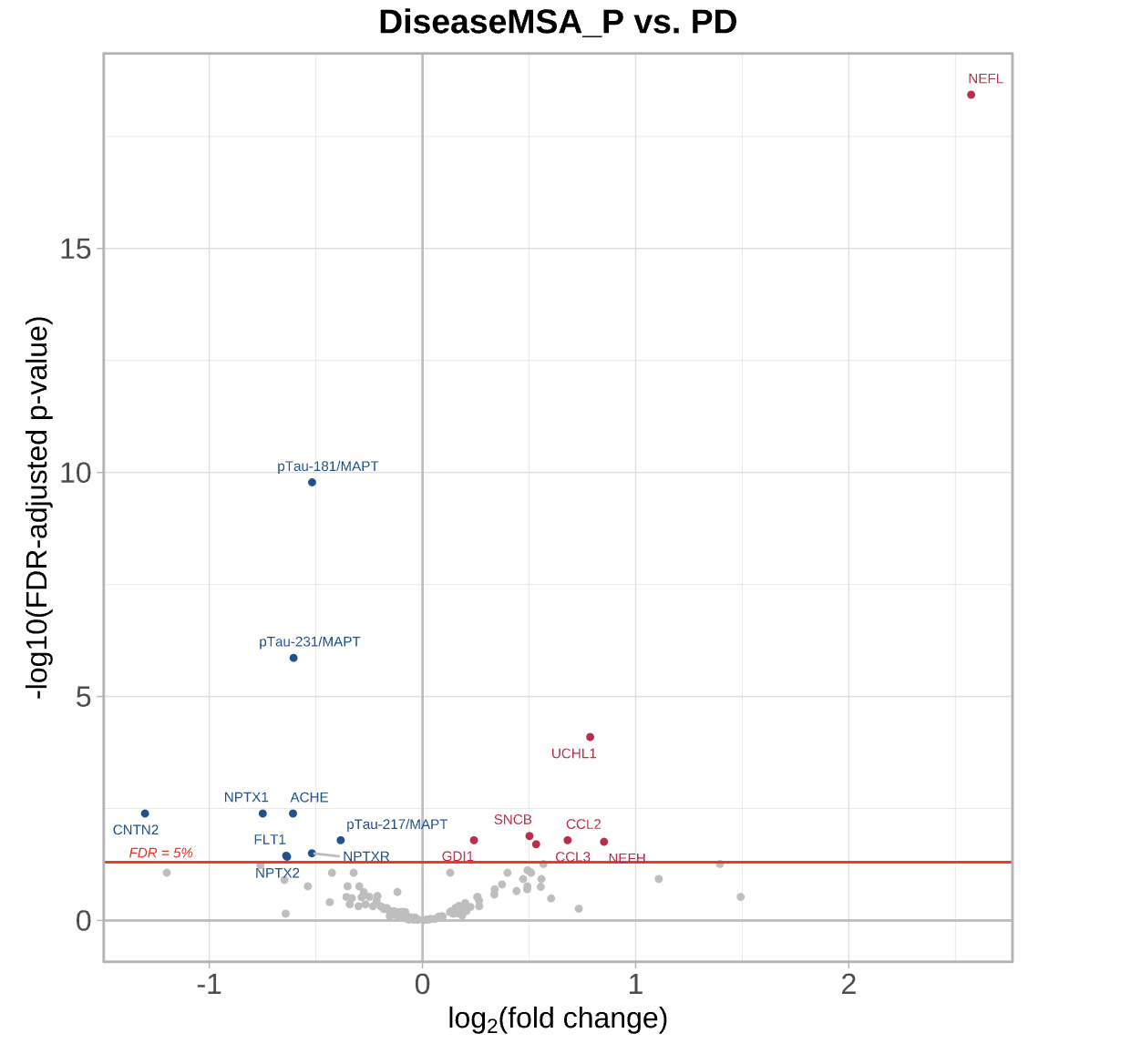


Supplementary Figure 22. PD CSF samples (n=51) compared to MSA-P CSF (n=13) samples using a categorical linear model adjusting for age at baseline and sex. Boxplots showing relative upregulated protein NPQ values in MSA-P compared to PD CSF samples.


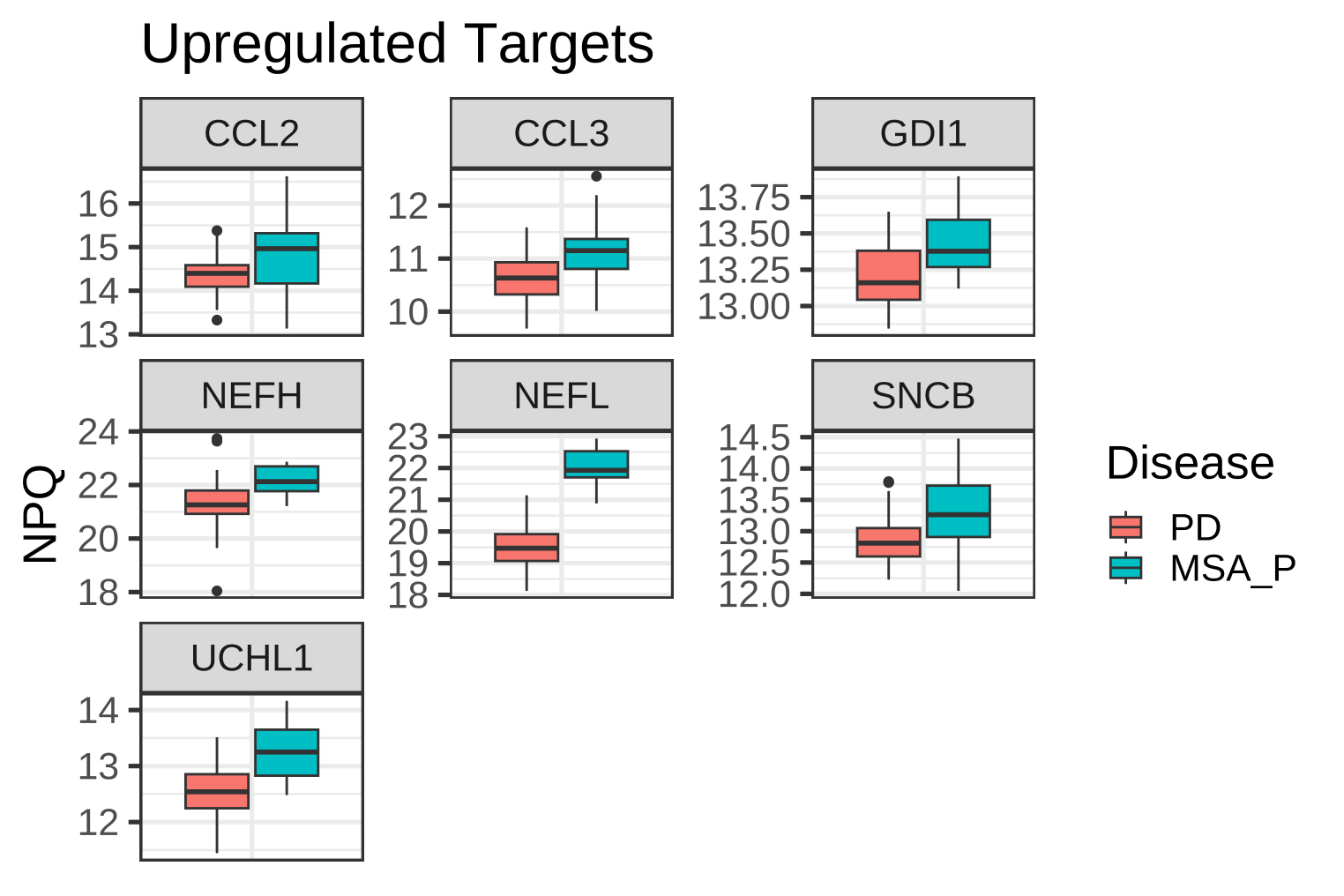


Supplementary Figure 23. PD CSF samples (n=51) compared to MSA-C CSF (n=10) samples using a categorical linear model adjusting for age at baseline and sex. The volcano plot shows Log_10_ adjusted FDR adjusted p values represented on the y axis i.e. a Log_10_ adjusted FDR adjusted p value of 5 represents p=0.00001. The x axis shows the log_2_ fold change, i.e. positive fold changes (proteins displayed in red) of +1 indicate that the protein values are twice as high in the MSA-C samples compared with PD samples.


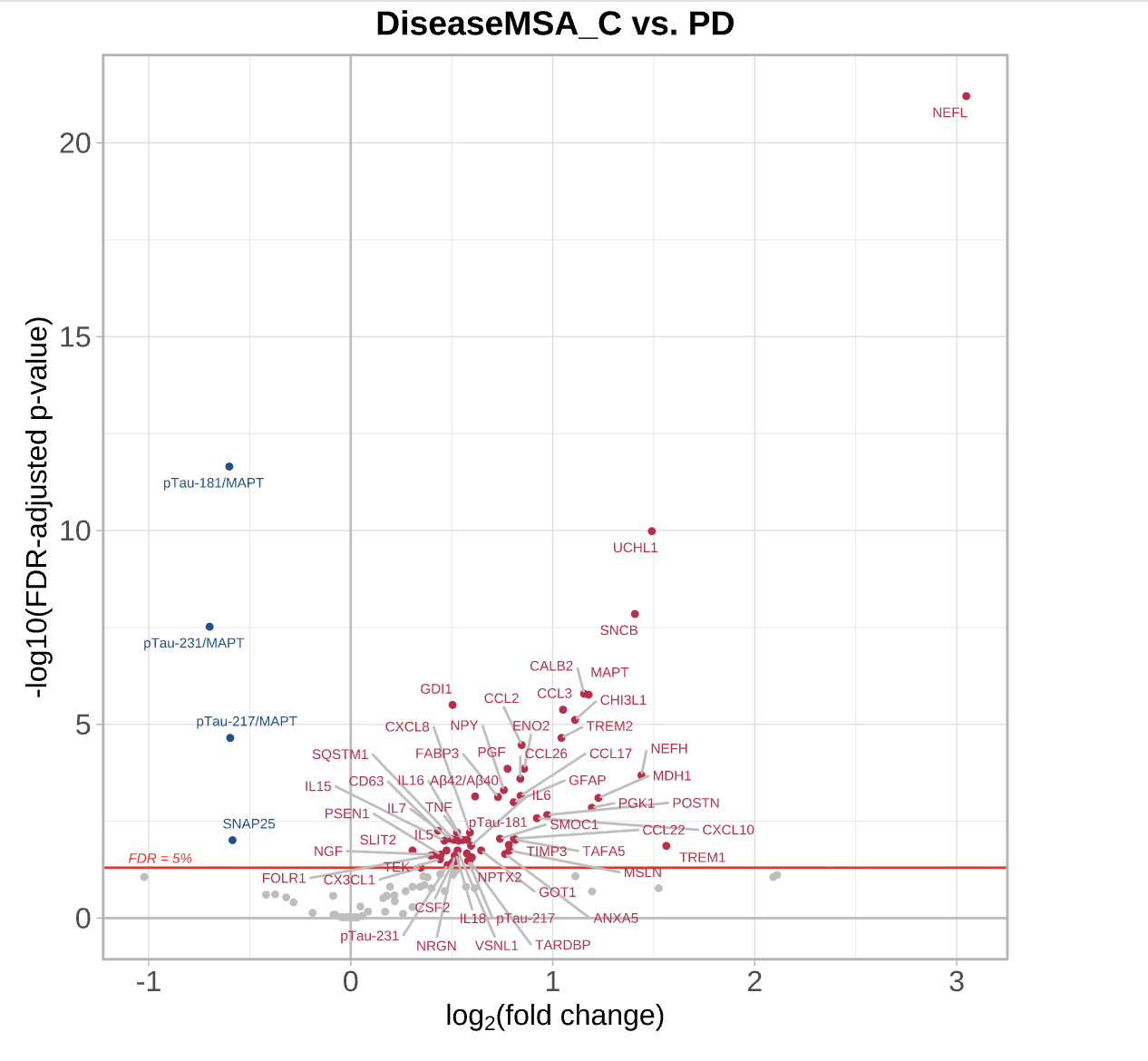


Supplementary Figure 24. PD CSF samples (n=51) compared to MSA-C CSF (n=10) samples using a categorical linear model adjusting for age at baseline and sex. Boxplots showing relative upregulated protein NPQ values in MSA-C compared to PD CSF samples.


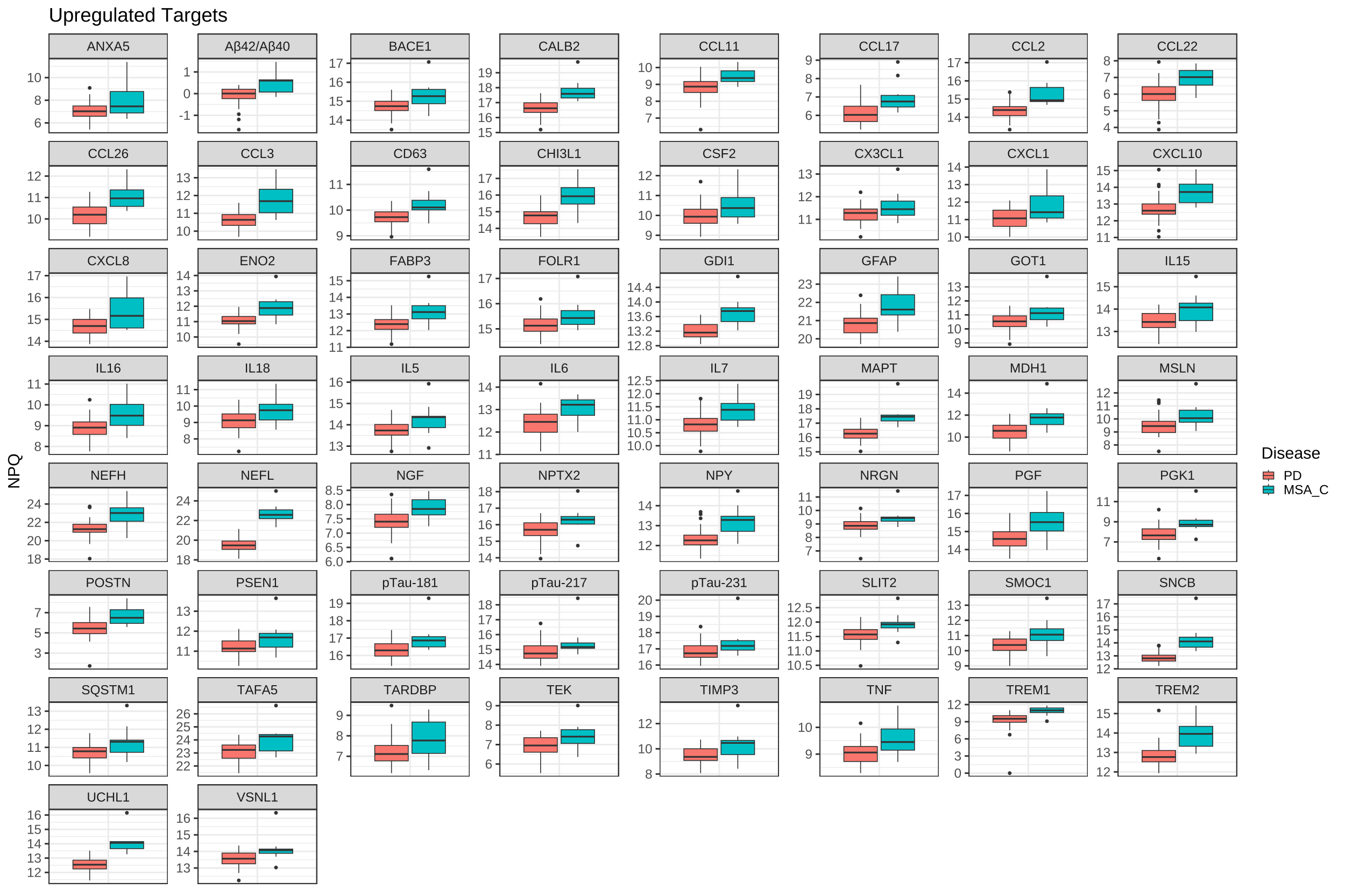


Supplementary Figure 25. Magnitude of differential protein expression in MSA compared to PD in CSF using a categorical linear model adjusting for age at baseline and sex. PD CSF samples (n=51) compared to MSA CSF samples (n=23) collected at trial baseline visits. Boxplots showing relative downregulated protein NPQ values in MSA CSF samples relative to PD CSF samples.


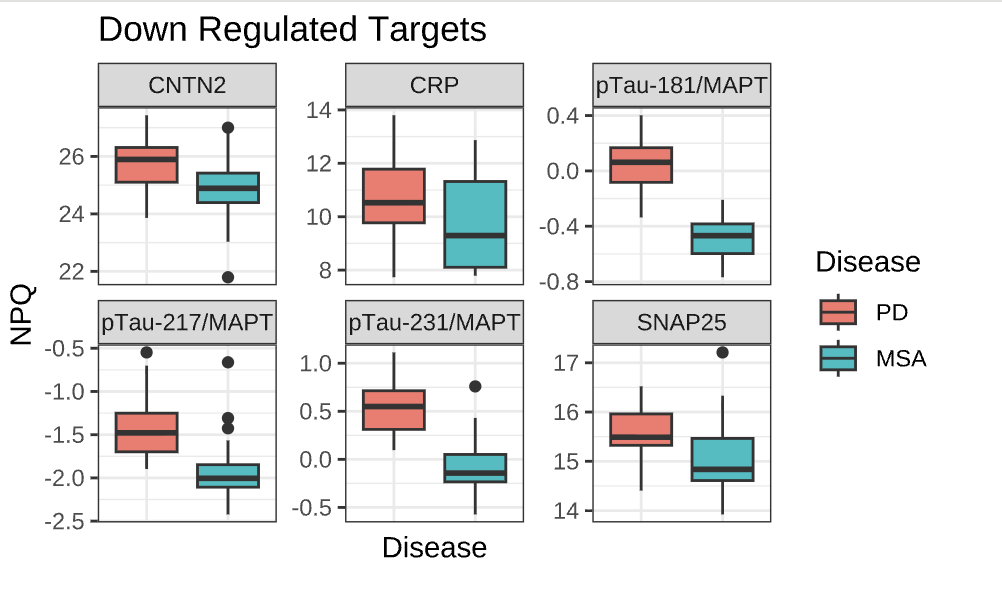


Supplementary Figure 26. Magnitude of differential protein expression in MSA-P compared to PD in CSF using a categorical linear model adjusting for age at baseline and sex. PD CSF samples (n=51) compared to MSA-P CSF samples (n=13) collected at trial baseline visits. Boxplots showing relative downregulated protein NPQ values in MSA-P CSF samples relative to PD CSF samples.


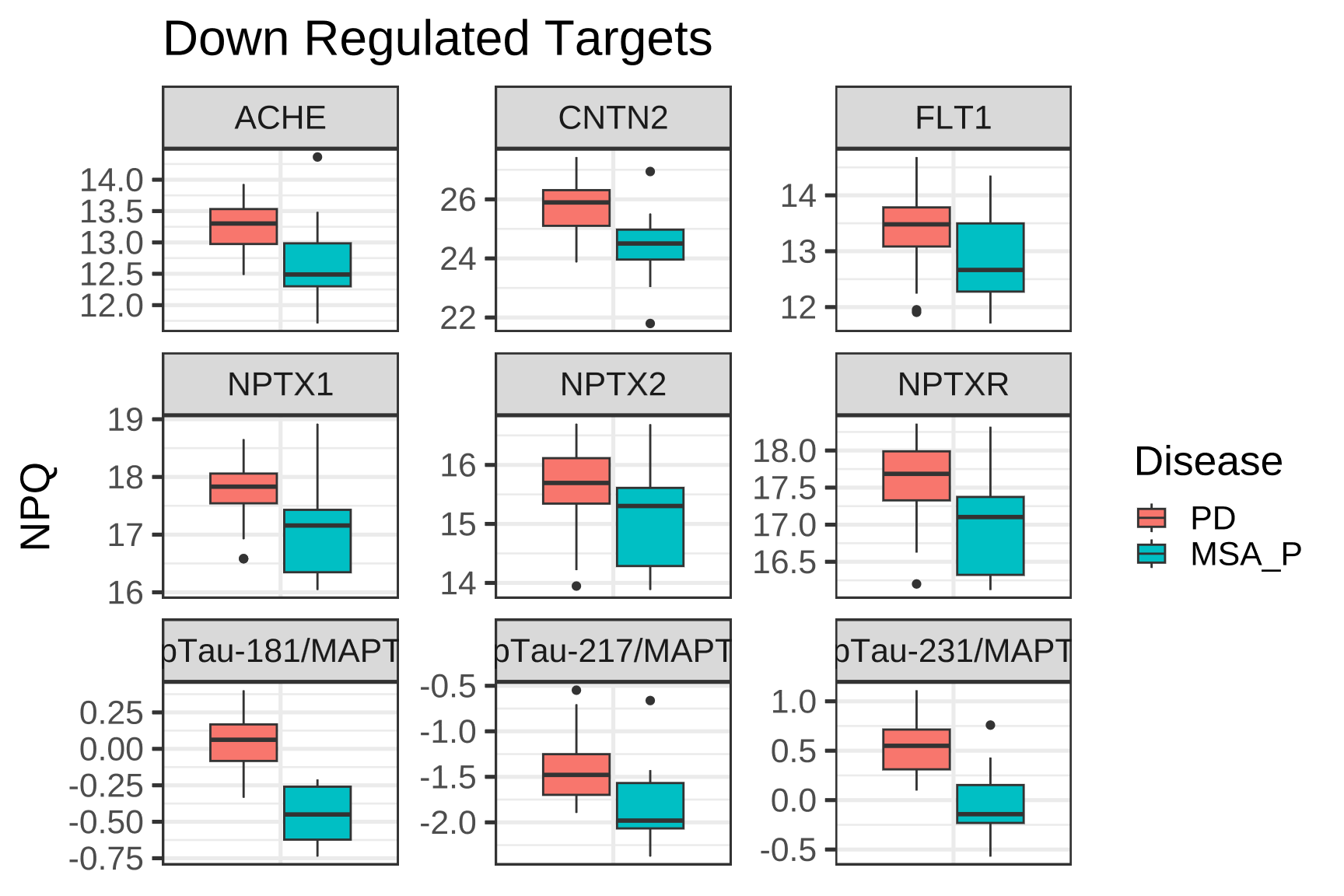


Supplementary Figure 27. Magnitude of differential protein expression in MSA-C compared to PD in CSF using a categorical linear model adjusting for age at baseline and sex. PD CSF samples (n=51) compared to MSA-C CSF samples (n=10) collected at trial baseline visits. Boxplots showing relative downregulated protein NPQ values in MSA-C CSF samples relative to PD CSF samples.


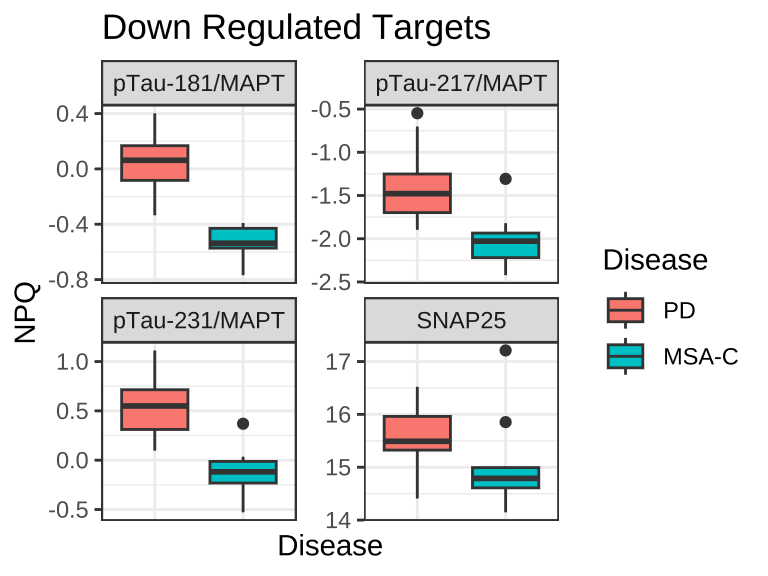

Supplement: fcag035_Supplementary_Data [file fcag035_supplementary_data.docx]
